# Supplementary material for: Understanding the roles and work of paramedics in primary care: a national cross-sectional survey
Source: BMJ Open. 2022 Dec 19;12(12):e067476. doi: 10.1136/bmjopen-2022-067476 (PMC9764645; doi:10.1136/bmjopen-2022-067476)
Supplement: Supplementary data [file bmjopen-2022-067476supp001.pdf]

SURVEY QUESTIONNAIRE

Understanding the roles and work of paramedics in primary care: A national cross-sectional survey

What is this project about?
Thank you for your interest in participating in this questionnaire. This questionnaire is part of a study which aims improve understanding of the ways in which paramedics are working within the primary care workforce in the United Kingdom. Whilst paramedics are currently working in primary care roles, evidence must be generated to show how and why these changes would work, for whom, in what context and to what extent. That's why we're keen to understand from your perspective, as a paramedic working in primary care, the impact you feel you have and your perspectives of your role. We hope that the results of our research will influence future policy and professional change, as well as support paramedics working in primary care in the future.

You have been invited to participate as you are a paramedic working in primary care within the UK. Please read through this information before agreeing to participate (if you wish to) by ticking the 'yes' box below.

You may ask any questions before deciding to take part by contacting the researcher (details below).

The Principal Researcher is Georgette Eaton, who is attached to the Nuffield Department of Primary Care Health Services at the University of Oxford. This project is being completed under the supervision of Dr Kamal R. Mahtani, Dr Geoff Wong, Dr Stephanie Tierney and Dr Veronika Williams from the University of Oxford, and Professor Julia Williams from the University of Hertfordshire. It is funded through a Doctoral Research Fellowship from the National Institute of Health Research.

You will be asked a series of questions about the work you undertake in primary care, and your perceptions of this. This should take about 20 minutes. No other background knowledge is required.

Do I have to take part?
No. Please note that participation is voluntary. If you do decide to take part, you may withdraw at any point for any reason before submitting your answers by pressing the 'Exit' button/ closing the browser. We are offering every paramedic who completes the questionnaire a £10 Amazon e-voucher. However, we are only able to offer this to participants who complete all study activities.

We have included a 'Prefer not to say' option for each set of questions should you prefer not to answer a particular question.

How will my data be used?
During the main set of questions, we will not collect any data that could directly identify you.

At the end of the survey, you will see a page to register your interest in being part of the second phase of our research which will involve an interview. To register your interest, please submit your name and nhs.net email address. This is not linked to your completion of the questionnaire and there is no obligation to be involved in the second phase of our research.

We are offering every paramedic who completes the questionnaire a £10 Amazon e-voucher. At the end of the survey, you will be taken to a separate page to give us your

SURVEY QUESTIONNAIRE

name and nhs.net email address in order to claim this e-voucher. This cannot be matched to your responses in the survey and will be destroyed when individual participant involvement is complete (e.g. when the Amazon e-voucher has been sent and redeemed by you.

Your IP address will not be stored. We will take all reasonable measures to ensure that data remain confidential.

The responses you provide will be stored in a password-protected electronic file on University of Oxford secure servers and may be used in academic publications, conference presentations, reports for external organisations and on websites. Identifiable information will be deleted as soon as it is no longer required for the research. Research data will be stored for 3 years after publication or public release of the study's results.

Who will have access to my data?
The University of Oxford is the data controller with respect to your personal data (name and email address) and, as such, will determine how your personal data is used in the study. The University will process your personal data for the purpose of the research reasons outlined above. Research is a task that we perform in the public interest. Further information about your rights with respect to your personal data is available from https://compliance.admin.ox.ac.uk/individual-rights.

All data will be anonymous. The answers you give us will be shared with the College of Paramedics, Health Education England, and the National Institute for Health Research.

We would also like your permission to use the questionnaire answers in future studies, and to share these with other researchers (e.g., in online databases). This will not include your name and email address, as this is only to claim incentive for the study or to participate in future work associated with this project.

The results will be written up for a DPhil (PhD) degree.

Who has reviewed this study?
This project has been reviewed by, and received ethics clearance through, a subcommittee of the University of Oxford Central University Research Ethics Committee R64129/RE001.

Who do I contact if I have a concern or I wish to complain?
If you have a concern about any aspect of this study, please speak to Georgette Eaton at georgette.eaton@phc.ox.ac.uk or their supervisor, Professor Kamal R. Mahtani kamal.mahtani@phc.ox.ac.uk and we will do our best to answer your query. We will acknowledge your concern within 10 working days and give you an indication of how it will be dealt with. If you remain unhappy or wish to make a formal complaint, please contact the Chair of the Research Ethics Committee at the University of Oxford who will seek to resolve the matter as soon as possible.

Please note that you may only participate in this survey if you are 18 years of age or over	Yes<br>No
If you have read the information above and agree to participate with the understanding that the data (including personal data) you submit will be processed accordingly, please tick the box below to start.	Yes<br>No

SURVEY QUESTIONNAIRE

**Section 1.** The purpose of this section is to find out about the prevalence of paramedics working in primary care in the United Kingdom.	
Are you currently a paramedic working in primary care?	Yes<br>No
What is your age range?	18-24 years old<br>25-34 years old<br>35-44 years old<br>45-54 years old<br>55-65 years old
Which of the following most accurately describe(s) you?<br><br>(Multiple choice answer)	Female<br>Intersex<br>Male<br>Non-binary<br>Transgender<br>Let me type (answer box)
Where do you practice?	England<br>Northern Ireland<br>Scotland<br>Wales
How long have you been a registered paramedic?	0-2 years<br>3-5 years<br>6-10 years<br>11- 14 years<br>15 - 19 years<br>20-24 years<br>25 - 30 years<br>31-35 years<br>>36 years
How long have you worked in your current role in primary care?	0-6 months<br>6-12 months<br>12 – 18months<br>18months – 2 years<br>2-4 years<br>5-7 years<br>8-10 years<br>11- 14 years<br>15 - 19 years<br>>20 years
What is your salary?	Band 5 (£24,907 - £30,615)<br>Band 6 (£31,365 - £37,890)<br>Band 7 (£38,890 - £44,503)<br>Band 8a (£45,753 - £51,668)<br>Band 8b (£53,168 - £62,001)<br>Band 8c (£63,751 - £73,664)<br>Band 8d (£75,914 - £87,754)<br>Band 9 (£91,004 - £104,927)

SURVEY QUESTIONNAIRE

What hours do you work in primary care?	Full time – more than 30 hours per week<br>Part time – 21-30 hours per week<br>Part time – 10-20 hours per week<br>One day a week<br>One session a week (4 hours)
What is your highest qualification relevant to your role as a paramedic in primary care?	Institute of Health and Care Development (IHCD) Graduate Diploma<br>Diploma/Certificate of Higher Education<br>Foundation degree<br>Bachelor's degree<br>Practice Certificate<br>Postgraduate Certificate<br>Postgraduate Diploma<br>Master's Degree<br>Let me type (answer box)
What is your job title?	Paramedic<br>Specialist Paramedic<br>Paramedic Practitioner<br>First Contact Practitioner (Paramedic)<br>Emergency Care Practitioner<br>Advanced Clinical Practitioner (Paramedic)<br>Advanced Paramedic<br>Consultant Paramedic<br>Let me type (answer box)
Are you annotated as an independent prescriber with the Health and Care Professions Council?	Yes<br>Not yet – I am undertaking the course currently<br>No – but I would like to be in the future<br>No
How are you employed in primary care?	Directly employed by GP practice<br>Directly employed by a primary care network/federation/health board<br>Freelance/locum contract<br>Agency/Private provider<br>Rotational through ambulance trust<br>Let me type (answer box)
What type of tasks do you undertake*?<br><br>*These could be face to face or remotely (by telephone or online)<br><br>Please select as many as apply.	Same day/urgent home visits<br>Routine home visits<br>Same day/urgent clinic appointments*<br>Minor illness clinic*<br>Routine clinic appointments*<br>Telephone triage<br>Care home ward rounds<br>Chronic Disease Reviews*<br>Covid clinics*

SURVEY QUESTIONNAIRE

	Admin (e.g. requesting investigations, interpreting results, medication checks)<br>Let me type (answer box)
What types of patients do you see?	Let me type (answer box)
Are there any patient types that you do not see?	Let me type (answer box)
Why?	Let me type (answer box)

SURVEY QUESTIONNAIRE

Section 2.
The purpose of this section is to understand the type of activities that make up your role as a paramedic in primary care. For each of the following activities, please indicate the extent that these form part of your role

	Not at all	Little extent	Some extent	Great extent	Very great extent
Communication and consultations					
Practising holistically to personalise care and promote public and person health					
Working with colleagues in primary care					
Maintaining an ethical approach and fitness to practice					
Information gathering and interpretation					
Clinical Examination and procedural skills					
Making a diagnosis					
Managing medical and clinical complexity					
Independent prescribing, medicines, and supply of pharmacotherapy					
Leadership and Management					
Education and Development					
Research and evidence-based practice					

SURVEY QUESTIONNAIRE

Section 3.
The purpose of this section is to understand the type of clinical presentations that make up your role as a paramedic in primary care. For each of the following activities, please tick the regularity of which you see these in your role in primary care

	Not at all	Sometimes	Regularly	Very regularly	All the time
**Cardiovascular system*** Blood pressure issues, chest pain, chest discomfort, orthopnoea, palpitation, irregular pulse, oedema, shortness of breath on exertion					
**Dermatology*** Rash, itching, infestation, spots, skin lesions/moles, nail issues, changes in pigmentation					
**Eyes, Ears, Nose and Throat*** Red eye, visual disturbance, acute loss of vision, eye discharge, eye injury, foreign body, swollen eyelid, dizziness, vertigo, otalgia, otorrhoea, sinus pain, nasal pain, nasal obstruction, mouth pain, neck swelling, sore throat, throat swelling, tinnitus, hearing loss, voice changes					
**Emergency presentations*** Cardiac arrest, catastrophic haemorrhage, shock, respiratory distress, cardiovascular emergency, anaphylaxis, angioedema, allergic reaction, collapse, seizure, non-blanching rash, overdose/poisoning, suspected DKA, meningism, limp child					
**Gastrointestinal system*** Difficulty swallowing, poor appetite, excessive thirst, abdominal pain, abdominal distention, abdominal mass/swelling, constipation, diarrhoea, change in bowel habit, nausea/vomiting, hematemesis, indigestion, rectal bleeding, abdominal blood results (eg. deranged liver enzymes, LFTs, anaemia), high risk behaviours and concerns, stoma issues					

SURVEY QUESTIONNAIRE

**Genitourinary System*** Loin pain, groin pain, haematuria, urinary symptoms, kidney disease, recurrent infection, penile pain, testicular pain/swelling, inability to pass urine, profuse vaginal bleeding, acute groin swelling/pain					
**General presentations*** Breast symptoms, tired all the time, generalised aches and pains, lymphadenopathy, sleep issues, fever, substance/alcohol misuse, overdose/poisoning, vulnerable adult, family/carer concern, genetic predisposition, presentations in patients with a learning disability; review of blood test results					
**Sexual health assessment*** Genital rashes/irritation, urinary symptoms, penile pain, penile discharge, acute groin swelling/pain, pelvic pain/mass, contraception					
**Medication Review*** Adverse side effects, ineffective medication, poor compliance, overuse of medication, misuse of medication, issues with polypharmacy, abnormal blood test results, higher risk groups (risk reduction medications)					
**Mental Health*** Suicidal ideation, self-harm, acute anxiety, stress, panic, post-natal mental health issues, visual/auditory hallucinations, paranoia, bereavement, substance misuse					
**Musculoskeletal system*** Pain, swelling, redness, stiffness, difficulty with movement, minor injury					
**Neurological system*** Altered level of consciousness, fits faints and funny turns, dizziness, altered power tone sensitivity, paraesthesia, altered level of consciousness,					

SURVEY QUESTIONNAIRE

weakness, altered gait, facial palsy, tremor, speech changes, headache, head injury, memory problems, confusion					
**Paediatrics*** Vulnerable child, rashes, pyrexia of unknown origin, crying baby, otalgia/otorrhoea, eye injury, red eye/discharge, cough/wheeze/stridor/respiratory distress/nasal symptoms, sore throat, vomiting, diarrhoea, acute bowel symptoms, abdominal pain, constipation, musculoskeletal symptoms, minor injury					
**Pain*** Acute pain, worsening pain, change in type of pain					
**Palliative and end of life care*** Symptom management, discussions about advanced care planning, verification of death					
**Pregnancy related conditions**					
**Non-pregnancy related conditions in pregnant people**					
**Respiratory System*** Shortness of breath, breathing difficulties, pain on breathing, cough (including haemoptysis), wheeze, pallor, cyanosis, suspected or recurrent infection, acute covid-19, post covid-19 syndrome					

SURVEY QUESTIONNAIRE

**Section 4.** The purpose of this section is to understand the type of clinical investigations and procedural skills that make up your role as a paramedic in primary care. For each of the following activities, please order the regularity that you undertake these in your role in primary care					
	Not at all	Sometimes	Regularly	Very regularly	All the time
Abdominal examination – including inspection, auscultation, percussion & palpation					
Assessment for lymphadenopathy					
Blood pressure					
Blood sugar					
Blood ketones					
Blood tests – FBC, ESR, iron studies, TFT, HbA1c, LFT, U&Es, haematinics, PSA, ACR, B12, drug levels, calcium, CRP, clotting factors, vitamin D, rheumatoid factor, anti CCP, urate, D-dimer, INR, TnT, autoimmune antibodies, TFT, lipid profile, testosterone, SHBG, free androgen index, FSH/LH +/- prolactin, CA125, CA19-9, CRP, coeliac screen, amylase, hepatitis, HIV, glandular fever screen/monospot, BNP/NT-proBNP					
Cardiovascular examination – including inspection, auscultation & palpation, Jugular venous pressure					
Core paramedic skills: Emergency procedures for seeking assistance and calling ambulance; provide basic life support (CPR, defibrillator); administration of nebulised therapies; management of anaphylaxis; management of suspected meningitis; management of seizures; management of					

SURVEY QUESTIONNAIRE

suspected MI; administration of oxygen					
Digital rectal examination					
Interpretation of injection fraction from echocardiogram (Echo)					
Electrocardiograph (ECG)					
Eye examination including inspection and visual acuity, fundoscopy and pupils, fluorescein, local anaesthetic					
Respiratory clinic - FeNO testing Spirometry					
Request for Imaging (eg. X-ray, CT, MRI, Ultrasound of organs, MSK and soft tissue, pelvic transvaginal and testicular)					
Interpretation for imaging					
Joint Injections					
Prescribing					
Mental health examination – Person Health Questionnaire (PHQ9); Generalised Anxiety Disorder Questionnaire (GAD7); Edinburgh Post					
Mid-stream urine culture					
Minor Surgery					
Mini mental state examination (MMSE), GPCOG and 6CIT, 480					
Musculoskeletal examination – including spine, neck, shoulders, elbows, wrists, hands, fingers, hips, pelvis, knee, ankle, feet and toes using Look/Feel/Move principles					
Neurological examination – including inspection, palpation, reflexes, sensation, power, tone, strength, pupils and nystagmus, cranial nerve and cerebellar testing					
Otoscopy					
Oxygen saturations					
Peak Expiratory Flow Rate					
Phlebotomy					

SURVEY QUESTIONNAIRE

Pulse rate, rhythm, volume and character					
Referral to specialism or specialist services					
Respiratory examination – including inspection, auscultation, percussion and palpation					
Respiratory rate					
Skin and/or nail scrapings/samples					
Smear					
Spirometry					
Sputum sample					
Stool sample – culture and sensitivity, faecal calprotectin, helicobacter-pylori testing, FIT testing or FOB					
Swabs					
Temperature					
Throat examination					
Urinalysis and HCG					
Vaginal examination (PV)					
Are there any clinical investigations and procedural skills that you undertake that we have not listed here?			Let me type (answer box)		

SURVEY QUESTIONNAIRE

**Section 5.** The purpose of this section is to understand your perspectives of your role working in primary care. You are welcome to write your answers, or bullet point them – whichever works best for you	
Why did you choose to work in primary care?	
Compared to your previous role, how do you find your current role in primary care?	Prompt: What are the challenges? What are the differences?
Do you experience conflict in your role?	Yes – go to next Q<br>No – skip
What type of conflict occurs?	
Do you receive clinical supervision in your role?	Yes – go to next Q<br>No – skip
To what extent does the clinical supervision/support you receive meet your needs?	
Do you feel patients are satisfied following a consultation with you?	Yes – go to next Q<br>No - go to next Q (negative)
Why do you feel patients are (not) satisfied following a consultation with you?	
How does Health Education England's Roadmap to Practice in primary care impact you in your role?	
What frustrations do you have about your role?	
How does your role as a paramedic in primary care make a difference to:<br>- Patients<br>- Other members of the primary care team<br>- The profession	
