# Supplementary material for: Understanding the roles and work of paramedics in primary care: a national cross-sectional survey
Source: BMJ Open. 2022 Dec 19;12(12):e067476. doi: 10.1136/bmjopen-2022-067476 (PMC9764645; doi:10.1136/bmjopen-2022-067476)
Supplement: Supplementary data [file bmjopen-2022-067476supp002.pdf]

Supplemental Table A. Correlations between clinical presentations and demogaphics of paramedics

				Clinical Supervision	Hours worked	Job Title	Length of time registered as a paramedic	Length of time in primary care	Level of Education	Prescribing Status
Spearman's rho	Cardiovascular	Blood pressure issues	Correlation Coefficient	.118	.139	.062	.002	.001	.105	.068
			Sig. (2-tailed)	0.029	0.01	0.256	0.972	0.988	0.052	0.21
			N	341	341	341	341	341	341	341
		Chest discomfort	Correlation Coefficient	.097	.065	0.212**	.024	.120	.061	.084
			Sig. (2-tailed)	0.073	0.234	0.0000	0.66	0.027	0.262	0.123
			N	341	341	341	341	341	341	341
		Chest pain	Correlation Coefficient	.016	.018	0.206**	-.002	.103	.076	.050
			Sig. (2-tailed)	0.762	0.737	0.0001	0.972	0.058	0.163	0.358
			N	341	341	341	341	341	341	341
		Irregular pulse	Correlation Coefficient	.087	.176	.036	.061	.088	.047	.057
			Sig. (2-tailed)	0.108	0.001	0.506	0.259	0.105	0.389	0.298
			N	341	341	341	341	341	341	341
		Oedema	Correlation Coefficient	.188	.130*	-.018	-.045	-.037	-.024	.010
			Sig. (2-tailed)	0.0001	0.016	0.742	0.408	0.499	0.659	0.851
			N	341	341	341	341	341	341	341
		Othopnoea	Correlation Coefficient	.058	.106	.096	.030	.053	-.029	.051
			Sig. (2-tailed)	0.283	0.051	0.076	0.584	0.325	0.588	0.349
			N	341	341	341	341	341	341	341
		Palpitations	Correlation Coefficient	.076	.094	.186**	.015	.093	.129	.171
			Sig. (2-tailed)	0.159	0.083	0.0005	0.786	0.086	0.017	0.002
			N	341	341	341	341	341	341	341
		Shortness of breath on exertion	Correlation Coefficient	.140	.177	.028	-.049	.026	.029	.059
			Sig. (2-tailed)	0.01	0.001	0.606	0.37	0.628	0.591	0.278
			N	341	341	341	341	341	341	341
	** Correlation is significant at the 0.0008 level (2-tailed).									
	Dermatology	Changes in pigmentation	Correlation Coefficient	-.011	.108	.093	.073	.100	.093	.279**
			Sig. (2-tailed)	0.843	0.045	0.087	0.177	0.066	0.087	0.000
			N	341	341	341	341	341	341	341
		Infestation	Correlation Coefficient	.022	.094	.105	.103	.206**	.159**	.365**
			Sig. (2-tailed)	0.688	0.084	0.053	0.058	0.000	0.003	0.000
			N	341	341	341	341	341	341	341
		Itching	Correlation Coefficient	.086	.037	.213**	.094	0.125	.203**	.303**
			Sig. (2-tailed)	0.111	0.500	0.000	0.084	0.021	0.000	0.000
			N	341	341	341	341	341	341	341
		Nail issues	Correlation Coefficient	.019	.093	.098	.108	.165**	.133*	.308**
			Sig. (2-tailed)	0.725	0.087	0.070	0.046	0.002	0.014	0.000
			N	341	341	341	341	341	341	341
		Rash	Correlation Coefficient	.108	-.003	.226**	.048	.063	.192**	.260**
			Sig. (2-tailed)	0.045	0.962	0.000	0.379	0.249	0.000	0.000
			N	341	341	341	341	341	341	341
		Skin lesions/moles	Correlation Coefficient	.113	.044	.182**	.036	.111	.156**	.327**
			Sig. (2-tailed)	0.038	0.420	0.001	0.503	0.041	0.004	0.000
			N	341	341	341	341	341	341	341
		Spots	Correlation Coefficient	.092	.024	.161**	.077	.164**	.188**	.313**
			Sig. (2-tailed)	0.088	0.662	0.003	0.156	0.002	0.000	0.000
			N	341	341	341	341	341	341	341
	** Correlation is significant at the 0.001 level (2-tailed).									
	Ear, Nose and Throat	Acute loss of vision	Correlation Coefficient	.042	.166	.088	-.004	.249**	.101	.176
			Sig. (2-tailed)	0.443	0.002	0.104	0.948	0.0000	0.063	0.001
			N	341	341	341	341	341	341	341
		Dizziness	Correlation Coefficient	.130	.167	.153	-.049	.089	.131	.147
			Sig. (2-tailed)	0.016	0.002	0.005	0.370	0.100	0.015	0.007
			N	341	341	341	341	341	341	341
		Eye discharge	Correlation Coefficient	.093	.047	.156**	.067	.197**	.151	.215**
			Sig. (2-tailed)	0.088	0.382	0.004	0.216	0.0002	0.005	0.0001
			N	341	341	341	341	341	341	341
		Eye injury	Correlation Coefficient	-.053	.068	.108*	.020	.140	.013	.046
			Sig. (2-tailed)	0.327	0.209	0.046	0.711	0.010	0.816	0.395
			N	341	341	341	341	341	341	341
		Foreign body	Correlation Coefficient	-.025	.020	.068	.021	.143	.024	.063
			Sig. (2-tailed)	0.645	0.719	0.208	0.706	0.008	0.664	0.245
			N	341	341	341	341	341	341	341
		Hearing loss	Correlation Coefficient	.084	.192	.071	.011	.149**	.091	.308**
			Sig. (2-tailed)	0.122	0.0004	0.188	0.834	0.006	0.095	0.0000
			N	341	341	341	341	341	341	341
		Mouth pain	Correlation Coefficient	.043	.080	.014	-.036	.092	.066	.181
			Sig. (2-tailed)	0.431	0.140	0.797	0.507	0.089	0.222	0.001
			N	341	341	341	341	341	341	341
		Nasal obstruction	Correlation Coefficient	-.060	.159	.093	.012	.212**	.134	.267**
			Sig. (2-tailed)	0.272	0.003	0.088	0.821	0.0001	0.013	0.0000
			N	341	341	341	341	341	341	341
		Nasal pain	Correlation Coefficient	.071	.143	.124	.030	.149	.160	.324**
			Sig. (2-tailed)	0.194	0.008	0.022	0.584	0.006	0.003	0.0000
			N	341	341	341	341	341	341	341
		Neck swelling	Correlation Coefficient	.057	.098	.139	.004	.119	.198**	.225**
			Sig. (2-tailed)	0.293	0.072	0.010	0.948	0.028	0.0002	0.0000
			N	341	341	341	341	341	341	341
		Otalgia	Correlation Coefficient	.104	.009	.243**	.099	.121	.128	.214**
			Sig. (2-tailed)	0.056	0.865	0.0000	0.069	0.026	0.018	0.0001
			N	341	341	341	341	341	341	341
			Correlation Coefficient	.120	.056	.210**	.069	.171	.134	.259**

Eyes, E	Otorrhoea	Sig. (2-tailed)	0.027	0.305	0.0001	0.206	0.002	0.013	0.0000
		N	341	341	341	341	341	341	341
	Red eye	Correlation Coefficient	.098	.069	.141	-.004	.153	.130	.189
		Sig. (2-tailed)	0.070	0.201	0.009	0.943	0.005	0.016	0.0005
		N	341	341	341	341	341	341	341
	Sinus pain	Correlation Coefficient	.107	.137	.202**	.075	.164	.200**	.307**
		Sig. (2-tailed)	0.048	0.011	0.0002	0.167	0.002	0.0002	0.0000
		N	341	341	341	341	341	341	341
	Sore throat	Correlation Coefficient	.078	.072	.210**	.043	.083	.171	.134
		Sig. (2-tailed)	0.148	0.183	0.0001	0.430	0.127	0.002	0.013
		N	341	341	341	341	341	341	341
	Swollen eyelid	Correlation Coefficient	.090	.099	.146	.056	.146	.151	.259**
		Sig. (2-tailed)	0.098	0.069	0.007	0.303	0.007	0.005	0.0000
		N	341	341	341	341	341	341	341
	Throat swelling	Correlation Coefficient	.003	.101	.140	-.096	.132	.161	.192
		Sig. (2-tailed)	0.955	0.063	0.010	0.079	0.015	0.003	0.0004
		N	341	341	341	341	341	341	341
	Tinnitus	Correlation Coefficient	.097	.066	.119	.040	.101	.195**	.215**
		Sig. (2-tailed)	0.074	0.224	0.029	0.459	0.062	0.0003	0.0001
		N	341	341	341	341	341	341	341
	Vertigo	Correlation Coefficient	.111	.121	.116	-.004	.078	.181	.176
		Sig. (2-tailed)	0.040	0.025	0.032	0.937	0.151	0.001	0.001
		N	341	341	341	341	341	341	341
	Visual disturbance	Correlation Coefficient	.085	.111	.103	.005	.168	.106	.191
		Sig. (2-tailed)	0.119	0.042	0.058	0.934	0.002	0.051	0.0004
		N	341	341	341	341	341	341	341
	Voice changes	Correlation Coefficient	.034	.099	.047	.015	.216**	.090	.274**
		Sig. (2-tailed)	0.535	0.067	0.389	0.777	0.0001	0.097	0.0000
		N	341	341	341	341	341	341	341

** Correlation is significant at the 0.0003 level (2-tailed).

Female and Male Anatamoical Health	Acute groin swelling/pain	Correlation Coefficient	.038	.140	.202**	-.017	.184**	.206**	.276**
		Sig. (2-tailed)	0.487	0.010	0.0002	0.754	0.001	0.0001	0.00000
		N	341	341	341	341	341	341	341
	Breast symptoms	Correlation Coefficient	-.024	-.038	.138	-.018	.087	.169	.191**
		Sig. (2-tailed)	0.664	0.483	0.011	0.735	0.109	0.002	0.0004
		N	341	341	341	341	341	341	341
	Genital rashes/irritation	Correlation Coefficient	-.024	.094	.239**	.022	.145	.188**	.332**
		Sig. (2-tailed)	0.662	0.082	0.00001	0.688	0.007	0.0005	0.00000
		N	341	341	341	341	341	341	341
	Pelvic pain/mass	Correlation Coefficient	-.024	.136	.167	.050	.164	.169	.242**
		Sig. (2-tailed)	0.665	0.012	0.002	0.361	0.002	0.002	0.00001
		N	341	341	341	341	341	341	341
	Penile discharge	Correlation Coefficient	.032	.133	.137	.037	.158	.195**	.293**
		Sig. (2-tailed)	0.560	0.014	0.011	0.499	0.003	0.0003	0.00000
		N	341	341	341	341	341	341	341
	Penile pain	Correlation Coefficient	.039	.147	.200**	-.027	.170	.234**	.331**
		Sig. (2-tailed)	0.469	0.007	0.0002	0.614	0.002	0.00001	0.00000
		N	341	341	341	341	341	341	341
	Urinary symptoms	Correlation Coefficient	.042	.036	.174**	-.003	.013	.060	.134
		Sig. (2-tailed)	0.439	0.513	0.001	0.949	0.804	0.273	0.013
		N	341	341	341	341	341	341	341

** Correlation is significant at the 0.001 level (2-tailed).

Gastrointestinal & Hepatic System	Abdominal blood results	Correlation Coefficient	.187	.131	.117	.033	.065	.142	.224**
		Sig. (2-tailed)	0.001	0.015	0.031	0.538	0.229	0.009	0.0000
		N	341	341	341	341	341	341	341
	Abdominal distention	Correlation Coefficient	.041	.249**	.042	-.071	.085	.018	.012
		Sig. (2-tailed)	0.455	0.0000	0.440	0.188	0.116	0.737	0.820
		N	341	341	341	341	341	341	341
	Abdominal mass/swelling	Correlation Coefficient	.038	.207**	.023	-.041	.096	.009	.059
		Sig. (2-tailed)	0.489	0.0001	0.671	0.451	0.076	0.872	0.274
		N	341	341	341	341	341	341	341
	Abdominal pain	Correlation Coefficient	.060	.023	.133	.001	.045	.113	.093
		Sig. (2-tailed)	0.273	0.678	0.014	0.991	0.410	0.037	0.085
		N	341	341	341	341	341	341	341
	Change in bowel habit	Correlation Coefficient	.064	.004	.084	-.041	.002	.091	.144
		Sig. (2-tailed)	0.238	0.943	0.120	0.451	0.973	0.094	0.008
		N	341	341	341	341	341	341	341
	Constipation	Correlation Coefficient	.102	.134	.072	-.014	.013	.041	.115
		Sig. (2-tailed)	0.060	0.013	0.187	0.791	0.813	0.448	0.034
		N	341	341	341	341	341	341	341
	Diarrhoea	Correlation Coefficient	.042	.094	.064	-.039	.052	.041	.116
		Sig. (2-tailed)	0.434	0.082	0.239	0.477	0.338	0.451	0.032
		N	341	341	341	341	341	341	341
	Difficulty swallowing	Correlation Coefficient	.070	.152	.079	-.007	.145	.026	.184
		Sig. (2-tailed)	0.195	0.005	0.144	0.902	0.007	0.627	0.001
		N	341	341	341	341	341	341	341
	Excessive thirst	Correlation Coefficient	.014	.183	.076	-.056	.036	.077	.174
		Sig. (2-tailed)	0.797	0.001	0.161	0.300	0.507	0.158	0.001
		N	341	341	341	341	341	341	341
	Hematemesis	Correlation Coefficient	-.012	.103	.098	-.019	.143	.116	.133
		Sig. (2-tailed)	0.820	0.058	0.070	0.732	0.008	0.032	0.014
		N	341	341	341	341	341	341	341
	High risk behaviours and	Correlation Coefficient	-.017	.143	.016	.121	.138	-.011	.086
		Sig. (2-tailed)	0.750	0.008	0.768	0.025	0.011	0.836	0.114

	concerns	N	341	341	341	341	341	341	341
	Indigestion	Correlation Coefficient	.142	.097	.222**	.008	.138	.157	.262**
		Sig. (2-tailed)	0.009	0.073	0.0000	0.883	0.011	0.004	0.0000
		N	341	341	341	341	341	341	341
	Nausea/vomiting	Correlation Coefficient	.138	.102	.123	.005	.055	.078	.159
		Sig. (2-tailed)	0.011	0.060	0.023	0.929	0.308	0.152	0.003
		N	341	341	341	341	341	341	341
	Poor appetite	Correlation Coefficient	.090	.098	.034	.043	.045	-.001	.070
		Sig. (2-tailed)	0.095	0.070	0.530	0.430	0.407	0.978	0.197
		N	341	341	341	341	341	341	341
	Rectal bleeding	Correlation Coefficient	.074	.128	.153	.012	.117	.119	.242**
		Sig. (2-tailed)	0.174	0.018	0.005	0.820	0.031	0.028	0.0000
		N	341	341	341	341	341	341	341
	Stoma issues	Correlation Coefficient	-.033	.242**	-.017	.047	.107	-.065	.130
		Sig. (2-tailed)	0.544	0.0000	0.754	0.383	0.049	0.235	0.016
		N	341	341	341	341	341	341	341

** Correlation is significant at the 0.0004 level (2-tailed).

Key Presentations	Fever	Correlation Coefficient	.038	.070	.150	.000	.044	.101	.127
		Sig. (2-tailed)	0.486	0.200	0.006	0.999	0.415	0.063	0.019
		N	341	341	341	341	341	341	341
	Generalised aches and pains	Correlation Coefficient	.121	.052	.069	-.037	-.001	.064	.081
		Sig. (2-tailed)	0.026	0.335	0.203	0.493	0.988	0.238	0.134
		N	341	341	341	341	341	341	341
	Genetic predisposition	Correlation Coefficient	.029	.029	.036	.049	.069	.043	.027
		Sig. (2-tailed)	0.593	0.590	0.504	0.365	0.204	0.424	0.617
		N	341	341	341	341	341	341	341
	Lymphadenopathy	Correlation Coefficient	.138	.136	.125	0.054	0.057	.149	.228**
		Sig. (2-tailed)	0.011	0.012	0.021	0.318	0.298	0.006	0.00002
		N	341	341	341	341	341	341	341
	Overdose/ poisoning	Correlation Coefficient	-.048	.254**	.101	-.073	.160	.057	.074
		Sig. (2-tailed)	0.377	0.00000	0.061	0.181	0.003	0.290	0.174
		N	341	341	341	341	341	341	341
	Presentations in patients with a learning disability	Correlation Coefficient	.024	.178	.075	.097	.117	.029	.063
		Sig. (2-tailed)	0.653	0.001	0.166	0.075	0.031	0.588	0.249
		N	341	341	341	341	341	341	341
	Review of blood test results	Correlation Coefficient	.144	.094	.201	-.001	.087	.199	.282**
		Sig. (2-tailed)	0.008	0.082	0.0002	0.982	0.109	0.0002	0.00000
		N	341	341	341	341	341	341	341
	Sleep issues	Correlation Coefficient	.074	.060	.040	-.104	-.039	.080	.107
		Sig. (2-tailed)	0.171	0.267	0.457	0.056	0.472	0.140	0.048
		N	341	341	341	341	341	341	341
	Substance/alcohol misuse	Correlation Coefficient	.014	.127	.115	-.018	.175	.092	.092
		Sig. (2-tailed)	0.791	0.019	0.034	0.743	0.001	0.091	0.090
		N	341	341	341	341	341	341	341
	Tired all the time	Correlation Coefficient	.165	.077	.096	-.036	.029	.105	.160
		Sig. (2-tailed)	0.002	0.154	0.077	0.513	0.590	0.053	0.003
		N	341	341	341	341	341	341	341
	Vulnerable adult, family/carer concern	Correlation Coefficient	.072	.216	-.033	.016	.054	-.069	-.013
		Sig. (2-tailed)	0.185	0.0001	0.545	0.765	0.320	0.205	0.807
		N	341	341	341	341	341	341	341

** Correlation is significant at the 0.0004 level (2-tailed).

Medication Review and Issues	Abnormal blood test results	Correlation Coefficient	.105	.098	.194**	.055	.095	.186	.264**
		Sig. (2-tailed)	0.052	0.072	0.0003	0.312	0.080	0.001	0.00000
		N	341	341	341	341	341	341	341
	Adverse side effects of medication	Correlation Coefficient	.026	.080	.118	.046	.222**	.131	.255**
		Sig. (2-tailed)	0.626	0.142	0.029	0.402	0.00003	0.016	0.00000
		N	341	341	341	341	341	341	341
	Higher risk groups (risk reduction medications)	Correlation Coefficient	.044	.116	.159	.078	.206**	.145	.235**
		Sig. (2-tailed)	0.416	0.033	0.003	0.152	0.0001	0.007	0.00001
		N	341	341	341	341	341	341	341
	Ineffective medication	Correlation Coefficient	.015	.085	.145	.044	.149	.139	.274**
		Sig. (2-tailed)	0.776	0.117	0.007	0.420	0.006	0.010	0.00000
		N	341	341	341	341	341	341	341
	Issues with polypharmacy	Correlation Coefficient	.038	.145	.187	.120	.195**	.163	.302**
		Sig. (2-tailed)	0.488	0.007	0.001	0.026	0.0003	0.002	0.00000
		N	341	341	341	341	341	341	341
	Misuse of medication	Correlation Coefficient	-.005	.130	.156	.101	.188**	.128	.203
		Sig. (2-tailed)	0.929	0.017	0.004	0.063	0.0005	0.018	0.0002
		N	341	341	341	341	341	341	341
	Overuse of medication	Correlation Coefficient	.041	.083	.230**	.099	.197**	.122	.243**
		Sig. (2-tailed)	0.451	0.127	0.00002	0.068	0.0002	0.024	0.00001
		N	341	341	341	341	341	341	341
	Poor compliance with medication	Correlation Coefficient	.071	.105	.135	.103	.144	.110	.191**
		Sig. (2-tailed)	0.188	0.052	0.012	0.058	0.008	0.042	0.0004
		N	341	341	341	341	341	341	341

** Correlation is significant at the 0.0008 level (2-tailed).

	Difficulty with movement	Correlation Coefficient	.054	.104	.113	.036	.023	.023	.072
		Sig. (2-tailed)	0.317	0.055	0.037	0.508	0.669	0.668	0.186
		N	341	341	341	341	341	341	341
	Minor injury	Correlation Coefficient	-.007	.082	.061	-.049	.040	-.020	-.023
		Sig. (2-tailed)	0.902	0.129	0.265	0.369	0.467	0.709	0.669
		N	341	341	341	341	341	341	341

Musculoskeletal	Pain	Correlation Coefficient	.057	.051	.153	.070	.019	.063	.039
		Sig. (2-tailed)	0.296	0.346	0.005	0.198	0.725	0.249	0.472
		N	341	341	341	341	341	341	341
	Redness	Correlation Coefficient	.051	.164	.146	.031	.092	.006	.093
		Sig. (2-tailed)	0.350	0.002	0.007	0.569	0.090	0.907	0.086
		N	341	341	341	341	341	341	341
	Stiffness	Correlation Coefficient	.064	.100	.128	.040	.062	.042	.112
		Sig. (2-tailed)	0.240	0.064	0.018	0.456	0.253	0.442	0.039
		N	341	341	341	341	341	341	341
	Swelling	Correlation Coefficient	.033	.133	.152	.027	.093	.023	.077
		Sig. (2-tailed)	0.540	0.014	0.005	0.617	0.087	0.679	0.155
		N	341	341	341	341	341	341	341
** Correlation is significant at the 0.001 level (2-tailed).									
Neurological	Altered gait	Correlation Coefficient	.062	.165	.018	.095	.075	.019	.053
		Sig. (2-tailed)	0.251	0.002	0.743	0.081	0.169	0.729	0.326
		N	341	341	341	341	341	341	341
	Altered level of consciousness	Correlation Coefficient	.024	.170	.054	.081	.156	-.012	.036
		Sig. (2-tailed)	0.656	0.002	0.320	0.136	0.004	0.820	0.508
		N	341	341	341	341	341	341	341
	Altered level of consciousness	Correlation Coefficient	.013	.176	.039	.068	.149	-.022	.064
		Sig. (2-tailed)	0.812	0.001	0.472	0.209	0.006	0.687	0.239
		N	341	341	341	341	341	341	341
	Altered power, tone or sensitivity	Correlation Coefficient	.039	.120*	.119	.044	.101	.096	.065
		Sig. (2-tailed)	0.473	0.027	0.028	0.423	0.062	0.076	0.231
		N	341	341	341	341	341	341	341
	Confusion	Correlation Coefficient	.071	.229**	.012	.070	.116	.017	.128
		Sig. (2-tailed)	0.190	0.00002	0.827	0.196	0.032	0.753	0.018
		N	341	341	341	341	341	341	341
	Dizziness	Correlation Coefficient	.101	.061	.125	.004	.042	.144	.092
		Sig. (2-tailed)	0.061	0.258	0.021	0.937	0.436	0.008	0.090
		N	341	341	341	341	341	341	341
	Facial palsy	Correlation Coefficient	.017	.131	.080	.086	.185	.051	.184
		Sig. (2-tailed)	0.754	0.015	0.138	0.112	0.001	0.347	0.001
		N	341	341	341	341	341	341	341
	Fits faints and funny turns	Correlation Coefficient	.096	.110	.118	-.016	.118	.069	.060
		Sig. (2-tailed)	0.078	0.043	0.030	0.762	0.029	0.204	0.268
		N	341	341	341	341	341	341	341
	Head injury	Correlation Coefficient	-.028	.029	.092	-.053	.068	.037	.001
		Sig. (2-tailed)	0.605	0.593	0.088	0.330	0.207	0.498	0.989
		N	341	341	341	341	341	341	341
	Headache	Correlation Coefficient	.103	.046	.195	-.046	.025	.172	.153
		Sig. (2-tailed)	0.057	0.396	0.0003	0.396	0.643	0.001	0.005
		N	341	341	341	341	341	341	341
	Memory problems	Correlation Coefficient	.090	.178	-.028	.075	.084	.032	.130
		Sig. (2-tailed)	0.098	0.001	0.607	0.166	0.120	0.553	0.016
		N	341	341	341	341	341	341	341
	Paraesthesia	Correlation Coefficient	.017	.081	.189	.057	.211	.139	.199
		Sig. (2-tailed)	0.758	0.138	0.0005	0.298	0.0001	0.010	0.0002
		N	341	341	341	341	341	341	341
	Speech changes	Correlation Coefficient	.031	.224**	.011	.049	.184	.014	.097
		Sig. (2-tailed)	0.567	0.00003	0.841	0.367	0.001	0.797	0.073
		N	341	341	341	341	341	341	341
	Tremor	Correlation Coefficient	.039	.127	.031	.013	.134	.042	.099
		Sig. (2-tailed)	0.474	0.019	0.568	0.813	0.014	0.440	0.067
		N	341	341	341	341	341	341	341
	Weakness	Correlation Coefficient	.104	.133	.068	.025	.050	.032	.030
		Sig. (2-tailed)	0.054	0.014	0.210	0.650	0.361	0.550	0.581
		N	341	341	341	341	341	341	341
** Correlation is significant at the 0.0004 level (2-tailed).									
	Abdominal pain	Correlation Coefficient	341	341	341	341	341	341	341
		Sig. (2-tailed)	0.334	0.977	0.00000	0.811	0.002	0.00000	0.00000
		N	.053	-.002	.293**	.013	.171	.249**	.260**
	Acute bowel symptoms	Correlation Coefficient	.040	.014	.254**	-.058	.144	.217**	.234**
		Sig. (2-tailed)	0.461	0.800	0.00000	0.285	0.008	0.00005	0.00001
		N	341	341	341	341	341	341	341
	Constipation	Correlation Coefficient	.049	.052	.266**	.001	.134	.248**	.294**
		Sig. (2-tailed)	0.371	0.335	0.00000	0.987	0.013	0.00000	0.00000
		N	341	341	341	341	341	341	341
	Cough/wheeze/stridor/respiratory distress/nasal symptoms	Correlation Coefficient	.043	-.032	.249**	.005	.109	.203	.148
		Sig. (2-tailed)	0.433	0.557	0.00000	0.923	0.045	0.0002	0.006
		N	341	341	341	341	341	341	341
	Crying baby	Correlation Coefficient	-.025	-.048	.296**	-.069	.149	.214	.192
		Sig. (2-tailed)	0.639	0.372	0.00000	0.201	0.006	0.0001	0.0004
		N	341	341	341	341	341	341	341
	Diarrhoea	Correlation Coefficient	.059	.009	.246**	-.046	.149	.237**	.230**
		Sig. (2-tailed)	0.280	0.866	0.00000	0.393	0.006	0.00001	0.00002
		N	341	341	341	341	341	341	341
	Eye injury	Correlation Coefficient	.003	.061	.116	-.042	.197	.117	.146
		Sig. (2-tailed)	0.961	0.260	0.032	0.434	0.0002	0.030	0.007
		N	341	341	341	341	341	341	341

Paediatrics	Minor injury	Correlation Coefficient	-.074	-.030	.155	-.039	.190	.081	.092
		Sig. (2-tailed)	0.174	0.583	0.004	0.472	0.0004	0.137	0.090
		N	341	341	341	341	341	341	341
	Musculoskeletal symptoms	Correlation Coefficient	-.031	-.024	.247**	.015	.173	.158	.167
		Sig. (2-tailed)	0.572	0.660	0.00000	0.778	0.001	0.003	0.002
		N	341	341	341	341	341	341	341
	Otalgia/otorrhoea	Correlation Coefficient	.070	-.024	.273**	-.010	.139*	.190	.223**
		Sig. (2-tailed)	0.196	0.663	0.00000	0.851	0.010	0.0004	0.00003
		N	341	341	341	341	341	341	341
	Pyrexia of unknown origin	Correlation Coefficient	.004	-.063	.265**	-.048	.090	.225**	.145
		Sig. (2-tailed)	0.947	0.248	0.00000	0.372	0.097	0.00003	0.007
		N	341	341	341	341	341	341	341
	Rashes	Correlation Coefficient	.023	-.039	.261**	-.007	.142	.208	.238**
		Sig. (2-tailed)	0.671	0.472	0.00000	0.904	0.009	0.0001	0.00001
		N	341	341	341	341	341	341	341
	Red eye/discharge	Correlation Coefficient	.045	.035	.222**	.026	.212	.193	.253**
		Sig. (2-tailed)	0.405	0.517	0.00003	0.628	0.0001	0.0003	0.00000
		N	341	341	341	341	341	341	341
	Sore throat	Correlation Coefficient	.053	-.020	.270**	.003	.099	.224**	.185
		Sig. (2-tailed)	0.327	0.713	0.00000	0.962	0.067	0.00003	0.001
		N	341	341	341	341	341	341	341
	Vomiting	Correlation Coefficient	.066	.008	.262**	-.053	.149	.224**	.225**
		Sig. (2-tailed)	0.227	0.885	0.00000	0.333	0.006	0.00003	0.00003
		N	341	341	341	341	341	341	341
	Vulnerable child	Correlation Coefficient	-.059	.103	.200**	.022	.175	.159	.196**
		Sig. (2-tailed)	0.279	0.057	0.0002	0.686	0.001	0.003	0.0003
		N	341	341	341	341	341	341	341

** Correlation is significant at the 0.0004 level (2-tailed).

Pain	Acute pain	Correlation Coefficient	.100	.071	.160	.053	.096	.127	.122
		Sig. (2-tailed)	0.066	0.190	0.003	0.326	0.076	0.019	0.024
		N	341	341	341	341	341	341	341
	Change in type of pain	Correlation Coefficient	.028	.079	.121	.048	.079	.017	.133
		Sig. (2-tailed)	0.606	0.145	0.026	0.373	0.148	0.750	0.014
		N	341	341	341	341	341	341	341
	Worsening pain	Correlation Coefficient	.052	.079	.140	.046	.035	.050	.090
		Sig. (2-tailed)	0.337	0.144	0.010	0.398	0.523	0.359	0.097
		N	341	341	341	341	341	341	341

** Correlation is significant at the 0.002 level (2-tailed).

Palliative and End of Life Care	Discussions about advanced care planning	Correlation Coefficient	.047	.205**	-.093	.051	.168	-.043	.071
		Sig. (2-tailed)	0.382	0.0001	0.088	0.349	0.002	0.433	0.190
		N	341	341	341	341	341	341	341
	Symptom management	Correlation Coefficient	.056	.205**	-.046	.051	.089	-.029	.044
		Sig. (2-tailed)	0.300	0.0001	0.399	0.349	0.103	0.593	0.418
		N	341	341	341	341	341	341	341

** Correlation is significant at the 0.003 level (2-tailed).

Renal and Genitourinary	Acute groin swelling/pain	Correlation Coefficient	.017	.155	.175	.076	.174	.141	.259**
		Sig. (2-tailed)	0.750	0.004	0.001	0.161	0.001	0.009	0.0000
		N	341	341	341	341	341	341	341
	Groin pain	Correlation Coefficient	.104	.135	.184	-.064	.110	.126	.260**
		Sig. (2-tailed)	0.055	0.012	0.001	0.238	0.041	0.020	0.0000
		N	341	341	341	341	341	341	341
	Haematuria	Correlation Coefficient	.132	.134	.156	.075	.116	.111	.226**
		Sig. (2-tailed)	0.015	0.014	0.004	0.165	0.032	0.041	0.0000
		N	341	341	341	341	341	341	341
	Inability to pass urine	Correlation Coefficient	.048	.206**	.046	.039	.131	.064	.101
		Sig. (2-tailed)	0.378	0.0001	0.400	0.471	0.016	0.240	0.063
		N	341	341	341	341	341	341	341
	Kidney disease	Correlation Coefficient	.090	.209**	.127	.028	.074	.119	.204**
		Sig. (2-tailed)	0.099	0.0001	0.019	0.601	0.173	0.028	0.0001
		N	341	341	341	341	341	341	341
	Loin pain	Correlation Coefficient	.093	.109	.189**	.027	.143	.143	.266**
		Sig. (2-tailed)	0.086	0.044	0.0004	0.622	0.008	0.008	0.0000
		N	341	341	341	341	341	341	341
	Penile pain	Correlation Coefficient	.037	.151	.095	.003	.162	.097	.271**
		Sig. (2-tailed)	0.495	0.005	0.079	0.949	0.003	0.073	0.0000
		N	341	341	341	341	341	341	341
	Profuse vaginal bleeding	Correlation Coefficient	-.044	-.006	.153	.067	.157	.117	.130
		Sig. (2-tailed)	0.416	0.917	0.005	0.218	0.004	0.031	0.017
		N	341	341	341	341	341	341	341
	Recurrent infection	Correlation Coefficient	.100	.141	.055	.058	-.016	.053	.081
		Sig. (2-tailed)	0.065	0.009	0.313	0.283	0.762	0.332	0.134
		N	341	341	341	341	341	341	341
	Testicular pain/swelling	Correlation Coefficient	.017	.129	.211**	.035	.215**	.182	.351**
		Sig. (2-tailed)	0.755	0.017	0.0001	0.517	0.0001	0.001	0.0000
		N	341	341	341	341	341	341	341
	Urinary symptoms	Correlation Coefficient	.136	.058	.089	.022	-.073	.060	.097
		Sig. (2-tailed)	0.012	0.282	0.102	0.691	0.181	0.266	0.074
		N	341	341	341	341	341	341	341

** Correlation is significant at the 0.0006 level (2-tailed).

	Acute Covid-19	Correlation Coefficient	.005	.121	.125	-.079	.029	.063	.069
		Sig. (2-tailed)	0.931	0.026	0.021	0.148	0.588	0.248	0.202
		N	341	341	341	341	341	341	341
	Breathing	Correlation Coefficient	.069	.111	.114	.023	.105	.097	.129

	Breathing difficulties	Sig. (2-tailed)	0.202	0.041	0.036	0.678	0.052	0.074	0.017
		N	341	341	341	341	341	341	341
	Cough (including haemoptysis)	Correlation Coefficient	.090	.129	.133	-.005	.054	.126	.140
		Sig. (2-tailed)	0.097	0.017	0.014	0.934	0.321	0.020	0.010
		N	341	341	341	341	341	341	341
	Cyanosis	Correlation Coefficient	-.054	.143	.058	.021	.144	.068	.125
		Sig. (2-tailed)	0.317	0.008	0.284	0.693	0.008	0.213	0.021
		N	341	341	341	341	341	341	341
Respiratory	Pain on breathing	Correlation Coefficient	.107	.104	.118	-.009	.088	.107	.106
		Sig. (2-tailed)	0.047	0.055	0.030	0.872	0.105	0.048	0.051
		N	341	341	341	341	341	341	341
	Pallor	Correlation Coefficient	-.038	.174	.060	.064	.120	.026	.108
		Sig. (2-tailed)	0.482	0.001	0.272	0.242	0.026	0.638	0.046
		N	341	341	341	341	341	341	341
	Post Covid-19 syndrome	Correlation Coefficient	-.028	.090	.042	-.062	.034	.072	.093
		Sig. (2-tailed)	0.603	0.097	0.435	0.252	0.535	0.186	0.087
		N	341	341	341	341	341	341	341
	Shortness of breath	Correlation Coefficient	.064	.126	.082	-.011	.067	.076	.107
		Sig. (2-tailed)	0.241	0.020	0.130	0.833	0.218	0.162	0.048
		N	341	341	341	341	341	341	341
	Suspected or recurrent infection	Correlation Coefficient	.077	.080	.090	.035	-.015	.100	.052
		Sig. (2-tailed)	0.154	0.138	0.097	0.515	0.789	0.064	0.342
		N	341	341	341	341	341	341	341
	Wheeze	Correlation Coefficient	.086	.128	.126	.046	.100	.121	.146
		Sig. (2-tailed)	0.113	0.018	0.020	0.393	0.066	0.026	0.007
		N	341	341	341	341	341	341	341

** Correlation is significant at the 0.0007 level (2-tailed).

Supplemental Table B. Correlations between clinical examinations and demogaphics of paramedics

			Clinical Supervision	Hours worked	Job Title	Length of time in primary care	Level of Education	Prescribing Status
Spearman's rho	Abdominal examination	Correlation Coefficient	.062	.116	.129	.091	.113	.117
		Sig. (2-tailed)	0.254	0.032	0.017	0.094	0.036	0.031
		N	341	341	341	341	341	341
	Assessment for lymphadenopathy	Correlation Coefficient	.062	.019	.186	.148	.211**	.257**
		Sig. (2-tailed)	0.254	0.730	0.001	0.006	0.0001	0.0000
		N	341	341	341	341	341	341
	Blood pressure	Correlation Coefficient	.064	.094	.072	.058	.071	.017
		Sig. (2-tailed)	0.238	0.085	0.183	0.284	0.190	0.751
		N	341	341	341	341	341	341
	Cardiovascular examination	Correlation Coefficient	.081	.113	.111	.065	.087	.066
		Sig. (2-tailed)	0.138	0.037	0.040	0.230	0.109	0.226
		N	341	341	341	341	341	341
	Cranial nerve examination	Correlation Coefficient	.048	.088	.166	.139	.155	.132
		Sig. (2-tailed)	0.376	0.106	0.002	0.010	0.004	0.015
		N	341	341	341	341	341	341
	Digital rectal examination	Correlation Coefficient	.007	.141	.217**	.195	.264**	.444**
		Sig. (2-tailed)	0.902	0.009	0.0001	0.0003	0.0000	0.0000
		N	341	341	341	341	341	341
	Echocardiogram (Echo)	Correlation Coefficient	-.066	.065	.052	.082	.091	.138
		Sig. (2-tailed)	0.228	0.236	0.343	0.133	0.096	0.011
		N	339	339	339	339	339	339
	Electrocardiograph (ECG)	Correlation Coefficient	-.047	.150	.053	.127	.027	.055
		Sig. (2-tailed)	0.388	0.005	0.329	0.019	0.616	0.311
		N	341	341	341	341	341	341
	Eye examination	Correlation Coefficient	-.036	.017	.105	.204**	.212**	.255**
		Sig. (2-tailed)	0.513	0.753	0.054	0.0002	0.0001	0.000
		N	341	341	341	341	341	341
	FeNO testing	Correlation Coefficient	-.193	.015	.026	.060	.091	.121
		Sig. (2-tailed)	0.0003	0.790	0.632	0.271	0.094	0.025
		N	341	341	341	341	341	341
	Imaging	Correlation Coefficient	-.005	.084	.214**	.129	.127	.185
		Sig. (2-tailed)	0.924	0.124	0.0001	0.018	0.019	0.001
		N	341	341	341	341	341	341
	Jugular venous pressure	Correlation Coefficient	.077	.062	.089	.124	.137	.181
		Sig. (2-tailed)	0.155	0.256	0.099	0.022	0.011	0.001
		N	341	341	341	341	341	341
	Mental health examination	Correlation Coefficient	.092	.037	.073	.126	.129	.178
		Sig. (2-tailed)	0.089	0.496	0.177	0.020	0.017	0.001
		N	341	341	341	341	341	341
	Mid-stream urine culture	Correlation Coefficient	.113	.179	0.075	0.013	0.093	.129
		Sig. (2-tailed)	0.037	0.001	0.167	0.807	0.088	0.017
		N	341	341	341	341	341	341
	Mini mental state examination (MMSE)	Correlation Coefficient	.094	.126	.040	.128	.081	.084
		Sig. (2-tailed)	0.082	0.020	0.464	0.018	0.135	0.120
		N	341	341	341	341	341	341
	Musculoskeletal examinatio	Correlation Coefficient	.017	.086	.153	.133	.112	.073
		Sig. (2-tailed)	0.758	0.113	0.005	0.014	0.039	0.180
		N	340	340	340	340	340	340
	Neurological examination	Correlation Coefficient	.008	.084	.125	.067	.124	.052
		Sig. (2-tailed)	0.887	0.123	0.021	0.217	0.022	0.335
		N	341	341	341	341	341	341
	Otoscopy	Correlation Coefficient	.058	.053	.162	.088	.088	.193
		Sig. (2-tailed)	0.282	0.332	0.003	0.106	0.104	0.0003
		N	341	341	341	341	341	341
	Oxygen saturations	Correlation Coefficient	.048	.080	.029	.029	-.067	-.105
		Sig. (2-tailed)	0.372	0.142	0.593	0.599	0.217	0.052
		N	341	341	341	341	341	341
	Peak Expiratory Flow Rate	Correlation Coefficient	-.006	.045	.036	.183	.037	.123
		Sig. (2-tailed)	0.909	0.407	0.510	0.001	0.491	0.023
		N	341	341	341	341	341	341
	Phlebotomy	Correlation Coefficient	.016	.220**	-.105	-.085	-.047	.033
		Sig. (2-tailed)	0.762	0.0000	0.053	0.116	0.385	0.539

	N	341	341	341	341	341	341
Pulse rate, rhythm, volume and character	Correlation Coefficient	-.016	.057	.044	.054	-.077	-.055
	Sig. (2-tailed)	0.774	0.292	0.422	0.320	0.156	0.310
	N	341	341	341	341	341	341
Referral to specialism or specialist services	Correlation Coefficient	.086	.168	.061	.143	.118	.190
	Sig. (2-tailed)	0.111	0.002	0.258	0.008	0.029	0.0004
	N	341	341	341	341	341	341
Respiratory examination	Correlation Coefficient	-.012	.085	.087	.085	.038	.034
	Sig. (2-tailed)	0.829	0.116	0.109	0.116	0.480	0.527
	N	341	341	341	341	341	341
Respiratory rate	Correlation Coefficient	.013	.084	.024	.018	-.058	-.063
	Sig. (2-tailed)	0.813	0.121	0.666	0.742	0.285	0.248
	N	340	340	340	340	340	340
Skin and/or nail scrapings/samples	Correlation Coefficient	.045	.124	.072	.149	.042	.296**
	Sig. (2-tailed)	0.410	0.022	0.183	0.006	0.444	0.0000
	N	341	341	341	341	341	341
Spirometry	Correlation Coefficient	-.015	.004	.022	-.001	.032	.046
	Sig. (2-tailed)	0.778	0.940	0.687	0.986	0.561	0.402
	N	341	341	341	341	341	341
Sputum sample	Correlation Coefficient	.134	.209**	.084	.152	.118	.290**
	Sig. (2-tailed)	0.013	0.0001	0.121	0.005	0.030	0.0000
	N	341	341	341	341	341	341
Stool sample	Correlation Coefficient	.212**	.107	.126	.049	.142	.257**
	Sig. (2-tailed)	0.0001	0.049	.021	0.363	0.009	0.0000
	N	341	341	341	341	341	341
Swabs	Correlation Coefficient	.126	.211**	.082	.089	.090	.277**
	Sig. (2-tailed)	0.020	0.0001	0.133	0.099	0.097	0.0000
	N	341	341	341	341	341	341
Temperature	Correlation Coefficient	.023	.067	-.023	-.019	-.103	-.114
	Sig. (2-tailed)	0.676	0.215	0.672	0.733	0.057	0.035
	N	341	341	341	341	341	341
Throat examination	Correlation Coefficient	.071	.072	.153	.063	.082	.102
	Sig. (2-tailed)	0.193	0.183	0.005	0.243	0.129	0.061
	N	341	341	341	341	341	341
Urinalysis	Correlation Coefficient	.071	.104	.029	.050	-.004	.033
	Sig. (2-tailed)	0.193	0.056	0.592	0.356	0.947	0.548
	N	341	341	341	341	341	341

**. Correlation is significant at the 0.0002 level (2-tailed).

Supplemental Table C. Relationship between core capabilities of primary care and demographics of paramedics

			Clinical Supervision	Hours worked	Job Title	Length of time registered as a paramedic	Length of time in primary care	Level of Education	Prescribing Status	Salary
Spearman's rho	Communication and consultations	Correlation Coefficient	-.042	-.050	.138	.150	.051	.112	-.102	.127
		Sig. (2-tailed)	0.436	0.356	0.011	0.006	0.349	0.038	0.059	0.019
		N	341	341	341	341	341	341	341	341
	Practising holistically to personalise care and promote public and person	Correlation Coefficient	-.015	-.076	.123	-.016	.066	.115	-.162	.159
		Sig. (2-tailed)	0.779	0.164	0.023	0.767	0.226	0.034	0.003	0.003
		N	341	341	341	341	341	341	341	341
	Working with colleagues in primary care	Correlation Coefficient	-.128	-.189**	.066	.069	-.015	.048	-.107	.077
		Sig. (2-tailed)	0.018	0.0005	0.225	0.204	0.783	0.375	0.048	0.156
		N	341	341	341	341	341	341	341	341
	Maintaining an ethical approach and fitness to practice	Correlation Coefficient	-.095	-0.083	0.095	0.139	0.026	0.013	-0.055	.078
		Sig. (2-tailed)	0.081	0.125	0.080	0.010	0.629	0.812	0.312	0.153
		N	341	341	341	341	341	341	341	341
	Information gathering and interpretation	Correlation Coefficient	-.059	-.024	.219**	.062	.066	.125	-.123	.177
		Sig. (2-tailed)	0.280	0.658	0.0000	0.251	0.225	0.021	0.023	0.001
		N	341	341	341	341	341	341	341	341
	Clinical Examination and procedural skills	Correlation Coefficient	-.005	-.079	.079	.142	.106	.081	-.119	.102
		Sig. (2-tailed)	0.924	0.145	0.145	0.009	0.051	0.137	0.028	0.059
		N	341	341	341	341	341	341	341	341
	Making a diagnosis	Correlation Coefficient	.016	-.081	.174	.092	.251**	.196**	-.284**	.231**
		Sig. (2-tailed)	0.763	0.134	0.001	0.090	0.0000	0.0003	0.0000	0.0000
		N	341	341	341	341	341	341	341	341
	Managing medical and clinical complexity	Correlation Coefficient	-.047	-.145	.209**	.017	.170	.156	-.298**	.236**
		Sig. (2-tailed)	0.391	0.007	0.0001	0.750	0.002	0.004	0.0000	0.0000
		N	341	341	341	341	341	341	341	341
	Independent prescribing, medicines, and supply of pharmacotherapy	Correlation Coefficient	-.044	-.091	.334	.097	.357**	.403**	-.757**	.481**
		Sig. (2-tailed)	0.416	0.092	0.0000	0.075	0.0000	0.0000	0.0000	0.0000
		N	341	341	341	341	341	341	341	341
	Leadership and Management	Correlation Coefficient	.016	-.213**	.158	.045	.302**	.175	-.331**	.255**
		Sig. (2-tailed)	0.774	0.0001	0.003	0.403	0.0000	0.001	0.0000	0.0000
		N	341	341	341	341	341	341	341	341
	Education and Development	Correlation Coefficient	-.008	-.095	.105	.003	.143	.087	-.143	.106
		Sig. (2-tailed)	0.888	0.080	0.052	0.956	0.008	0.110	0.008	0.051
		N	341	341	341	341	341	341	341	341
	Research and evidence-based practice	Correlation Coefficient	.000	-.088	.095	.005	.033	.082	-.114	.133
		Sig. (2-tailed)	0.997	0.104	0.079	0.927	0.546	0.131	0.035	0.014
		N	341	341	341	341	341	341	341	341

**. Correlation is significant at the 0.0005 level (2-tailed).

Supplemental Table D. Correlations between the request and interpretation of blood tests and demogaphics of paramedics

			Clinical Supervision	Hours worked	Job Title	Length of time registered as a paramedic	Length of time in primary care	Level of Education	Prescribing Status	Salary
Spearman's rho	ACR	Correlation Coefficient	.093	.232	.094	-.021	.116	.123	.230	.236
		Sig. (2-tailed)	0.085	0.001	0.082	0.705	0.033	0.023	0.001	0.001
		N	341	341	341	341	341	341	341	341
	Amylase	Correlation Coefficient	.077	.118	.150	.059	.115	.198	.306	.266
		Sig. (2-tailed)	0.156	0.030	0.005	0.274	0.033	0.001	0.001	0.001
		N	341	341	341	341	341	341	341	341
	Anti CCP	Correlation Coefficient	.080	.047	.147	.035	.087	.165	.248	.195
		Sig. (2-tailed)	0.139	0.389	0.007	0.514	0.108	0.002	0.001	0.001
		N	341	341	341	341	341	341	341	341
	Autoimmune antibodies	Correlation Coefficient	.100	.084	.129	-.025	.041	.198	.197	.223
		Sig. (2-tailed)	0.065	0.120	0.017	0.649	0.448	0.001	0.001	0.001
		N	341	341	341	341	341	341	341	341
	B12	Correlation Coefficient	.125	.100	.158	-.005	.011	.149	.245	.273
		Sig. (2-tailed)	0.021	0.066	0.003	0.922	0.835	0.006	0.001	0.001
		N	341	341	341	341	341	341	341	341
	BNP/NT-proBNP	Correlation Coefficient	.153	.228	.095	.022	.109	.134	.278	.297
		Sig. (2-tailed)	0.005	0.001	0.081	0.687	0.044	0.013	0.001	0.001
		N	341	341	341	341	341	341	341	341
	CA125	Correlation Coefficient	.092	.161	.148	.005	.115	.186	.268	.257
		Sig. (2-tailed)	0.091	0.003	0.006	0.923	0.034	0.001	0.001	0.001
		N	341	341	341	341	341	341	341	341
	Calcium	Correlation Coefficient	.130	.165	.110	.055	.025	.166	.251	.214
		Sig. (2-tailed)	0.016	0.002	0.043	0.312	0.643	0.002	0.001	0.001
		N	341	341	341	341	341	341	341	341
	Coeliac screen	Correlation Coefficient	.077	.086	.146	-.054	.126	.197	.305	.326
		Sig. (2-tailed)	0.153	0.114	0.007	0.322	0.020	0.0003	0.001	0.001
		N	341	341	341	341	341	341	341	341
	CRP	Correlation Coefficient	.153	.189	.099	.009	.083	.108	.246	.296
		Sig. (2-tailed)	0.005	0.001	0.068	0.871	0.124	0.047	0.001	0.001
		N	341	341	341	341	341	341	341	341
	Drug levels	Correlation Coefficient	.025	.181	.011	.007	.037	.095	.172	.164
		Sig. (2-tailed)	0.641	0.001	0.836	0.895	0.498	0.079	0.001	0.002
		N	341	341	341	341	341	341	341	341
	ESR	Correlation Coefficient	.158	.114	.104	.091	-.028	.158	.189	.201
		Sig. (2-tailed)	0.003	0.036	0.055	0.094	0.600	0.003	0.001	0.001
		N	341	341	341	341	341	341	341	341
	Estosterone	Correlation Coefficient	.009	.090	.044	-.023	.013	.117	.134	.144
		Sig. (2-tailed)	0.869	0.097	0.415	0.671	0.806	0.030	0.013	0.008
		N	341	341	341	341	341	341	341	341
	FBC	Correlation Coefficient	.176	.125	.136	.009	.015	.167	.20	.267
		Sig. (2-tailed)	0.001	0.021	0.012	0.871	0.789	0.002	0.001	0.001
		N	341	341	341	341	341	341	341	341
	Free androgen index	Correlation Coefficient	-.054	.019	.024	-.044	.022	.129	.098	.104
		Sig. (2-tailed)	0.320	0.732	0.657	0.415	0.686	0.017	0.070	0.055
		N	341	341	341	341	341	341	341	341
	FSH/LH +/- prolactin	Correlation Coefficient	.005	.071	.089	.018	.032	.144	.161	.132
		Sig. (2-tailed)	0.920	0.189	0.101	0.746	0.552	0.008	0.003	0.015
		N	341	341	341	341	341	341	341	341
	Glandular fever screen	Correlation Coefficient	.061	.104	.168	.123	.223	.204**	.342**	.324
		Sig. (2-tailed)	0.264	0.056	0.002	0.023	0.001	0.0001	0.0000	0.001
		N	341	341	341	341	341	341	341	341
	Haematinics	Correlation Coefficient	.080	.201	.152	.069	.086	.144	.304	.279
		Sig. (2-tailed)	0.140	0.001	0.005	0.206	0.111	0.008	0.00100	0.001
		N	341	341	341	341	341	341	341	341
	HbA1c	Correlation Coefficient	.155	.142	.118	-.064	.002	.162	.202**	.249**
		Sig. (2-tailed)	0.004	0.009	0.029	0.235	0.971	0.003	0.0002	0.0000
		N	341	341	341	341	341	341	341	341
	Hepatitis	Correlation Coefficient	.076	.087	.127	-.015	.120	.156	.193	.234
		Sig. (2-tailed)	0.164	0.107	0.019	0.779	0.027	0.004	0.0003	0.001
		N	341	341	341	341	341	341	341	341
	HIV	Correlation Coefficient	.045	.069	.137	-.034	.154	.136	.192	.208
		Sig. (2-tailed)	0.407	0.205	0.011	0.526	0.004	0.012	0.001	0.001
		N	341	341	341	341	341	341	341	341
	Iron studies	Correlation Coefficient	.139	.115	.104	-.017	-.004	.135	.162	.198
		Sig. (2-tailed)	0.010	0.034	0.055	0.760	0.938	0.013	0.003	0.0002
		N	341	341	341	341	341	341	341	341
	LFT	Correlation Coefficient	.175	.129	.137	-.006	-.007	.165	.183	.247
		Sig. (2-tailed)	0.001	0.017	0.011	0.911	0.900	0.002	0.001	0.001
		N	341	341	341	341	341	341	341	341
	Lipid profile	Correlation Coefficient	.104	.071	.158	-.003	.077	.192	.278	.341
		Sig. (2-tailed)	0.054	0.192	0.003	0.957	0.157	0.0004	0.001	0.001
		N	341	341	341	341	341	341	341	341
	PSA	Correlation Coefficient	.123	.195	.137	-.008	.002	.205	.297	.293
		Sig. (2-tailed)	0.023	0.001	0.011	0.876	0.977	0.001	0.001	0.001
		N	341	341	341	341	341	341	341	341
	Rheumatoid factor	Correlation Coefficient	.082	.085	.154	.019	.081	.196	.248	.235
		Sig. (2-tailed)	0.130	0.117	0.004	0.727	0.137	0.001	0.001	0.001
		N	341	341	341	341	341	341	341	341
	SHBG	Correlation Coefficient	-.034	.008	.027	.009	.020	.155	.132	.101
		Sig. (2-tailed)	0.530	0.885	0.623	0.867	0.707	0.004	0.014	0.063
		N	341	341	341	341	341	341	341	341
	TFT	Correlation Coefficient	.133	.138	.154	.039	.081	.201**	.276	.276

	Sig. (2-tailed)	0.014	0.011	0.004	0.476	0.136	0.00019	0.001	0.001
	N	341	341	341	341	341	341	341	341
U&Es	Correlation Coefficient	.175	.146	.123	.006	-.007	.152	.185	.261
	Sig. (2-tailed)	0.001	0.007	0.024	0.906	0.898	0.005	0.001	0.001
	N	341	341	341	341	341	341	341	341
Urate	Correlation Coefficient	.141	.193	.122	.063	.133	.167	.256	.288
	Sig. (2-tailed)	0.009	0.001	0.025	0.244	0.014	0.002	0.001	0.001
	N	341	341	341	341	341	341	341	341
Vitamin D	Correlation Coefficient	.142	.090	.095	-.066	-.027	.147	.183	.192
	Sig. (2-tailed)	0.009	0.098	0.078	0.225	0.623	0.006	0.001	0.000
	N	341	341	341	341	341	341	341	341

**. Correlation is significant at the 0.0002 level (2-tailed).

Supplemental Table E. Associations between prescribing status and clinical skills assessments in primary care

Clinical Assessments	Value	df	Asymptotic Significance (2-sided)
Digital rectal examination	68.059	4	215
Eye examination	24.228	4	<.001
Imaging	15.861	4	0.003
Lymphadenopathy	28.75	4	<.001
Mental health examination	14.886	4	0.005
Otoscopy	21.312	4	<.001
Referrals to specialism or specialist services	14.886	4	0.003
Skin samples	37.529	4	<.001
Sputum samples	31.322	4	<.001
Stool samples	24.25	4	<.001
Swabs	32.213	4	<.001
