# Supplementary material for: Understanding the roles and work of paramedics in primary care: a national cross-sectional survey
Source: BMJ Open. 2022 Dec 19;12(12):e067476. doi: 10.1136/bmjopen-2022-067476 (PMC9764645; doi:10.1136/bmjopen-2022-067476)
Supplement: Author's manuscript [file bmjopen-2022-067476.draft_revisions.pdf]

BMJ Open is committed to open peer review. As part of this commitment we make the peer review history of every article we publish publicly available.

When an article is published we post the peer reviewers' comments and the authors' responses online. We also post the versions of the paper that were used during peer review. These are the versions that the peer review comments apply to.

The versions of the paper that follow are the versions that were submitted during the peer review process. They are not the versions of record or the final published versions. They should not be cited or distributed as the published version of this manuscript.

BMJ Open is an open access journal and the full, final, typeset and author-corrected version of record of the manuscript is available on our site with no access controls, subscription charges or pay-per-view fees (http://bmjopen.bmj.com).

If you have any questions on BMJ Open's open peer review process please email info.bmjopen@bmj.com

BMJ Open

Understanding the roles and work of paramedics in primary care:A national cross-sectional survey

Journal:	*BMJ Open*
Manuscript ID	bmjopen-2022-067476
Article Type:	Original research
Date Submitted by the Author:	19-Aug-2022
Complete List of Authors:	Eaton, Georgette; Oxford University, Nuffield Department of Primary Care Health Sciences<br>Tierney, Stephanie; Oxford University, Nuffield Department of Primary Care Health Sciences<br>Wong, Geoff; Oxford University, Nuffield Department of Primary Care Health Sciences<br>Oke, Jason; Oxford University, Nuffiled Department of Primary Care Health Sciences<br>Williams, Veronika; Nipissing University, Faculty of Education and Professional Studies - School of Nursing<br>Mahtani, Kamal; Oxford University, Nuffield Department of Primary Health Care Sciences
Keywords:	PRIMARY CARE, Organisation of health services < HEALTH SERVICES ADMINISTRATION & MANAGEMENT, Quality in health care < HEALTH SERVICES ADMINISTRATION & MANAGEMENT

SCHOLARONE™
Manuscripts

I, the Submitting Author has the right to grant and does grant on behalf of all authors of the Work (as defined in the below author licence), an exclusive licence and/or a non-exclusive licence for contributions from authors who are: i) UK Crown employees; ii) where BMJ has agreed a CC-BY licence shall apply, and/or iii) in accordance with the terms applicable for US Federal Government officers or employees acting as part of their official duties; on a worldwide, perpetual, irrevocable, royalty-free basis to BMJ Publishing Group Ltd ("BMJ") its licensees and where the relevant Journal is co-owned by BMJ to the co-owners of the Journal, to publish the Work in this journal and any other BMJ products and to exploit all rights, as set out in our licence.

The Submitting Author accepts and understands that any supply made under these terms is made by BMJ to the Submitting Author unless you are acting as an employee on behalf of your employer or a postgraduate student of an affiliated institution which is paying any applicable article publishing charge ("APC") for Open Access articles. Where the Submitting Author wishes to make the Work available on an Open Access basis (and intends to pay the relevant APC), the terms of reuse of such Open Access shall be governed by a Creative Commons licence – details of these licences and which Creative Commons licence will apply to this Work are set out in our licence referred to above.

Other than as permitted in any relevant BMJ Author's Self Archiving Policies, I confirm this Work has not been accepted for publication elsewhere, is not being considered for publication elsewhere and does not duplicate material already published. I confirm all authors consent to publication of this Work and authorise the granting of this licence.

For peer review only

TITLE PAGE

Title: Understanding the roles and work of paramedics in primary care: A national cross-sectional survey

Corresponding Author:

Georgette Eaton

Nuffield Department of Primary Care Health Sciences
University of Oxford
Radcliffe Primary Care Building
Radcliffe Observatory Quarter
Woodstock Road
Oxford
OX2 6GG
Georgette.eaton@phc.ox.ac.uk
Tel: +44 (0)1865 617816

Co-Authors:

Dr Stephanie Tierney
Nuffield Department of Primary Care Health Sciences
University of Oxford
Radcliffe Primary Care Building
Radcliffe Observatory Quarter
Woodstock Road
Oxford
OX2 6GG

Dr Geoff Wong
Nuffield Department of Primary Care Health Sciences
University of Oxford
Radcliffe Primary Care Building
Radcliffe Observatory Quarter
Woodstock Road
Oxford
OX2 6GG

Dr Jason Oke
Nuffield Department of Primary Care Health Sciences
University of Oxford
Radcliffe Primary Care Building
Radcliffe Observatory Quarter

For peer review only

Woodstock Road
Oxford
OX2 6GG

Dr Veronika Williams
100 College Drive
Box 5002
North Bay
ON
Canada
P1B 8L7
veronikw@nipissingu.ca
Tel: 705.474.3450

Dr Kamal R. Mahtani
Nuffield Department of Primary Care Health Sciences
University of Oxford
Radcliffe Primary Care Building
Radcliffe Observatory Quarter
Woodstock Road
Oxford
OX2 6GG

For peer review only

Keywords:

1. PRIMARY CARE
2. Organisation of health services
3. Quality in health care

Word Count: 4666

ABSTRACT

OBJECTIVES: This study aimed to collect and analyse data from a national cross-sectional survey with free-text comments to understand the paramedic role in primary care and determine the current working practices of paramedics in this setting.

SETTING The survey was distributed to paramedics in primary care across the UK through the College of Paramedics.

PARTICIPANTS A total of 341 surveys were returned (male = 215). 90% of responses were from paramedics in England, 1.7% from paramedics in Northern Ireland, 4.6% from paramedics in Scotland, and 2.9 from paramedics in Wales. This represents approximately 33% of the primary care paramedic workforce in England and Wales. Estimates for percentages in Northern Ireland and Scotland are unavailable due to the lack of workforce datasets capturing paramedics in primary care.

RESULTS Considerable variation was found in job titles, level of education and provision of clinical supervision of paramedics in primary care. Differing levels of practice were noted, despite guidance documents that attempt to standardise the role. Many respondents outlined their move to work in primary care was to exit the ambulance service and achieve a better work/life balance. However, challenges in relation to access to further education and clinical supervision to support clinical development resulted in frustration for paramedics who work in this setting. Respondents called for improvements in guidance frameworks and legislation shaping the role, alongside support in clinical development.

CONCLUSIONS A standardised clinical role may support the development of paramedics in primary care across the UK, and enable them to work to the full extent of their capability. Based on our findings, we recommend changes to education and support, governance, and legislation to ensure paramedics employed in primary care can work to their full capability.

STRENGTHS AND LIMITATIONS

- This is the first national survey of the paramedic role in primary care within the UK.
- Distributed through the College of Paramedics, and so may not capture the experiences of paramedics not registered with their professional body.
- Survey undertaken during September-November 2021, during which time UK primary care was continuing to respond to the ongoing global pandemic of COVID-19
- The uptake of the survey in Northern Ireland and Scotland was low. Whilst the paramedic role is currently not captured in workforce data for these countries, paramedics in primary care from Northern Ireland and Scotland may be underrepresented in this survey.

For peer review only - http://bmjopen.bmj.com/site/about/guidelines.xhtml

INTRODUCTION

Over the last decade, paramedics in the United Kingdom (UK) have increasingly taken up clinical employment away from ambulance services, moving into primary care settings (1). Reasons for this move are multifactorial and interwoven. Changes to healthcare access has created a sociocultural dependence on the ambulance service (2), resulting in an increase of urgent and primary care related dispositions attended by paramedics (3). To respond to the changing demands of 999 calls, paramedic pre-registration education prepares registrants to deliver holistic care across the lifespan (4), moving beyond protocolised training for the management of emergency presentations, for which paramedics have historically been most well-known. This broad undergraduate education defines the pluripotential nature of paramedics, enabling them to act as generalist clinicians in a range of clinical settings – and ultimately enables them to leave the ambulance service. This, coupled with poor managerial support and a lack of clinical progression within the ambulance service (5), is resulting in paramedics seeking opportunities to work in other clinical settings which offer further development and an improved work-life balance (6). Occurring at a time where the primary care workforce is understaffed due to a failure to recruit and retain General Practitioners (GPs) (7), paramedics actively seek opportunities to work in primary care, believing their capabilities fit well within this clinical setting (8). Indeed, recent NHS workforce policy welcomes this professional group into primary care (9–11), with associated funding through the Additional Roles Reimbursement Scheme (AARS) available for practices employing paramedics (amongst other health and social care staff) in England (12).

Our previous research has highlighted that for paramedics to work successfully as part of the primary care team, transition into this workforce needs to be supported through a combination of formal education, clinical supervision, and socialisation (8). Desired outcomes, such as increasing clinical capacity within the primary care team (and filling workforce gaps created by the shortage of GPs), may then transpire. Health Education England (HEE) has made steps to provide a framework that addresses these concepts. HEE is a non-departmental public body, which provides coordination and support for the training and education within England's healthcare workforce. In 2019, HEE set out a 'roadmap' for paramedics to follow as they transition into primary care

For peer review only - http://bmjopen.bmj.com/site/about/guidelines.xhtml

roles (13). This document outlines the qualifications, capabilities, clinical skills, and case presentations paramedics are expected to encounter whilst working in primary care. This is the only published document across the devolved nations that sets any scope of role for paramedics working in this clinical setting.

Paramedics are being actively recruited into the primary care workforce, but knowledge gaps remain. Ongoing research aims to produce meaningful contributions to inform understanding of the paramedic role in primary care (14). This research specifically aims to fill a current knowledge gap, namely the paramedics' scope of role in primary care and their perception of their roles. This research charts the current scope of clinical role of paramedics in primary care, in relation to specific objectives:

- To investigate the scope of role undertaken by paramedics in NHS primary care.
- To better understand the patterns of education level, experience, salary, prescribing status, and clinical supervision for paramedics in primary care.
- To explore the perceptions paramedics in primary care have on their contribution to primary care teams.

METHODS

Prior to commencing this survey, a study protocol was developed and registered with OSF Registries (10.17605/OSF.IO/YKDA7). We report our findings according to the Strengthening the Reporting of Observational Studies in Epidemiology (STROBE) Statement (14).

Study design

A mixed-method online survey was distributed via the College of Paramedics to paramedics in primary care in England, Northern Ireland, Scotland, and Wales. The survey utilised both qualitative and quantitative items in recognition of the polygonal objectives of the study (15). Details regarding workforce data for paramedics in primary care are reported for England (16) and Wales (17), but not yet for Northern Ireland or Scotland. Based on the available data in England and Wales, we estimated that approximately 1,500 paramedics were working in primary care roles across the

United Kingdom in August 2021. Based on this, a sample size of 306 would be needed for a confidence interval of 95% and margin of error of 5% for statistical analysis.

Materials and procedure

The survey was developed by authors in consultation with members of a patient and public involvement group, and stakeholders from the College of Paramedics, HEE, and the Royal College of General Practitioners. The survey was first piloted with paramedics within this stakeholder group and refined by the authorship team. This pilot assisted in the flow of questions, and the format of question presentation. The final survey was presented using Jisc online surveys (https://www.onlinesurveys.ac.uk/) (online supplemental file 1). Data collection took place between 1st September and 30th November 2021.

Distribution of the survey was initially via internal communications within the College of Paramedics, as well as across their social media platforms. This survey was shared further on social media, in a snowball effect, in an attempt to reach paramedics working in primary care who may not be members of the College of Paramedics.

Respondents were offered a £10 Amazon e-voucher in compensation for their time to complete the survey.

Data analysis

Free-text responses were analysed using semantic level, inductive thematic analysis in NVivo v.12 (18), undertaken by GE. Ten per cent of these codes were reviewed by ST. Quantitative data were analysed using descriptive statistics (mean, SD, and frequencies) and non-parametric tests (Chi-Square Test of Independence, Kruskal-Wallis Test, Mann-Whitney test, Spearman's rho correlation) in IBM SPSS Statistics, Version 28 (IBM Corp., Armonk, N.Y., USA). Statistical analysis was undertaken by GE and reviewed independently by JO.

Quantitative and qualitative data were initially analysed separately, then merged and interpreted (19). During interpretation, data was considered in relation to existing conceptual frameworks (8) to enrich findings and synthesise complementary results.

Patient Involvement

The patient and public group associated with this research were involved at several stages of the research, including the design and analysis of the results.

RESULTS

A total of 341 responses were returned, of which 90.6% were from paramedics working in primary care in England (n=309). Based on workforce data published for the period within which the survey was undertaken (September-November 2021) (16,17), these figures represent 33% of the population for England and Wales separately. Table 1 outlines descriptive characteristics of survey respondents according to age range, gender, and country of work. All respondents answered each question in the survey.

Table 1. Description of survey respondents

**Age Range**	**n**	**Gender**	**n**	**Country of work**	**n**
18-24 years old	4	Female	126	England	309
25-34 years old	98	Male	215	Northern Ireland	6
35-44 years old	113			Scotland	16
45-54 years old	96			Wales	10
55-65 years old	30				

Experience as a paramedic prior to working in primary care

Prior to working in primary care, most respondents had 3-5 years (n=93) or 6-10 years (n=93) experience of working as a paramedic. When asked why they chose to work in primary care, a majority cited an improved work/life balance as the single biggest contributing factor. This was often referred to in relation to a move away from the unsustainable workload in the ambulance service, especially working hours that were considered family friendly, with no night or weekend shifts. Other factors prompting paramedics to take up employment in primary care included the opportunity for clinical development; increased job satisfaction associated with the increased autonomy and enhanced clinical skill set required to work in primary care; and a clinical interest in holistic and preventative medicine associated with general practice.

For peer review only

Level of education

The majority (52%) of respondents were educated to at least one module at FHEQ Level 7/SCQF Level 11, with more than half educated to postgraduate certificate (n=90). Figure 1 outlines the level of education for respondents. The need for additional knowledge to work in primary care was articulated. Respondents often reported a "*constant pressure to study*" (RID 118; Emergency Care Practitioner), and a sense of overwhelm regarding the volume of academic work alongside their clinical workload. Both a lack of protected time to study, as well as absence of funding from primary care employers for paramedics to undertake education qualifications, were highlighted as a hinderance to development in primary care.

Effect of HEE Roadmap

For respondents in England, the 'roadmap' published by HEE (13) was viewed to assist in the standardisation and supervision of the role (n=42), but poorly executed at an employment level, due to lack of clarity in the roadmap's requirements (n=21), ease of fulfilment (n=25), or uptake by employer (n=21). 57 respondents reported the roadmap had no influence on their employment or practice in primary care:

"I'm not ARRS funded so doesn't impact me. I'm aware of the roadmap but my surgery isn't pressuring me to complete it. They know my skill set." (RID 327; Paramedic Practitioner).

Clinical Supervision

85.6% (n=292) of respondents received clinical supervision within their role. When asked to what extent the clinical supervision meets their needs, only 25 respondents had a clinical supervision model that was regular and structured. Other experiences were outlined as "*very poor due to limited time with supervisor*" (RID 51; Paramedic Practitioner).

"I don't feel the GPs really understand the role yet or have the time to support it in a way that would meet the expectations set out by the… roadmap." (RID 199; Paramedic Practitioner).

For peer review only

Job title

Job titles for paramedics working in primary care were varied and inconsistent (Figure 2). Inconsistency in job titles was suggested to contribute to discrepancies in scope of practice and understanding of the role from other health care professionals in primary care:

"I still feel that there is a lack of understanding, or clear delineation between roles… I am unsure if those at my surgery are aware of the difference between a Paramedic Practitioner and Advanced Clinical Practitioner…" (RID 305; Advanced Clinical Practitioner).

As well as patients:

"I don't think [my job title] *reflects my role and is unclear to patients."* (RID 150; Emergency Care Practitioner).

Job title made a difference to the extent to which a diagnosis is made during a consultation (H(3)=15.73, p=<.001), the management of medical and clinical complexity (H(3)=15.73, p=<.001) and leadership and management (H(3)=10.507 p=.015), all of which were increased with seniority of employment.

Salary

The most common salary bracket was between £33,222-£43,041(Figure 3). Significant associations were found between salary and job title (X^2(15)> = 51.137, p= <.001), prescribing status (X^2(5)> = 118.190, p=<.001), highest qualification (X^2(30)> = 72.589, p=<.001), length of time as a paramedic (X^2(40)> = 101.212, p=<.001), and length of time in primary care (X^2(25)> = 112.780, p=<.001).

There was no significant association between salary and gender (X^2(5) = 11.936, p=.036), or salary and region of work within the UK (X^2(15) = 28.589, p=.018).

Length of time working in primary care

Length of time in primary care differed with job title (H(3)=14.145, p=003), where seniority of job title was associated with increased time in primary care. Elements of

the clinical work associated with length of time in primary care are reported in online supplemental file 2.

Hours worked in primary care

Significant associations were found between hours worked and employment type ($X^2(15)$>=129.872, p=<.001), where there was an association found between respondents employed directly with the practice and working full time hours (n=126), and those working in rotational roles between ambulance services and primary care providers working one day a week (n=22).

For peer review only

Core capabilities of paramedics working in primary care

We assessed the relationships between the extent to which the core capabilities of primary care set out within HEE's Roadmap (13) formed part of the respondent's role, against employment type, length of time registered as a paramedic, length of time in primary care, level of education, hours worked, job title, independent prescribing status, and salary. Positive relationships exist between these capabilities and increase in each of these factors – where advanced and consultant level roles would undertake activities related to leadership and management in primary care more frequently. These are outlined in Table A of online supplemental file 2.

However, some respondents experienced frustration regarding their progression within these core capabilities:

"Keen to undertake a leadership role but don't see this being an option. Very much bums on seats." (RID 92; Advanced Clinical Practitioner).

Clinical work undertaken

To understand the breadth and frequency of clinical work undertaken, respondents were asked the extent to which they saw the range of clinical presentations (Figure 4) and the extent to which they undertook the clinical examinations (Figure 5) outlined in HEE's Roadmap (13).

Positive relationships between clinical presentations existed between length of time as a paramedic, length of time in primary care, hours worked in primary care, highest qualification, job title, and prescribing status. Relationships between clinical

examinations undertaken also existed between length of time in primary care, hours worked in primary care, highest qualification, job title, and prescribing status. These results are outlined in Tables B and C in online supplemental file 2.

Blood Tests

The request and interpretation of blood tests by paramedics in primary care was positively influenced by prescribing status, the receipt of clinical supervision, increased salary, hours worked (full time), and higher levels of education. There was no correlation between the request and interpretation of blood tests and length of time as a paramedic, nor length of time in primary care. These results are outlined in Table D online supplemental file 2.

Patient Groups not seen

When asked about patient groups not seen, many respondents (n=189) would not see presentations relating to women's health (including intimate examination, pregnant patients with directly pregnancy related issues, sexual health or menopausal). Other common patient groups not seen by paramedics included children, particularly under the age of two (n=114), and presentations relating to mental health (n=69). In addition, respondents saw clinically complex patients, palliative care, and chronic conditions less than other patient groups.

Emergency Skills

Significant correlations were found between level of education and the undertaking of emergency procedures (rs=--.156, p=<.004, *N*=341), where respondents with lower qualifications undertook emergency procedures to a greater extent. A similar correlation was found between the level of education and attending presentations such as cardiac arrest (rs=-.106, p=.049, *N*=341), catastrophic haemorrhage (rs=-.119, p=.028, *N*=341), shock (rs=-.173, p=.001, *N*=341), cardiovascular adverse signs (rs=-.120, p=<.027, *N*=341), and limp children (rs=.125, p=.021, *N*=341), again where respondents with lower qualifications attended these presentations to a greater extent. This was also reflected in the free-text comments, where some paramedics *"…do not feel challenged by only dealing with same day emergency consultations."* (RID 14; Paramedic).

There was also a correlation between hours worked and attending presentations such as catastrophic haemorrhage (rs=.109, p=.044, *N*=341), anaphylaxis (rs=.127, p=.019, *N*=341), angioedema (rs=.140, p=.009, *N*=341), seizures (rs=.147, p=.007, *N*=341), and overdose/poisoning (rs=.200, p=<.001, *N*=341), where respondents who worked less hours in primary care (such as one day a week or 10-20 hours per week) attended these presentations to a greater extent. It was also noted that these respondents were employed primarily by ambulance services, and so likely to be working in rotational roles.

Free-text comments outlined from respondents who had been employed for a longer time in primary care missed the opportunity to attend higher acuity patients, and also reported a loss of paramedic identity without the opportunity to practice their emergency skills.

Prescribing status

125 respondents (36.7%) were independent prescribers, with a further 57 (16.7%) undertaking the course at the time of the survey, and a further 137 (38.7%) wishing to undertake the course in the future. Inability to prescribe frustrated 20 respondents when asked about the frustrations in their role, though many added that they had plans to complete this module of study.

Significant associations were found between prescribing status and clinical assessment skills outlined in Table E online supplemental file 2, where independent prescribers were more likely to undertake these examinations when compared to paramedics who could not prescribe.

Role frustrations

Short appointment times and volume of patients within the day contributed to high workload and feelings of frustrations about their role in primary care. General system frustrations were also reported regarding the structure, organisation, and funding of primary care. In addition, the impact of the ongoing global pandemic of coronavirus disease (COVID-19) on the working practices of respondents caused frustration, with experiences of an increase in telephone consultation and aggression from patients.

For peer review only - http://bmjopen.bmj.com/site/about/guidelines.xhtml

Respondents reported frustration when faced with restrictions in practice, commonly due to an inability to prescribe Schedule 2 controlled medicines, or to provide patients with a Statement of Fitness for Work. Other restrictions in practice included an ability to fit contraceptive devices, or certain procedural skills such as undertaking intimate examination.

Another frustration reported was a lack of understanding of the capabilities of the paramedics in primary care, from both colleagues and patients:

"Sometimes there are major misconceptions about what paramedics can and can't do. Most management have no idea about the role of a paramedic." (RID 326; First Contact Practitioner Paramedic).

"I am well qualified for my role, I am experienced. But I work alongside doctors who do not understand my capabilities." (RID 15; Advanced Clinical Practitioner, Paramedic).

Role gratification

When asked about the difference respondents felt they made in the role, this was considered between the difference they felt they made in the primary care workforce, the difference to patients, and the difference their role had on the paramedic profession overall.

Primary care

Just under half of respondents felt their role in primary care increased workforce capacity (n=161, 47%). This was in the context of freeing up time "*for GPs to manage complex conditions*" (RID 9; Advanced Paramedic Practitioner); facilitating multi-disciplinary team working, and being able to capitalise on their unique paramedic skills:

"*Our clinical knowledge with ability to make autonomous safe decisions with the ability to recognise big sick / little sick quickly means that we are a strong spoke in the wheel of general practice."* (RID 303; Specialist Paramedic).

For peer review only

Patients' satisfaction

Respondents measured the satisfaction of patients who they had seen in their role by virtue of positive feedback received, and patients who actively sought consultations with them again in the future or did not reattend for the same presenting complaint. This patient satisfaction was attributed to the comprehensive consultations undertaken by paramedics, which they reported as benefitting from increased time slots, and the discussion of medical conditions in a simple language patients could understand. Indeed, paramedics viewed their interpersonal skills as one of their greatest strengths regarding patient satisfaction:

"*For the most part people are happy to see a professional that listens to them, treats them with respect and dignity and is competent. I think a lot of paramedics offer this in primary care."* (RID 135; Advanced Clinical Practitioner).

Experiences of patient dissatisfaction following consultation by a paramedic in primary care was also reported by respondents. This occurred when patients were disappointed that the consultation was not with a GP, and where there was a lack of understanding of the paramedic role, leading them to question the clinical expertise paramedics could offer:

"Some patients do not understand my role/skill set and just want a GP." (RID 143; Advanced Paramedic).

Profession

Respondents considered their role in primary care in relation to promotion and understanding of the profession, which "*shows that there is so much more than being a paramedic on the back of an ambulance*" (RID 144; Paramedic Practitioner). Other respondents considered this in relation to the development of the profession as a whole:

"I feel as though the level of autonomous clinical decision making, management of complex cases, referrals and medicine management we are practising is at a much higher level than a large amount of the profession. I think it sets a good example to other paramedics and other medical colleagues alike that paramedics can be pushed

For peer review only - http://bmjopen.bmj.com/site/about/guidelines.xhtml

beyond traditional roles and expectations and can prove to be valuable part of clinical team." (RID 202; Advanced Paramedic).

The impact of the move to work in primary care was also acknowledged in context of the wider healthcare workforce. Respondents outlined this both in terms of leaving the ambulance service to undertake roles in primary care, as well as rotational working between ambulance services and primary care settings. This was considered to have benefit regarding the transferability of clinical knowledge and skills from one clinical setting to the other:

"My rotational role increases understanding between ambulance & primary care, increases my knowledge & understanding which I can pass on to colleagues and increases visibility, trust and understanding of the profession throughout primary care and the lay community." (RID 36; Specialist Paramedic).

For peer review only

DISCUSSION

Main findings of this study

This research confirms previous publications which noted variance in a) job title reported by paramedics working in primary care; b) the clinical work and examinations undertaken by paramedics in this setting; c) entry requirements in terms of clinical experience and education to work in primary care (1,8). This level of variation subsequently leads to confusion around the scope and expectations for the role and contributes to a lack of recognition of paramedics within primary care teams. Whilst attempts have been made through HEE's Roadmap (13) to outline a framework to address this, this is applicable only in England, with minimal uptake by paramedics across the devolved nations for whom such a framework has no influence. The main barriers to engagement with this framework were competing workload pressures affecting the delivery of clinical supervision and uptake of this framework by primary care employers. Such inconsistency contributed to frustration and demotivation among respondents, who were concerned regarding their clinical development in this setting.

Clinical examinations and procedural skills

Our analysis indicates that length of time in primary care, the presence of clinical supervision, higher levels of education, and status as an independent prescriber all contribute to an increase in the scope of role for paramedics in primary care. Indeed, an inverse association was also observed, where paramedics with lower educational qualifications attended emergency presentations within primary care to a greater extent than those who had undertaken higher education. This outlines that, whilst the paramedic may transition into primary care due to the virtue of their generalist background, their productivity in primary care may be influenced by further education and feedback regarding their clinical experience in this setting. Despite this generalist background, this survey also outlines there are patient groups commonly not seen by paramedics. This could be due to policies that emphasise the need for nurses to see women who are pregnant in this setting (20), and a denouement from guidance for paramedics in ambulance services to convey all children under the age of two (21).

The survey also highlighted that paramedics working less hours in primary care (such as one day a week or 10-20 hours per week) attended emergency presentations in their primary care role to a greater extent when compared to their full-time counterparts. Such hours are common in rotational models, where paramedics, specialist paramedics or advanced paramedics work in a split clinical role between ambulance services and primary care settings in an attempt to increase workforce capacity in primary care and reduce attrition from the ambulance service (22). Whilst the ability for paramedics to attend emergency presentations in primary care may be a benefit for primary care providers, this does little to develop their primary care clinical acumen.

Paramedic taxonomy

Some job titles reported by respondents match those endorsed by the College of Paramedics (23) (such as ‘Paramedic’, ‘Specialist Paramedic’, ‘Advanced Paramedic’, or ‘Consultant Paramedic’) or those outlined by HEE (13) (such as ‘First Contact Practitioner’ and ‘Advanced Practitioner’), yet there remains a variety of job titles that do not correlate to these archetypes.

For peer review only

Our analysis indicates that, as paramedics take up more senior roles in primary care (such as Advanced Paramedic), their scope of role increases in relation to clinical examinations performed, and the clinical presentations they attend. Such an increase in scope could be due to their ability to independently prescribe and undertaking postgraduate study. Independent prescribing is typically undertaken by advanced paramedics who have completed (or working towards completion of) a Master's degree (24). We noted that these paramedics are more likely to make a diagnosis during the consultation and manage medical and clinical complexity. This is in contrast to 'paramedics' or 'first contact practitioners', who may have a similar scope of clinical examination, but a reduced scope in relation to managing clinical complexity and making a diagnosis. This supports previous findings where such paramedics are employed in an 'eye and ears' approach only (8).

There was a strong correlation with advanced and consultant level roles and undertaking activities related to leadership and management in primary care. This suggests that paramedics may move into leadership roles within primary care that have traditionally been filled by GPs. However, there was no correlation regarding undertaking research activities and job title. This indicates that research activities are less accessible to paramedics in primary care, despite being a pillar of advanced practice, matching previous research findings (25).

Strengths and weaknesses of the study

This is the first national survey of the paramedic role in primary care within the UK. It has international relevance for primary care workforce transformation in countries where paramedics operate in a similar way to in the UK, such as in Australasia and Canada (26). The use of a mixed methods cross-sectional survey allowed for the exploration of both the distribution and variety of roles paramedics undertake in primary care, with tentative claims made regarding the impact clinical exposure in primary care, education, ability to prescribe and clinical supervision has on the paramedic role. Free-text responses also enabled further understanding of the support and frustrations paramedics experience when working in primary care.

Whilst the survey was widely distributed across each UK nation, this was either through the College of Paramedics, or on social media – and thus paramedics not

For peer review only

registered with the professional body, or not on social media, may not have had access. It is noted that the uptake of the survey in Northern Ireland and Scotland was low. Numbers of paramedics working in primary care roles is likely to be fewer than in England, however the paramedic role is currently not captured in workforce data for these countries (27,28), so the actual number of paramedics in primary care in these countries is unknown. There was no variation by region reported in the clinical work undertaken, outlining that paramedics are working in a similar way across the UK. Nevertheless, we recognise that paramedics from Northern Ireland and Scotland may be underrepresented in this survey.

It is appreciated that data collection took part during the ongoing global pandemic of COVID-19. As this is the first national survey of the paramedic role in primary care, it is unclear what impact this ongoing pandemic has on these results. Future surveys may be able to determine this.

RECOMMENDATIONS

Based on our findings above we make a number or recommendations:

Education and support

• There is a need to further standardise required education and training for paramedics working in primary care.

• There should be systems in place within primary care settings to ensure paramedics have access to structured clinical supervision.

• Support should be given to employers to enable them to effectively support the paramedic in primary care and utilise this healthcare professional to the greatest benefit.

Governance

• There is a need to standardise job titles, salaries, and descriptions for paramedics working in primary care to ensure role recognition by both patients and the wider primary care team.

• A standard scope of role for paramedics working in primary care that is applicable to all nations is needed.

For peer review only

• Clear career pathways for paramedics working in primary care should be established, to maximise retention and job satisfaction in this clinical setting.

Changes to legislation

• Changes to legislation to support independent prescribing of controlled medications.

• Ability for paramedics to provide a Statement of Fitness for Work.

CONCLUSION

As the first large-scale survey of the paramedic role in primary care across the UK, this study highlights the types of patient groups seen by this workforce, and the extent to which these are managed. As well as offering an insight into the clinical scope of role undertaken in this setting, we have highlighted relationships that exist between clinical exposure in primary care, level of education, ability to prescribe medicines and provision of clinical supervision for paramedics to work in primary care.

For peer review only

FIGURE LEGEND

Figure 1. Level of education of respondents

Figure 2. Job title of respondents.

Figure 3. Salary of respondents

Figure 4. The frequency of clinical presentations attended by respondents as a paramedic in primary care

Figure 5. The frequency of clinical examination undertaken by respondents as a paramedic in primary care

For peer review only

ACKNOWLEDGEMENTS

We would like to thank the patient and public group involved with this research for their time and comments during the design of the survey and the analysis of the results. We would also like to thank our stakeholders from the College of Paramedics, Health Education England, the Nuffield Trust, and the Royal College of General Practitioners with whom we discussed the development of our survey, and with whom we piloted the survey.

Contributors

GE, ST, GW, KM, VW designed the survey. Data analysis was carried out by GE, JO and ST. The manuscript was drafted by GE, ST, GW, JO, VW and KM. All authors accept full responsibility for the work and/or the conduct of the study, had access to the data, and controlled the decision to publish. All authors read and approved the final manuscript

Funding

GE is supported by a National Institute for Health Research (NIHR) Doctoral Research Fellowship [NIHR300681]. This work is also supported by NHS Health Education England [ref: 190121].

Disclaimer

The views expressed are those of the authors and not necessarily those of the NHS, the NIHR, NHS Health Education England or the host institution. The funders of this research remained independent of the project design, data collection and data analysis.

Competing Interests

GE is a Trustee in the College of Paramedics. The recruitment of paramedics to the survey was made by application to the College of Paramedics Research Design Advisory Committee and was not associated with GE's trusteeship.

The remaining authors declare no competing interests.

Ethics approval

For peer review only

This study was reviewed by the Central University Research Ethics Committee (MS IDREC Ref: R64129/RE001. Respondents were free to decide whether to participate. Compensation was offered at completion of the survey, in the form of a £10 Amazon e-voucher. All data processing and storage comply with the General Data Protection Regulation and UK Data Protection Act 2018.

Data availability statement

Datasets are available upon reasonable request.

For peer review only

REFERENCES

1. Eaton G, Wong G, Williams V, Roberts N, Mahtani KR. Contribution of paramedics in primary and urgent care: A systematic review. *Br J Gen Pract* 2020; 70 (695): e421-e426
2. Wankhade P. Cultural characteristics in the ambulance service and its relationship with organisational performance: evidence from the UK. In: Does Culture matter Track. Nottingham: PAC Conference: 6-8 September; 2010.
3. Eaton G, Mahtani K, Catterall M. The evolving role of paramedics – a NICE problem to have? *J Health Serv Res Policy* 2018 Jul;23(3):193-195.
4. College of Paramedics. Paramedic Curriculum Guidance. 5th ed. Bridgwater: College of Paramedics; 2019. https://collegeofparamedics.co.uk/COP/ProfessionalDevelopment/Paramedic_Curriculum_Guidance.aspx (accessed 20 July 2022)
5. Beldon R, Garside J. Burnout in frontline ambulance staff. *JPP* 2021 Dec 30;14(1):6–14.
6. Mahtani KR, Eaton G, Catterall M, Ridley A. Setting the scene for paramedics in general practice: what can we expect? *J R Soc Med* 2018;111(6).
7. Marchand C, Peckham S. Addressing the crisis of GP recruitment and retention: a systematic review. *Br J Gen Pract* 2017];67(657):e227–37.
8. Eaton G, Wong G, Tierney S, Roberts N, Williams V, Mahtani KR. Understanding the role of the paramedic in primary care: a realist review. *BMC Med* 2021;19(1)
9. NHS England. The NHS Long Term Plan. London, NHS England 2019
10. NHS England. General Practice Forward View. London, NHS England 2016.
11. NHS England. Supporting General Practice in 2021/22. London, NHS England 2021.
12. NHS Confederation. Supporting population health management: workforce Recruiting paramedic practitioners through the Additional Roles Reimbursement Scheme (ARRS). 2021 https://www.nhsconfed.org/-/media/Confederation/Files/Publications/Documents/Paramedic-practitioners-ARRS.pdf (accessed 20 July 2022)
13. Health Education England. First Contact Practitioners and Advanced Practitioners in Primary Care: (Musculoskeletal) A Roadmap to Practice. London, Health Education England 2021.

For peer review only

14. Von Elm, E., Altman, D. G., Egger, M., Pocock, S. J., Gøtzsche, P. C., Vandenbroucke, J. P., & STROBE Initiative. The Strengthening the Reporting of Observational Studies in Epidemiology (STROBE) statement: guidelines for reporting observational studies. *Lancet* 2007 370(9596), 1453–1457. https://doi.org/10.1016/S0140-6736(07)61602-X
15. Czaja R, Blair J, Blair E. Designing surveys: A guide to decisions and procedures. LODNON, SAGE Publications 2014.
16. NHS Digital. Headcount and Full-Time Equivalent (FTE) Time Series. General Practice Workforce: 2022 https://app.powerbi.com/view?r=eyJrIjoiYTM4ZTA3NGItMTM2Mi00NzAwLWEyY2QtNDgyZDkxOTk3MmFlIiwidCI6IjUwZjYwNzFmLWJiZmUtNDAxYS04ODAzLTY3Mzc0OGU2MjllMiIsImMiOjh9 (accessed 20 July 2022)
17. Welsh Government. Number of wider practice staff employed in general practices, by staff category and local health board. 2022 https://statswales.gov.wales/Catalogue/Health-and-Social-Care/General-Medical-Services/number-of-wider-practice-staff-employed-in-general-practices (accessed 20 July 2022)
18. Braun V, Clarke V. Thematic Analysis A Practical Guide. London: SAGE Publications 2022.
19. Zheng M. Conceptualization of Cross-Sectional Mixed Methods Studies in Health Science: A Methodological Review. *Int j quant qual res* 2015; 3(2):66–87.
20. Royal College of Nursing. Advanced Level Nursing Practice and Care of Pregnant and Postnatal Women. London; 2021.
21. National Ambulance Service Medical Directors. NASMeD Best Practice guidance: Conveyance of children by operational ambulance clinicians in face-to-face settings. London; 2021
22. Welsh Ambulance Services NHS Trust. Advanced Paramedic Practitioner Pilot-BCU Health Board Final Report. 2020 https://aace.org.uk/wp-content/uploads/2020/08/APP-Rotational-Pilot-Report.pdf (accessed 20 July 2022)
23. College of Paramedics. Post-Registration Career Framework. 4th ed. 2018 https://collegeofparamedics.co.uk/COP/ProfessionalDevelopment/post_reg_career_framework.aspx (accessed 20 July 2022)

For peer review only - http://bmjopen.bmj.com/site/about/guidelines.xhtml

24. College of Paramedics. Practice Guidance for Paramedics Independent & Supplementary Prescribers 2021 https://collegeofparamedics.co.uk/COP/Professional_development/Medicines_and_Independent_Prescribing/COP/ProfessionalDevelopment/Medicines_and_Independent_Prescribing.aspx?hkey=04486919-f7b8-47bd-8d84-47bfc11d821a (accessed 20 July 2022)
25. Fothergill J, Al-Oraibi A, Houdmont J, Conway J, Evans C, Timmons S, et al. Nationwide evaluation of the advanced clinical practitioner role in England: a cross-sectional survey. *BMJ Open* 2022; 12:55475 http://dx.doi.org/10.1136/bmjopen-2021-055475 (accessed 20 July 2022)
26. O'Meara P. Community paramedics: a scoping review of their emergence and potential impact. *International Paramedic Practice* 2014;4(1):5–12
27. Northern Ireland Statistics and Research Agency. General Medical Services for Northern Ireland, Annual Statistics 2019/20. Belfast; 2020 https://www.statisticsauthority.gov.uk/osr/code-of-practice/ (accessed 20 July 2022)
28. Public Health Scotland. General Practice Workforce Survey 2021 https://publichealthscotland.scot/media/9866/2021-10-26-gpworkforcesurvey2021-report.pdf (accessed 20 July 2022)

For peer review only

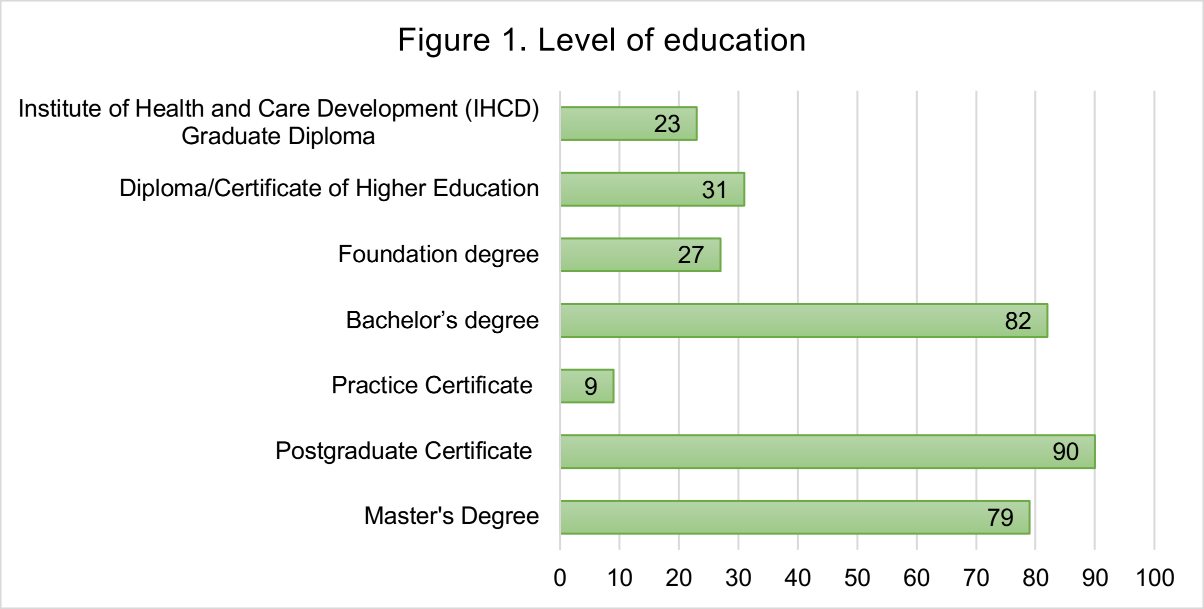

peer review only

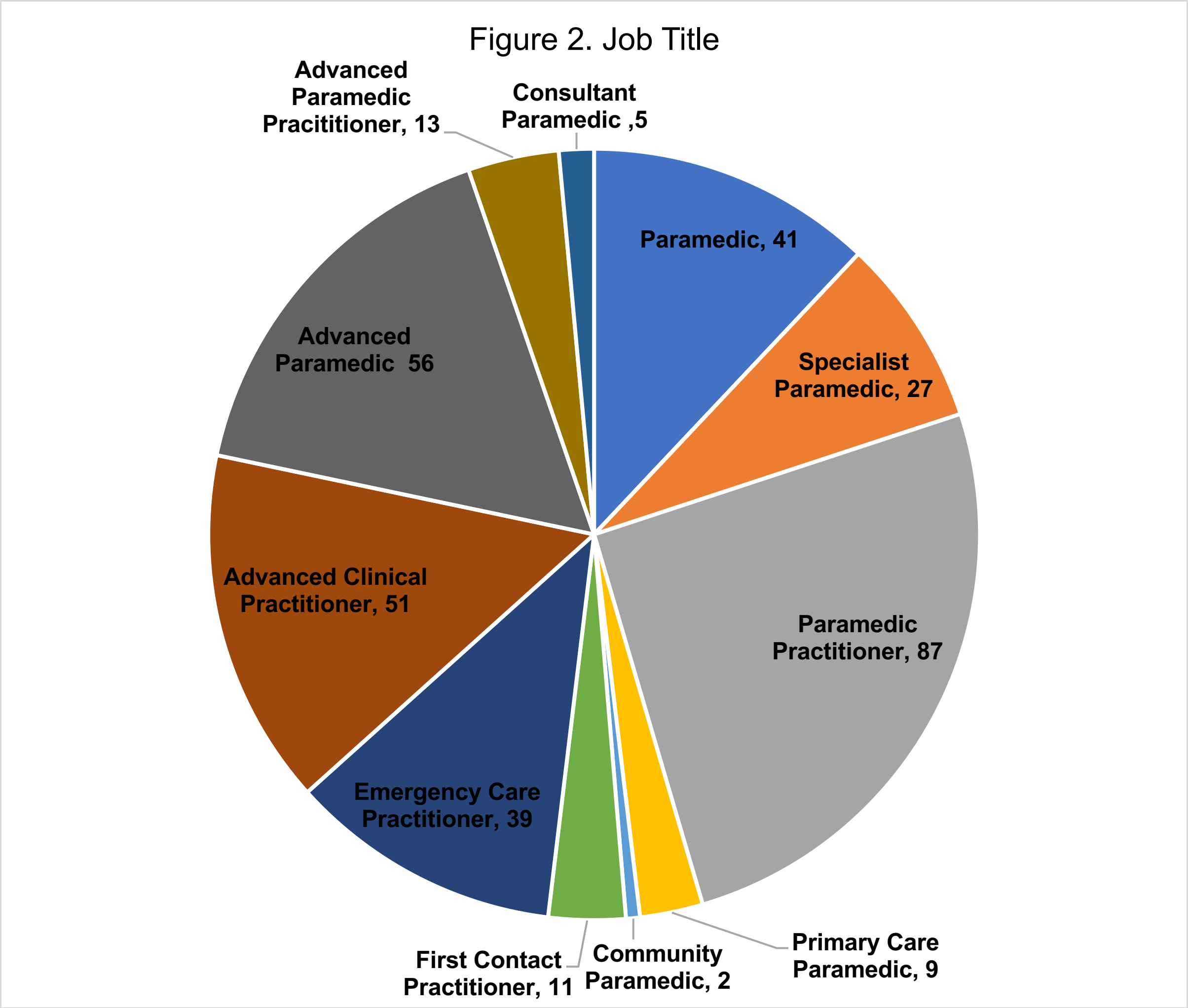
Figure 2. Job Title
Advanced Paramedic Pracititioner, 13
Consultant Paramedic ,5
Paramedic, 41
Specialist Paramedic, 27
Paramedic Practitioner, 87
Primary Care Paramedic, 9
Community Paramedic, 2
First Contact Practitioner, 11
Emergency Care Practitioner, 39
Advanced Clinical Practitioner, 51
Advanced Paramedic 56

Figure 3. Salary

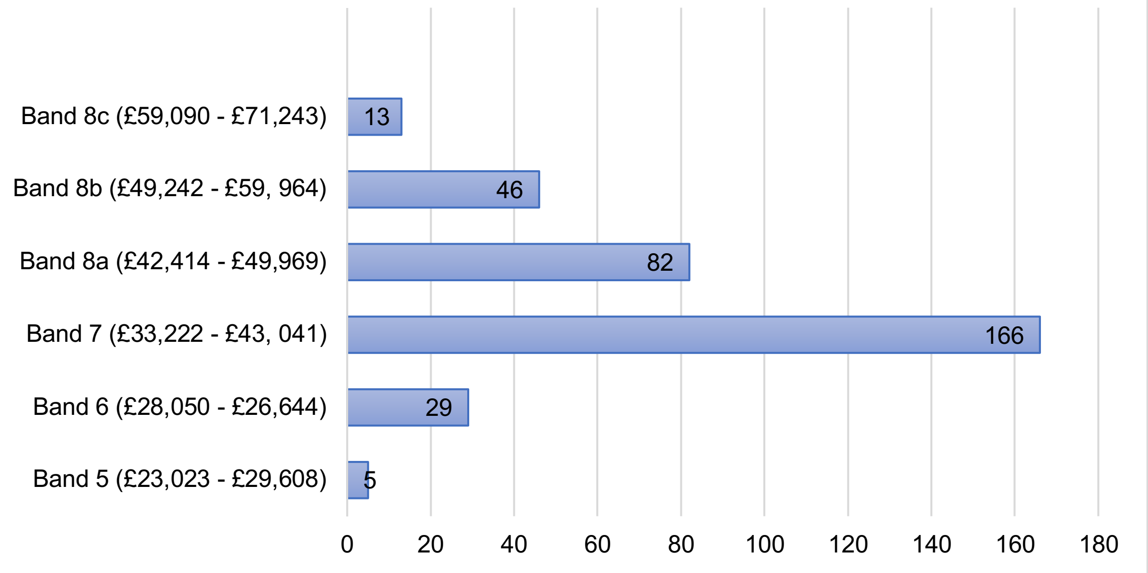

peer review only

Figure 4. Frequency of Clinical Presentations attended by Paramedics working in Primary Care

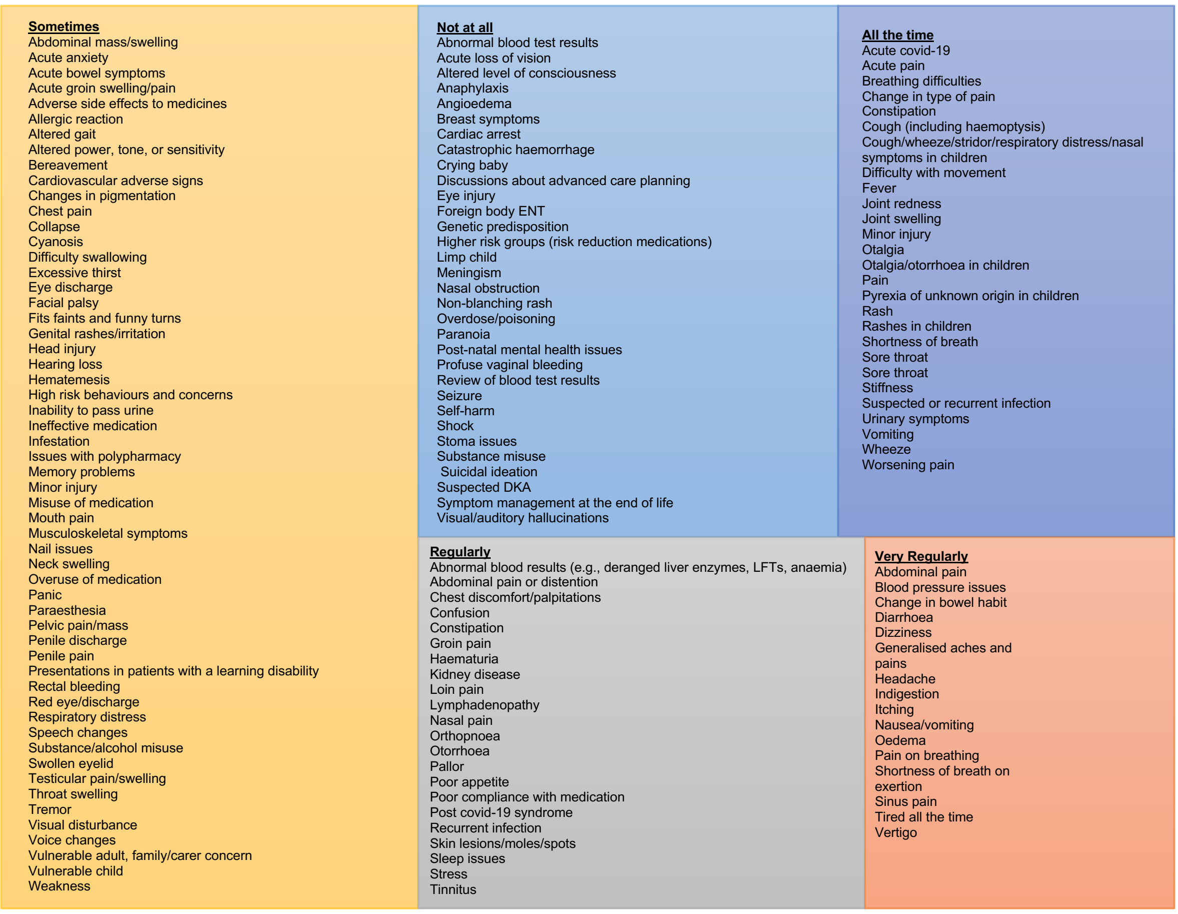

For peer review only - http://bmjopen.bmj.com/site/about/guidelines.xhtml

Figure 5. Frequency of Clinical Examinations undertaken by Paramedics working in Primary Care

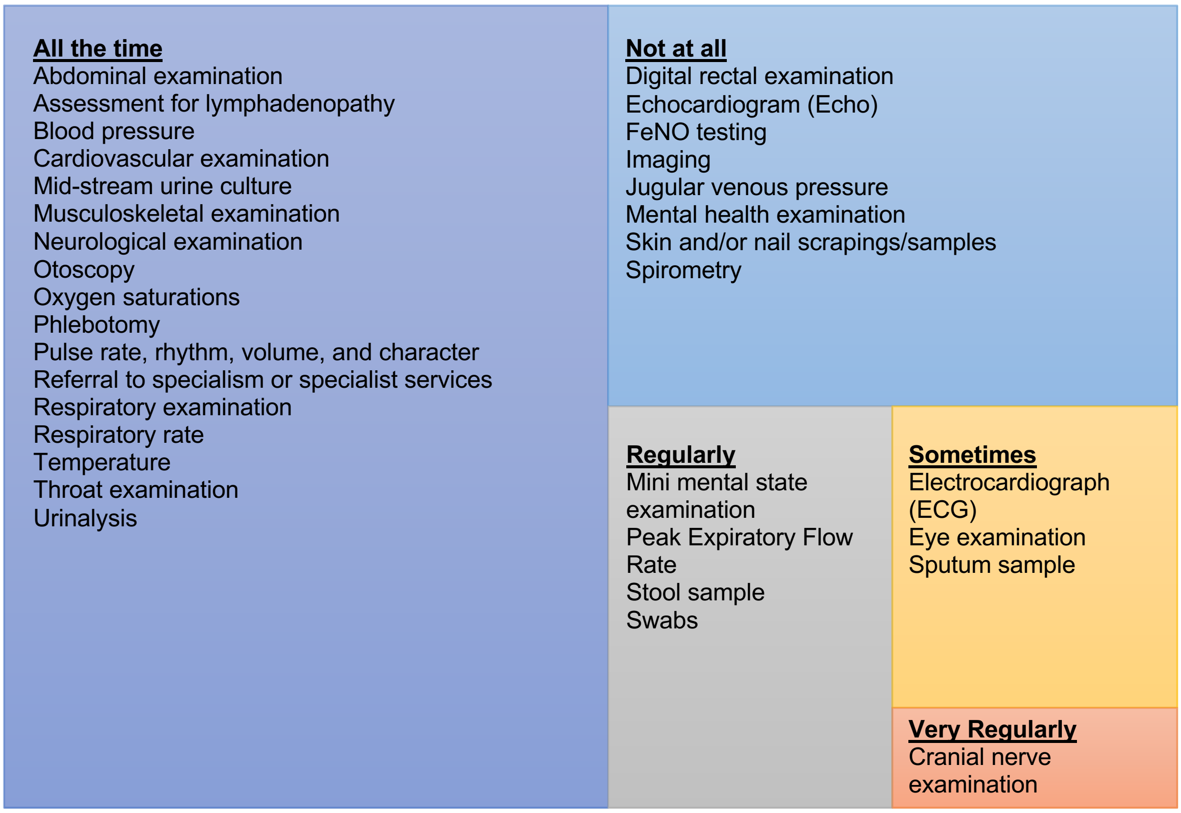

eview only

SURVEY QUESTIONNAIRE

Understanding the roles and work of paramedics in primary care: A national cross-sectional survey

What is this project about?
Thank you for your interest in participating in this questionnaire. This questionnaire is part of a study which aims improve understanding of the ways in which paramedics are working within the primary care workforce in the United Kingdom. Whilst paramedics are currently working in primary care roles, evidence must be generated to show how and why these changes would work, for whom, in what context and to what extent. That's why we're keen to understand from your perspective, as a paramedic working in primary care, the impact you feel you have and your perspectives of your role. We hope that the results of our research will influence future policy and professional change, as well as support paramedics working in primary care in the future.

You have been invited to participate as you are a paramedic working in primary care within the UK. Please read through this information before agreeing to participate (if you wish to) by ticking the 'yes' box below.

You may ask any questions before deciding to take part by contacting the researcher (details below).

The Principal Researcher is Georgette Eaton, who is attached to the Nuffield Department of Primary Care Health Services at the University of Oxford. This project is being completed under the supervision of Dr Kamal R. Mahtani, Dr Geoff Wong, Dr Stephanie Tierney and Dr Veronika Williams from the University of Oxford, and Professor Julia Williams from the University of Hertfordshire. It is funded through a Doctoral Research Fellowship from the National Institute of Health Research.

You will be asked a series of questions about the work you undertake in primary care, and your perceptions of this. This should take about 20 minutes. No other background knowledge is required.

Do I have to take part?
No. Please note that participation is voluntary. If you do decide to take part, you may withdraw at any point for any reason before submitting your answers by pressing the 'Exit' button/ closing the browser. We are offering every paramedic who completes the questionnaire a £10 Amazon e-voucher. However, we are only able to offer this to participants who complete all study activities.

We have included a 'Prefer not to say' option for each set of questions should you prefer not to answer a particular question.

How will my data be used?
During the main set of questions, we will not collect any data that could directly identify you.

At the end of the survey, you will see a page to register your interest in being part of the second phase of our research which will involve an interview. To register your interest, please submit your name and nhs.net email address. This is not linked to your completion of the questionnaire and there is no obligation to be involved in the second phase of our research.

We are offering every paramedic who completes the questionnaire a £10 Amazon e-voucher. At the end of the survey, you will be taken to a separate page to give us your

SURVEY QUESTIONNAIRE

name and nhs.net email address in order to claim this e-voucher. This cannot be matched to your responses in the survey and will be destroyed when individual participant involvement is complete (e.g. when the Amazon e-voucher has been sent and redeemed by you.

Your IP address will not be stored. We will take all reasonable measures to ensure that data remain confidential.

The responses you provide will be stored in a password-protected electronic file on University of Oxford secure servers and may be used in academic publications, conference presentations, reports for external organisations and on websites. Identifiable information will be deleted as soon as it is no longer required for the research. Research data will be stored for 3 years after publication or public release of the study's results.

Who will have access to my data?
The University of Oxford is the data controller with respect to your personal data (name and email address) and, as such, will determine how your personal data is used in the study. The University will process your personal data for the purpose of the research reasons outlined above. Research is a task that we perform in the public interest. Further information about your rights with respect to your personal data is available from https://compliance.admin.ox.ac.uk/individual-rights.

All data will be anonymous. The answers you give us will be shared with the College of Paramedics, Health Education England, and the National Institute for Health Research.

We would also like your permission to use the questionnaire answers in future studies, and to share these with other researchers (e.g., in online databases). This will not include your name and email address, as this is only to claim incentive for the study or to participate in future work associated with this project.

The results will be written up for a DPhil (PhD) degree.

Who has reviewed this study?
This project has been reviewed by, and received ethics clearance through, a subcommittee of the University of Oxford Central University Research Ethics Committee R64129/RE001.

Who do I contact if I have a concern or I wish to complain?
If you have a concern about any aspect of this study, please speak to Georgette Eaton at georgette.eaton@phc.ox.ac.uk or their supervisor, Professor Kamal R. Mahtani kamal.mahtani@phc.ox.ac.uk and we will do our best to answer your query. We will acknowledge your concern within 10 working days and give you an indication of how it will be dealt with. If you remain unhappy or wish to make a formal complaint, please contact the Chair of the Research Ethics Committee at the University of Oxford who will seek to resolve the matter as soon as possible.

Please note that you may only participate in this survey if you are 18 years of age or over	Yes<br>No
If you have read the information above and agree to participate with the understanding that the data (including personal data) you submit will be processed accordingly, please tick the box below to start.	Yes<br>No

SURVEY QUESTIONNAIRE

**Section 1.** The purpose of this section is to find out about the prevalence of paramedics working in primary care in the United Kingdom.	
Are you currently a paramedic working in primary care?	Yes<br>No
What is your age range?	18-24 years old<br>25-34 years old<br>35-44 years old<br>45-54 years old<br>55-65 years old
Which of the following most accurately describe(s) you?<br><br>(Multiple choice answer)	Female<br>Intersex<br>Male<br>Non-binary<br>Transgender<br>Let me type (answer box)
Where do you practice?	England<br>Northern Ireland<br>Scotland<br>Wales
How long have you been a registered paramedic?	0-2 years<br>3-5 years<br>6-10 years<br>11- 14 years<br>15 - 19 years<br>20-24 years<br>25 - 30 years<br>31-35 years<br>>36 years
How long have you worked in your current role in primary care?	0-6 months<br>6-12 months<br>12 – 18months<br>18months – 2 years<br>2-4 years<br>5-7 years<br>8-10 years<br>11- 14 years<br>15 - 19 years<br>>20 years
What is your salary?	Band 5 (£24,907 - £30,615)<br>Band 6 (£31,365 - £37,890)<br>Band 7 (£38,890 - £44,503)<br>Band 8a (£45,753 - £51,668)<br>Band 8b (£53,168 - £62,001)<br>Band 8c (£63,751 - £73,664)<br>Band 8d (£75,914 - £87,754)<br>Band 9 (£91,004 - £104,927)

For peer review only

SURVEY QUESTIONNAIRE

What hours do you work in primary care?	Full time – more than 30 hours per week<br>Part time – 21-30 hours per week<br>Part time – 10-20 hours per week<br>One day a week<br>One session a week (4 hours)
What is your highest qualification relevant to your role as a paramedic in primary care?	Institute of Health and Care Development (IHCD) Graduate Diploma<br>Diploma/Certificate of Higher Education<br>Foundation degree<br>Bachelor's degree<br>Practice Certificate<br>Postgraduate Certificate<br>Postgraduate Diploma<br>Master's Degree<br>Let me type (answer box)
What is your job title?	Paramedic<br>Specialist Paramedic<br>Paramedic Practitioner<br>First Contact Practitioner (Paramedic)<br>Emergency Care Practitioner<br>Advanced Clinical Practitioner (Paramedic)<br>Advanced Paramedic<br>Consultant Paramedic<br>Let me type (answer box)
Are you annotated as an independent prescriber with the Health and Care Professions Council?	Yes<br>Not yet – I am undertaking the course currently<br>No – but I would like to be in the future<br>No
How are you employed in primary care?	Directly employed by GP practice<br>Directly employed by a primary care network/federation/health board<br>Freelance/locum contract<br>Agency/Private provider<br>Rotational through ambulance trust<br>Let me type (answer box)
What type of tasks do you undertake*?<br><br>*These could be face to face or remotely (by telephone or online)<br><br>Please select as many as apply.	Same day/urgent home visits<br>Routine home visits<br>Same day/urgent clinic appointments*<br>Minor illness clinic*<br>Routine clinic appointments*<br>Telephone triage<br>Care home ward rounds<br>Chronic Disease Reviews*<br>Covid clinics*

For peer review only

SURVEY QUESTIONNAIRE

	Admin (e.g. requesting investigations, interpreting results, medication checks) Let me type (answer box)
What types of patients do you see?	Let me type (answer box)
Are there any patient types that you do not see?	Let me type (answer box)
Why?	Let me type (answer box)

For peer review only

SURVEY QUESTIONNAIRE

Section 2.
The purpose of this section is to understand the type of activities that make up your role as a paramedic in primary care. For each of the following activities, please indicate the extent that these form part of your role

	Not at all	Little extent	Some extent	Great extent	Very great extent
Communication and consultations					
Practising holistically to personalise care and promote public and person health					
Working with colleagues in primary care					
Maintaining an ethical approach and fitness to practice					
Information gathering and interpretation					
Clinical Examination and procedural skills					
Making a diagnosis					
Managing medical and clinical complexity					
Independent prescribing, medicines, and supply of pharmacotherapy					
Leadership and Management					
Education and Development					
Research and evidence-based practice					

For peer review only

SURVEY QUESTIONNAIRE

Section 3.
The purpose of this section is to understand the type of clinical presentations that make up your role as a paramedic in primary care. For each of the following activities, please tick the regularity of which you see these in your role in primary care

	Not at all	Sometimes	Regularly	Very regularly	All the time
**Cardiovascular system*** Blood pressure issues, chest pain, chest discomfort, orthopnoea, palpitation, irregular pulse, oedema, shortness of breath on exertion					
**Dermatology*** Rash, itching, infestation, spots, skin lesions/moles, nail issues, changes in pigmentation					
**Eyes, Ears, Nose and Throat*** Red eye, visual disturbance, acute loss of vision, eye discharge, eye injury, foreign body, swollen eyelid, dizziness, vertigo, otalgia, otorrhoea, sinus pain, nasal pain, nasal obstruction, mouth pain, neck swelling, sore throat, throat swelling, tinnitus, hearing loss, voice changes					
**Emergency presentations*** Cardiac arrest, catastrophic haemorrhage, shock, respiratory distress, cardiovascular emergency, anaphylaxis, angioedema, allergic reaction, collapse, seizure, non-blanching rash, overdose/poisoning, suspected DKA, meningism, limp child					
**Gastrointestinal system*** Difficulty swallowing, poor appetite, excessive thirst, abdominal pain, abdominal distention, abdominal mass/swelling, constipation, diarrhoea, change in bowel habit, nausea/vomiting, hematemesis, indigestion, rectal bleeding, abdominal blood results (eg. deranged liver enzymes, LFTs, anaemia), high risk behaviours and concerns, stoma issues					

For peer review only

SURVEY QUESTIONNAIRE

**Genitourinary System*** Loin pain, groin pain, haematuria, urinary symptoms, kidney disease, recurrent infection, penile pain, testicular pain/swelling, inability to pass urine, profuse vaginal bleeding, acute groin swelling/pain					
**General presentations*** Breast symptoms, tired all the time, generalised aches and pains, lymphadenopathy, sleep issues, fever, substance/alcohol misuse, overdose/poisoning, vulnerable adult, family/carer concern, genetic predisposition, presentations in patients with a learning disability; review of blood test results					
**Sexual health assessment*** Genital rashes/irritation, urinary symptoms, penile pain, penile discharge, acute groin swelling/pain, pelvic pain/mass, contraception					
**Medication Review*** Adverse side effects, ineffective medication, poor compliance, overuse of medication, misuse of medication, issues with polypharmacy, abnormal blood test results, higher risk groups (risk reduction medications)					
**Mental Health*** Suicidal ideation, self-harm, acute anxiety, stress, panic, post-natal mental health issues, visual/auditory hallucinations, paranoia, bereavement, substance misuse					
**Musculoskeletal system*** Pain, swelling, redness, stiffness, difficulty with movement, minor injury					
**Neurological system*** Altered level of consciousness, fits faints and funny turns, dizziness, altered power tone sensitivity, paraesthesia, altered level of consciousness,					

For peer review only

SURVEY QUESTIONNAIRE

weakness, altered gait, facial palsy, tremor, speech changes, headache, head injury, memory problems, confusion					
**Paediatrics*** Vulnerable child, rashes, pyrexia of unknown origin, crying baby, otalgia/otorrhoea, eye injury, red eye/discharge, cough/wheeze/stridor/respiratory distress/nasal symptoms, sore throat, vomiting, diarrhoea, acute bowel symptoms, abdominal pain, constipation, musculoskeletal symptoms, minor injury					
**Pain*** Acute pain, worsening pain, change in type of pain					
**Palliative and end of life care*** Symptom management, discussions about advanced care planning, verification of death					
**Pregnancy related conditions**					
**Non-pregnancy related conditions in pregnant people**					
**Respiratory System*** Shortness of breath, breathing difficulties, pain on breathing, cough (including haemoptysis), wheeze, pallor, cyanosis, suspected or recurrent infection, acute covid-19, post covid-19 syndrome					

For peer review only

SURVEY QUESTIONNAIRE

Section 4.
The purpose of this section is to understand the type of clinical investigations and procedural skills that make up your role as a paramedic in primary care. For each of the following activities, please order the regularity that you undertake these in your role in primary care

	Not at all	Sometimes	Regularly	Very regularly	All the time
Abdominal examination – including inspection, auscultation, percussion & palpation					
Assessment for lymphadenopathy					
Blood pressure					
Blood sugar					
Blood ketones					
Blood tests – FBC, ESR, iron studies, TFT, HbA1c, LFT, U&Es, haematinics, PSA, ACR, B12, drug levels, calcium, CRP, clotting factors, vitamin D, rheumatoid factor, anti CCP, urate, D-dimer, INR, TnT, autoimmune antibodies, TFT, lipid profile, testosterone, SHBG, free androgen index, FSH/LH +/- prolactin, CA125, CA19-9, CRP, coeliac screen, amylase, hepatitis, HIV, glandular fever screen/monospot, BNP/NT-proBNP					
Cardiovascular examination – including inspection, auscultation & palpation, Jugular venous pressure					
Core paramedic skills: Emergency procedures for seeking assistance and calling ambulance; provide basic life support (CPR, defibrillator); administration of nebulised therapies; management of anaphylaxis; management of suspected meningitis; management of seizures; management of					

For peer review only

SURVEY QUESTIONNAIRE

suspected MI; administration of oxygen					
Digital rectal examination					
Interpretation of injection fraction from echocardiogram (Echo)					
Electrocardiograph (ECG)					
Eye examination including inspection and visual acuity, fundoscopy and pupils, fluorescein, local anaesthetic					
Respiratory clinic - FeNO testing Spirometry					
Request for Imaging (eg. X-ray, CT, MRI, Ultrasound of organs, MSK and soft tissue, pelvic transvaginal and testicular)					
Interpretation for imaging					
Joint Injections					
Prescribing					
Mental health examination – Person Health Questionnaire (PHQ9); Generalised Anxiety Disorder Questionnaire (GAD7); Edinburgh Post					
Mid-stream urine culture					
Minor Surgery					
Mini mental state examination (MMSE), GPCOG and 6CIT, 480					
Musculoskeletal examination – including spine, neck, shoulders, elbows, wrists, hands, fingers, hips, pelvis, knee, ankle, feet and toes using Look/Feel/Move principles					
Neurological examination – including inspection, palpation, reflexes, sensation, power, tone, strength, pupils and nystagmus, cranial nerve and cerebellar testing					
Otoscopy					
Oxygen saturations					
Peak Expiratory Flow Rate					
Phlebotomy					

For peer review only

SURVEY QUESTIONNAIRE

Pulse rate, rhythm, volume and character					
Referral to specialism or specialist services					
Respiratory examination – including inspection, auscultation, percussion and palpation					
Respiratory rate					
Skin and/or nail scrapings/samples					
Smear					
Spirometry					
Sputum sample					
Stool sample – culture and sensitivity, faecal calprotectin, helicobacter-pylori testing, FIT testing or FOB					
Swabs					
Temperature					
Throat examination					
Urinalysis and HCG					
Vaginal examination (PV)					
Are there any clinical investigations and procedural skills that you undertake that we have not listed here?		Let me type (answer box)			

For peer review only

SURVEY QUESTIONNAIRE

**Section 5.** The purpose of this section is to understand your perspectives of your role working in primary care. You are welcome to write your answers, or bullet point them – whichever works best for you	
Why did you choose to work in primary care?	
Compared to your previous role, how do you find your current role in primary care?	Prompt: What are the challenges? What are the differences?
Do you experience conflict in your role?	Yes – go to next Q<br>No – skip
What type of conflict occurs?	
Do you receive clinical supervision in your role?	Yes – go to next Q<br>No – skip
To what extent does the clinical supervision/support you receive meet your needs?	
Do you feel patients are satisfied following a consultation with you?	Yes – go to next Q<br>No - go to next Q (negative)
Why do you feel patients are (not) satisfied following a consultation with you?	
How does Health Education England's Roadmap to Practice in primary care impact you in your role?	
What frustrations do you have about your role?	
How does your role as a paramedic in primary care make a difference to:<br>- Patients<br>- Other members of the primary care team<br>- The profession	

For peer review only

Supplemental Table A. Relationship between core capabilities of primary care and demographics of paramedics

			Prescribing Status	Clinical Supervision	Length of time registered as a paramedic	Length of time in primary care	Salary	Hours worked	Job Title	Level of Education
Spearman's rho	Communication and consultations	Correlation Coefficient	-0.102	-0.042	.150**	0.051	.127*	-0.050	.138*	.112*
		Sig. (2-tailed)	0.059	0.436	0.006	0.349	0.019	0.356	0.011	0.038
		N	341	341	341	341	341	341	341	341
	Practising holistically to personalise care and promote public and person	Correlation Coefficient	-.162**	-0.015	-0.016	0.066	.159**	-0.076	.123*	.115*
		Sig. (2-tailed)	0.003	0.779	0.767	0.226	0.003	0.164	0.023	0.034
		N	341	341	341	341	341	341	341	341
	Working with colleagues in primary care	Correlation Coefficient	-.107*	-.128*	0.069	-0.015	0.077	-.189**	0.066	0.048
		Sig. (2-tailed)	0.048	0.018	0.204	0.783	0.156	0.000	0.225	0.375
		N	341	341	341	341	341	341	341	341
	Maintaining an ethical approach and fitness to practice	Correlation Coefficient	-0.055	-0.095	.139*	0.026	0.078	-0.083	0.095	0.013
		Sig. (2-tailed)	0.312	0.081	0.010	0.629	0.153	0.125	0.080	0.812
		N	341	341	341	341	341	341	341	341
	Information gathering and interpretation	Correlation Coefficient	-.123*	-0.059	0.062	0.066	.177**	-0.024	.219**	.125*
		Sig. (2-tailed)	0.023	0.280	0.251	0.225	0.001	0.658	0.000	0.021
		N	341	341	341	341	341	341	341	341
	Clinical Examination and procedural skills	Correlation Coefficient	-.119*	-0.005	.142**	0.106	0.102	-0.079	0.079	0.081
		Sig. (2-tailed)	0.028	0.924	0.009	0.051	0.059	0.145	0.145	0.137
		N	341	341	341	341	341	341	341	341
	Making a diagnosis	Correlation Coefficient	-.284**	0.016	0.092	.251**	.231**	-0.081	.174**	.196**
		Sig. (2-tailed)	0.000	0.763	0.090	0.000	0.000	0.134	0.001	0.000
		N	341	341	341	341	341	341	341	341
	Managing medical and clinical complexity	Correlation Coefficient	-.298**	-0.047	0.017	.170**	.236**	-.145**	.209**	.156**
		Sig. (2-tailed)	0.000	0.391	0.750	0.002	0.000	0.007	0.000	0.004
		N	341	341	341	341	341	341	341	341
	Independent prescribing, medicines, and supply of pharmacotherapy	Correlation Coefficient	-.757**	-0.044	0.097	.357**	.481**	-0.091	.334**	.403**
		Sig. (2-tailed)	0.000	0.416	0.075	0.000	0.000	0.092	0.000	0.000
		N	341	341	341	341	341	341	341	341
	Leadership and Management	Correlation Coefficient	-.331**	0.016	0.045	.302**	.255**	-.213**	.158**	.175**
		Sig. (2-tailed)	0.000	0.774	0.403	0.000	0.000	0.000	0.003	0.001
		N	341	341	341	341	341	341	341	341
	Education and Development	Correlation Coefficient	-.143**	-0.008	0.003	.143**	0.106	-0.095	0.105	0.087
		Sig. (2-tailed)	0.008	0.888	0.956	0.008	0.051	0.080	0.052	0.110
		N	341	341	341	341	341	341	341	341
	Research and evidence-based practice	Correlation Coefficient	-.114*	0.000	0.005	0.033	.133*	-0.088	0.095	0.082
		Sig. (2-tailed)	0.035	0.997	0.927	0.546	0.014	0.104	0.079	0.131
		N	341	341	341	341	341	341	341	341

**. Correlation is significant at the 0.01 level (2-tailed).
*. Correlation is significant at the 0.05 level (2-tailed).

For peer review only

Supplemental Table B. Correlations between clinical presentations and demographics of paramedics

			Length of time registered as a paramedic	Length of time in primary care	Hours worked	Level of Education	Job Title	Prescribing Status	Clinical Supervision
Spearman's rho	Breast symptoms	Correlation Coefficient	-0.018	0.087	-0.038	.169**	.138*	.191**	-0.024
		Sig. (2-tailed)	0.735	0.109	0.483	0.002	0.011	0.000	0.664
		N	341	341	341	341	341	341	341
	Tired all the time	Correlation Coefficient	-0.036	0.029	0.077	0.105	0.096	.160**	.165**
		Sig. (2-tailed)	0.513	0.590	0.154	0.053	0.077	0.003	0.002
		N	341	341	341	341	341	341	341
	Generalised aches and pains	Correlation Coefficient	-0.037	-0.001	0.052	0.064	0.069	0.081	.121*
		Sig. (2-tailed)	0.493	0.988	0.335	0.238	0.203	0.134	0.026
		N	341	341	341	341	341	341	341
	Lymphadenopathy	Correlation Coefficient	0.054	0.057	.136*	.149**	.125*	.228**	.138*
		Sig. (2-tailed)	0.318	0.298	0.012	0.006	0.021	0.000	0.011
		N	341	341	341	341	341	341	341
	Sleep issues	Correlation Coefficient	-0.104	-0.039	0.060	0.080	0.040	.107*	0.074
		Sig. (2-tailed)	0.056	0.472	0.267	0.140	0.457	0.048	0.171
		N	341	341	341	341	341	341	341
	Fever	Correlation Coefficient	0.000	0.044	0.070	0.101	.150**	.127*	0.038
		Sig. (2-tailed)	0.999	0.415	0.200	0.063	0.006	0.019	0.486
		N	341	341	341	341	341	341	341
	Substance/alcohol misuse	Correlation Coefficient	-0.018	.175**	.127*	0.092	.115*	0.092	0.014
		Sig. (2-tailed)	0.743	0.001	0.019	0.091	0.034	0.090	0.791
		N	341	341	341	341	341	341	341
	Overdose/poisoning	Correlation Coefficient	-0.073	.160**	.254**	0.057	0.101	0.074	-0.048
		Sig. (2-tailed)	0.181	0.003	0.000	0.290	0.061	0.174	0.377
		N	341	341	341	341	341	341	341
	Vulnerable adult, family/carer concern	Correlation Coefficient	0.016	0.054	.216**	-0.069	-0.033	-0.013	0.072
		Sig. (2-tailed)	0.765	0.320	0.000	0.205	0.545	0.807	0.185
		N	341	341	341	341	341	341	341
	Genetic predisposition	Correlation Coefficient	0.049	0.069	0.029	0.043	0.036	0.027	0.029
		Sig. (2-tailed)	0.365	0.204	0.590	0.424	0.504	0.617	0.593
		N	341	341	341	341	341	341	341
	Presentations in patients with a learning disability	Correlation Coefficient	0.097	.117*	.178**	0.029	0.075	0.063	0.024
		Sig. (2-tailed)	0.075	0.031	0.001	0.588	0.166	0.249	0.653
		N	341	341	341	341	341	341	341
	Review of blood test results	Correlation Coefficient	-0.001	0.087	0.094	.199**	.201**	.282**	.144**
		Sig. (2-tailed)	0.982	0.109	0.082	0.000	0.000	0.000	0.008
		N	341	341	341	341	341	341	341
	Genital rashes/irritation	Correlation Coefficient	0.022	.145**	0.094	.188**	.239**	.332**	-0.024
		Sig. (2-tailed)	0.688	0.007	0.082	0.000	0.000	0.000	0.662
		N	341	341	341	341	341	341	341
	Urinary symptoms	Correlation Coefficient	-0.003	0.013	0.036	0.060	.174**	.134*	0.042
		Sig. (2-tailed)	0.949	0.804	0.513	0.273	0.001	0.013	0.439
		N	341	341	341	341	341	341	341
	Penile pain	Correlation Coefficient	-0.027	.170**	.147**	.234**	.200**	.331**	0.039
		Sig. (2-tailed)	0.614	0.002	0.007	0.000	0.000	0.000	0.469
		N	341	341	341	341	341	341	341
	Penile discharge	Correlation Coefficient	0.037	.158**	.133*	.195**	.137*	.293**	0.032
		Sig. (2-tailed)	0.499	0.003	0.014	0.000	0.011	0.000	0.560
		N	341	341	341	341	341	341	341
	Acute groin swelling/pain	Correlation Coefficient	-0.017	.184**	.140**	.206**	.202**	.276**	0.038
		Sig. (2-tailed)	0.754	0.001	0.010	0.000	0.000	0.000	0.487
		N	341	341	341	341	341	341	341
	Pelvic pain/mass	Correlation Coefficient	0.050	.164**	.136*	.169**	.167**	.242**	-0.024
		Sig. (2-tailed)	0.361	0.002	0.012	0.002	0.002	0.000	0.665
		N	341	341	341	341	341	341	341
	Adverse side effects of medication	Correlation Coefficient	0.046	.222**	0.080	.131*	.118*	.255**	0.026
		Sig. (2-tailed)	0.402	0.000	0.142	0.016	0.029	0.000	0.626
		N	341	341	341	341	341	341	341
	Ineffective medication	Correlation Coefficient	0.044	.149**	0.085	.139*	.145**	.274**	0.015
		Sig. (2-tailed)	0.420	0.006	0.117	0.010	0.007	0.000	0.776
		N	341	341	341	341	341	341	341
	Poor compliance with medication	Correlation Coefficient	0.103	.144**	0.105	.110*	.135*	.191**	0.071
		Sig. (2-tailed)	0.058	0.008	0.052	0.042	0.012	0.000	0.188
		N	341	341	341	341	341	341	341
	Overuse of medication	Correlation Coefficient	0.099	.197**	0.083	.122*	.230**	.243**	0.041
		Sig. (2-tailed)	0.068	0.000	0.127	0.024	0.000	0.000	0.451
		N	341	341	341	341	341	341	341
	Misuse of medication	Correlation Coefficient	0.101	.188**	.130*	.128*	.156**	.203**	-0.005
		Sig. (2-tailed)	0.063	0.000	0.017	0.018	0.004	0.000	0.929
		N	341	341	341	341	341	341	341
	Issues with polypharmacy	Correlation Coefficient	.120*	.195**	.145**	.163**	.187**	.302**	0.038
		Sig. (2-tailed)	0.026	0.000	0.007	0.002	0.001	0.000	0.488
		N	341	341	341	341	341	341	341

For peer review only

Abnormal blood test results	Correlation Coefficient	0.055	0.095	0.098	.186**	.194**	.264**	0.105
	Sig. (2-tailed)	0.312	0.080	0.072	0.001	0.000	0.000	0.052
	N	341	341	341	341	341	341	341
Higher risk groups (risk reduction medications)	Correlation Coefficient	0.078	.206**	.116*	.145**	.159**	.235**	0.044
	Sig. (2-tailed)	0.152	0.000	0.033	0.007	0.003	0.000	0.416
	N	341	341	341	341	341	341	341
Suicidal ideation	Correlation Coefficient	0.032	.158**	0.070	.122*	.226**	0.095	0.012
	Sig. (2-tailed)	0.551	0.003	0.200	0.024	0.000	0.081	0.823
	N	341	341	341	341	341	341	341
Self-harm	Correlation Coefficient	0.048	.179**	0.104	0.101	.201**	0.096	-0.048
	Sig. (2-tailed)	0.373	0.001	0.056	0.062	0.000	0.075	0.372
	N	341	341	341	341	341	341	341
Acute anxiety	Correlation Coefficient	0.079	0.072	0.076	.141**	.212**	.148**	0.022
	Sig. (2-tailed)	0.143	0.188	0.161	0.009	0.000	0.006	0.679
	N	341	341	341	341	341	341	341
Stress	Correlation Coefficient	0.064	0.094	0.016	.164**	.194**	.154**	0.029
	Sig. (2-tailed)	0.237	0.083	0.770	0.002	0.000	0.004	0.591
	N	341	341	341	341	341	341	341
Panic	Correlation Coefficient	0.056	.131*	0.058	.159**	.245**	.180**	0.006
	Sig. (2-tailed)	0.301	0.015	0.284	0.003	0.000	0.001	0.914
	N	341	341	341	341	341	341	341
Post-natal mental health issues	Correlation Coefficient	0.002	0.027	-0.019	.151**	.123*	0.035	-0.069
	Sig. (2-tailed)	0.971	0.624	0.727	0.005	0.023	0.523	0.205
	N	341	341	341	341	341	341	341
Visual/auditory hallucinations	Correlation Coefficient	0.064	0.100	.181**	0.046	0.063	.116*	-0.034
	Sig. (2-tailed)	0.236	0.064	0.001	0.400	0.245	0.032	0.534
	N	341	341	341	341	341	341	341
Paranoia	Correlation Coefficient	0.090	.134*	.144**	0.069	0.102	.130*	-0.085
	Sig. (2-tailed)	0.096	0.013	0.008	0.206	0.060	0.016	0.115
	N	341	341	341	341	341	341	341
Bereavement	Correlation Coefficient	0.057	0.097	0.076	.135*	.108*	.176**	0.027
	Sig. (2-tailed)	0.294	0.075	0.162	0.013	0.045	0.001	0.619
	N	341	341	341	341	341	341	341
Substance misuse	Correlation Coefficient	0.062	.145**	0.100	.112*	.231**	.117*	-0.062
	Sig. (2-tailed)	0.253	0.007	0.065	0.039	0.000	0.031	0.255
	N	341	341	341	341	341	341	341
Pain	Correlation Coefficient	0.070	0.019	0.051	0.063	.153**	0.039	0.057
	Sig. (2-tailed)	0.198	0.725	0.346	0.249	0.005	0.472	0.296
	N	341	341	341	341	341	341	341
Swelling	Correlation Coefficient	0.027	0.093	.133*	0.023	.152**	0.077	0.033
	Sig. (2-tailed)	0.617	0.087	0.014	0.679	0.005	0.155	0.540
	N	341	341	341	341	341	341	341
Redness	Correlation Coefficient	0.031	0.092	.164**	0.006	.146**	0.093	0.051
	Sig. (2-tailed)	0.569	0.090	0.002	0.907	0.007	0.086	0.350
	N	341	341	341	341	341	341	341
Stiffness	Correlation Coefficient	0.040	0.062	0.100	0.042	.128*	.112*	0.064
	Sig. (2-tailed)	0.456	0.253	0.064	0.442	0.018	0.039	0.240
	N	341	341	341	341	341	341	341
Difficulty with movement	Correlation Coefficient	0.036	0.023	0.104	0.023	.113*	0.072	0.054
	Sig. (2-tailed)	0.508	0.669	0.055	0.668	0.037	0.186	0.317
	N	341	341	341	341	341	341	341
Minor injury	Correlation Coefficient	-0.049	0.040	0.082	-0.020	0.061	-0.023	-0.007
	Sig. (2-tailed)	0.369	0.467	0.129	0.709	0.265	0.669	0.902
	N	341	341	341	341	341	341	341
Altered level of consciousness	Correlation Coefficient	0.081	.156**	.170**	-0.012	0.054	0.036	0.024
	Sig. (2-tailed)	0.136	0.004	0.002	0.820	0.320	0.508	0.656
	N	341	341	341	341	341	341	341
Fits faints and funny turns	Correlation Coefficient	-0.016	.118*	.110*	0.069	.118*	0.060	0.096
	Sig. (2-tailed)	0.762	0.029	0.043	0.204	0.030	0.268	0.078
	N	341	341	341	341	341	341	341
Dizziness	Correlation Coefficient	0.004	0.042	0.061	.144**	.125*	0.092	0.101
	Sig. (2-tailed)	0.937	0.436	0.258	0.008	0.021	0.090	0.061
	N	341	341	341	341	341	341	341
Altered power, tone or sensitivity	Correlation Coefficient	0.044	0.101	.120*	0.096	.119*	0.065	0.039
	Sig. (2-tailed)	0.423	0.062	0.027	0.076	0.028	0.231	0.473
	N	341	341	341	341	341	341	341
Paraesthesia	Correlation Coefficient	0.057	.211**	0.081	.139**	.189**	.199**	0.017
	Sig. (2-tailed)	0.298	0.000	0.138	0.010	0.000	0.000	0.758
	N	341	341	341	341	341	341	341
Altered level of consciousness	Correlation Coefficient	0.068	.149**	.176**	-0.022	0.039	0.064	0.013
	Sig. (2-tailed)	0.209	0.006	0.001	0.687	0.472	0.239	0.812
	N	341	341	341	341	341	341	341
Weakness	Correlation Coefficient	0.025	0.050	.133*	0.032	0.068	0.030	0.104
	Sig. (2-tailed)	0.650	0.361	0.014	0.550	0.210	0.581	0.054
	N	341	341	341	341	341	341	341
Altered gait	Correlation Coefficient	0.095	0.075	.165**	0.019	0.018	0.053	0.062

For peer review only

	Sig. (2-tailed)	0.081	0.169	0.002	0.729	0.743	0.326	0.251
	N	341	341	341	341	341	341	341
Facial palsy	Correlation Coefficient	0.086	.185**	.131*	0.051	0.080	.184**	0.017
	Sig. (2-tailed)	0.112	0.001	0.015	0.347	0.138	0.001	0.754
	N	341	341	341	341	341	341	341
Tremor	Correlation Coefficient	0.013	.134*	.127*	0.042	0.031	0.099	0.039
	Sig. (2-tailed)	0.813	0.014	0.019	0.440	0.568	0.067	0.474
	N	341	341	341	341	341	341	341
Speech changes	Correlation Coefficient	0.049	.184**	.224**	0.014	0.011	0.097	0.031
	Sig. (2-tailed)	0.367	0.001	0.000	0.797	0.841	0.073	0.567
	N	341	341	341	341	341	341	341
Headache	Correlation Coefficient	-0.046	0.025	0.046	.172**	.195**	.153**	0.103
	Sig. (2-tailed)	0.396	0.643	0.396	0.001	0.000	0.005	0.057
	N	341	341	341	341	341	341	341
Head injury	Correlation Coefficient	-0.053	0.068	0.029	0.037	0.092	0.001	-0.028
	Sig. (2-tailed)	0.330	0.207	0.593	0.498	0.088	0.989	0.605
	N	341	341	341	341	341	341	341
Memory problems	Correlation Coefficient	0.075	0.084	.178**	0.032	-0.028	.130*	0.090
	Sig. (2-tailed)	0.166	0.120	0.001	0.553	0.607	0.016	0.098
	N	341	341	341	341	341	341	341
Confusion	Correlation Coefficient	0.070	.116*	.229**	0.017	0.012	.128*	0.071
	Sig. (2-tailed)	0.196	0.032	0.000	0.753	0.827	0.018	0.190
	N	341	341	341	341	341	341	341
Vulnerable child	Correlation Coefficient	0.022	.175**	0.103	.159**	.200**	.196**	-0.059
	Sig. (2-tailed)	0.686	0.001	0.057	0.003	0.000	0.000	0.279
	N	341	341	341	341	341	341	341
Rashes	Correlation Coefficient	-0.007	.142**	-0.039	.208**	.261**	.238**	0.023
	Sig. (2-tailed)	0.904	0.009	0.472	0.000	0.000	0.000	0.671
	N	341	341	341	341	341	341	341
Pyrexia of unknown origin	Correlation Coefficient	-0.048	0.090	-0.063	.225**	.265**	.145**	0.004
	Sig. (2-tailed)	0.372	0.097	0.248	0.000	0.000	0.007	0.947
	N	341	341	341	341	341	341	341
Crying baby	Correlation Coefficient	-0.069	.149**	-0.048	.214**	.296**	.192**	-0.025
	Sig. (2-tailed)	0.201	0.006	0.372	0.000	0.000	0.000	0.639
	N	341	341	341	341	341	341	341
Otalgia/otorrhoea	Correlation Coefficient	-0.010	.139*	-0.024	.190**	.273**	.223**	0.070
	Sig. (2-tailed)	0.851	0.010	0.663	0.000	0.000	0.000	0.196
	N	341	341	341	341	341	341	341
Eye injury	Correlation Coefficient	-0.042	.197**	0.061	.117*	.116*	.146**	0.003
	Sig. (2-tailed)	0.434	0.000	0.260	0.030	0.032	0.007	0.961
	N	341	341	341	341	341	341	341
Red eye/discharge	Correlation Coefficient	0.026	.212**	0.035	.193**	.222**	.253**	0.045
	Sig. (2-tailed)	0.628	0.000	0.517	0.000	0.000	0.000	0.405
	N	341	341	341	341	341	341	341
Cough/wheeze/stridor/respiratory distress/nasal symptoms	Correlation Coefficient	0.005	.109*	-0.032	.203**	.249**	.148**	0.043
	Sig. (2-tailed)	0.923	0.045	0.557	0.000	0.000	0.006	0.433
	N	341	341	341	341	341	341	341
Sore throat	Correlation Coefficient	0.003	0.099	-0.020	.224**	.270**	.185**	0.053
	Sig. (2-tailed)	0.962	0.067	0.713	0.000	0.000	0.001	0.327
	N	341	341	341	341	341	341	341
Vomiting	Correlation Coefficient	-0.053	.149**	0.008	.224**	.262**	.225**	0.066
	Sig. (2-tailed)	0.333	0.006	0.885	0.000	0.000	0.000	0.227
	N	341	341	341	341	341	341	341
Diarrhoea	Correlation Coefficient	-0.046	.149**	0.009	.237**	.246**	.230**	0.059
	Sig. (2-tailed)	0.393	0.006	0.866	0.000	0.000	0.000	0.280
	N	341	341	341	341	341	341	341
Acute bowel symptoms	Correlation Coefficient	-0.058	.144**	0.014	.217**	.254**	.234**	0.040
	Sig. (2-tailed)	0.285	0.008	0.800	0.000	0.000	0.000	0.461
	N	341	341	341	341	341	341	341
Abdominal pain	Correlation Coefficient	0.013	.171**	-0.002	.249**	.293**	.260**	0.053
	Sig. (2-tailed)	0.811	0.002	0.977	0.000	0.000	0.000	0.334
	N	341	341	341	341	341	341	341
Constipation	Correlation Coefficient	0.001	.134*	0.052	.248**	.266**	.294**	0.049
	Sig. (2-tailed)	0.987	0.013	0.335	0.000	0.000	0.000	0.371
	N	341	341	341	341	341	341	341
Musculoskeletal symptoms	Correlation Coefficient	0.015	.173**	-0.024	.158**	.247**	.167**	-0.031
	Sig. (2-tailed)	0.778	0.001	0.660	0.003	0.000	0.002	0.572
	N	341	341	341	341	341	341	341
Minor injury	Correlation Coefficient	-0.039	.190**	-0.030	0.081	.155**	0.092	-0.074
	Sig. (2-tailed)	0.472	0.000	0.583	0.137	0.004	0.090	0.174
	N	341	341	341	341	341	341	341
Acute pain	Correlation Coefficient	0.053	0.096	0.071	.127*	.160**	.122*	0.100
	Sig. (2-tailed)	0.326	0.076	0.190	0.019	0.003	0.024	0.066
	N	341	341	341	341	341	341	341
Worsening pain	Correlation Coefficient	0.046	0.035	0.079	0.050	.140**	0.090	0.052

For peer review only

	Sig. (2-tailed)	0.398	0.523	0.144	0.359	0.010	0.097	0.337
	N	341	341	341	341	341	341	341
Change in type of pain	Correlation Coefficient	0.048	0.079	0.079	0.017	.121*	.133*	0.028
	Sig. (2-tailed)	0.373	0.148	0.145	0.750	0.026	0.014	0.606
	N	341	341	341	341	341	341	341
Symptom management	Correlation Coefficient	0.051	0.089	.205**	-0.029	-0.046	0.044	0.056
	Sig. (2-tailed)	0.349	0.103	0.000	0.593	0.399	0.418	0.300
	N	341	341	341	341	341	341	341
Discussions about advanced care planning	Correlation Coefficient	0.051	.168**	.205**	-0.043	-0.093	0.071	0.047
	Sig. (2-tailed)	0.349	0.002	0.000	0.433	0.088	0.190	0.382
	N	341	341	341	341	341	341	341
Shortness of breath	Correlation Coefficient	-0.011	0.067	.126*	0.076	0.082	.107*	0.064
	Sig. (2-tailed)	0.833	0.218	0.020	0.162	0.130	0.048	0.241
	N	341	341	341	341	341	341	341
Breathing difficulties	Correlation Coefficient	0.023	0.105	.111*	0.097	.114*	.129*	0.069
	Sig. (2-tailed)	0.678	0.052	0.041	0.074	0.036	0.017	0.202
	N	341	341	341	341	341	341	341
Pain on breathing	Correlation Coefficient	-0.009	0.088	0.104	.107*	.118*	0.106	.107*
	Sig. (2-tailed)	0.872	0.105	0.055	0.048	0.030	0.051	0.047
	N	341	341	341	341	341	341	341
Cough (including haemoptysis)	Correlation Coefficient	-0.005	0.054	.129*	.126*	.133*	.140**	0.090
	Sig. (2-tailed)	0.934	0.321	0.017	0.020	0.014	0.010	0.097
	N	341	341	341	341	341	341	341
Wheeze	Correlation Coefficient	0.046	0.100	.128*	.121*	.126*	.146**	0.086
	Sig. (2-tailed)	0.393	0.066	0.018	0.026	0.020	0.007	0.113
	N	341	341	341	341	341	341	341
Pallor	Correlation Coefficient	0.064	.120*	.174**	0.026	0.060	.108*	-0.038
	Sig. (2-tailed)	0.242	0.026	0.001	0.638	0.272	0.046	0.482
	N	341	341	341	341	341	341	341
Cyanosis	Correlation Coefficient	0.021	.144**	.143**	0.068	0.058	.125*	-0.054
	Sig. (2-tailed)	0.693	0.008	0.008	0.213	0.284	0.021	0.317
	N	341	341	341	341	341	341	341
Suspected or recurrent infection	Correlation Coefficient	0.035	-0.015	0.080	0.100	0.090	0.052	0.077
	Sig. (2-tailed)	0.515	0.789	0.138	0.064	0.097	0.342	0.154
	N	341	341	341	341	341	341	341
Acute Covid-19	Correlation Coefficient	-0.079	0.029	.121*	0.063	.125*	0.069	0.005
	Sig. (2-tailed)	0.148	0.588	0.026	0.248	0.021	0.202	0.931
	N	341	341	341	341	341	341	341
Post Covid-19 syndrome	Correlation Coefficient	-0.062	0.034	0.090	0.072	0.042	0.093	-0.028
	Sig. (2-tailed)	0.252	0.535	0.097	0.186	0.435	0.087	0.603
	N	341	341	341	341	341	341	341

**. Correlation is significant at the 0.01 level (2-tailed).

*. Correlation is significant at the 0.05 level (2-tailed).

For peer review only

Supplemental Table C. Correlations between clinical examinations and demogaphics of paramedics

			Length of time in primary care	Hours worked	Level of Education	Job Title	Prescribing Status	Clinical Supervision
Spearman's rho	Abdominal examination	Correlation Coefficient	0.091	.116*	.113*	.129*	.117*	0.062
		Sig. (2-tailed)	0.094	0.032	0.036	0.017	0.031	0.254
		N	341	341	341	341	341	341
	Assessment for lymphadenopathy	Correlation Coefficient	.148**	0.019	.211**	.186**	.257**	0.062
		Sig. (2-tailed)	0.006	0.730	0.000	0.001	0.000	0.254
		N	341	341	341	341	341	341
	Blood pressure	Correlation Coefficient	0.058	0.094	0.071	0.072	0.017	0.064
		Sig. (2-tailed)	0.284	0.085	0.190	0.183	0.751	0.238
		N	340	340	340	340	340	340
	Cardiovascular examination	Correlation Coefficient	0.065	.113*	0.087	.111*	0.066	0.081
		Sig. (2-tailed)	0.230	0.037	0.109	0.040	0.226	0.138
		N	341	341	341	341	341	341
	Cranial nerve examination	Correlation Coefficient	.139**	0.088	.155**	.166**	.132*	0.048
		Sig. (2-tailed)	0.010	0.106	0.004	0.002	0.015	0.376
		N	341	341	341	341	341	341
	Digital rectal examination	Correlation Coefficient	.195**	.141**	.264**	.217**	.444**	0.007
		Sig. (2-tailed)	0.000	0.009	0.000	0.000	0.000	0.902
		N	340	340	340	340	340	340
	Echocardiogram (Echo)	Correlation Coefficient	0.082	0.065	0.091	0.052	.138*	-0.066
		Sig. (2-tailed)	0.133	0.236	0.096	0.343	0.011	0.228
		N	339	339	339	339	339	339
	Electrocardiograph (ECG)	Correlation Coefficient	.127*	.150**	0.027	0.053	0.055	-0.047
		Sig. (2-tailed)	0.019	0.005	0.616	0.329	0.311	0.388
		N	341	341	341	341	341	341
	Eye examination	Correlation Coefficient	.204**	0.017	.212**	0.105	.255**	-0.036
		Sig. (2-tailed)	0.000	0.753	0.000	0.054	0.000	0.513
		N	339	339	339	339	339	339
	FeNO testing	Correlation Coefficient	0.060	0.015	0.091	0.026	.121*	-.193**
		Sig. (2-tailed)	0.271	0.790	0.094	0.632	0.025	0.000
		N	341	341	341	341	341	341
	Imaging	Correlation Coefficient	.129*	0.084	.127*	.214**	.185**	-0.005
		Sig. (2-tailed)	0.018	0.124	0.019	0.000	0.001	0.924
		N	339	339	339	339	339	339
	Jugular venous pressure	Correlation Coefficient	.124*	0.062	.137*	0.089	.181**	0.077
		Sig. (2-tailed)	0.022	0.256	0.011	0.099	0.001	0.155
		N	341	341	341	341	341	341
	Mental health examination	Correlation Coefficient	.126*	0.037	.129*	0.073	.178**	0.092
		Sig. (2-tailed)	0.020	0.496	0.017	0.177	0.001	0.089
		N	341	341	341	341	341	341
	Mid-stream urine culture	Correlation Coefficient	0.013	.179**	0.093	0.075	.129*	.113*
		Sig. (2-tailed)	0.807	0.001	0.088	0.167	0.017	0.037
		N	341	341	341	341	341	341
	Mini mental state examination (MMSE)	Correlation Coefficient	.128*	.126*	0.081	0.040	0.084	0.094
		Sig. (2-tailed)	0.018	0.020	0.135	0.464	0.120	0.082
		N	341	341	341	341	341	341
	Musculoskeletal examinatio	Correlation Coefficient	.133*	0.086	.112*	.153**	0.073	0.017
		Sig. (2-tailed)	0.014	0.113	0.039	0.005	0.180	0.758
		N	340	340	340	340	340	340
	Neurological examination	Correlation Coefficient	0.067	0.084	.124*	.125*	0.052	0.008
		Sig. (2-tailed)	0.217	0.123	0.022	0.021	0.335	0.887
		N	341	341	341	341	341	341
	Otoscopy	Correlation Coefficient	0.088	0.053	0.088	.162**	.193**	0.058
		Sig. (2-tailed)	0.106	0.332	0.104	0.003	0.000	0.282
		N	341	341	341	341	341	341
	Oxygen saturations	Correlation Coefficient	0.029	0.080	-0.067	0.029	-0.105	0.048
		Sig. (2-tailed)	0.599	0.142	0.217	0.593	0.052	0.372
		N	341	341	341	341	341	341
	Peak Expiratory Flow Rate	Correlation Coefficient	.183**	0.045	0.037	0.036	.123*	-0.006
		Sig. (2-tailed)	0.001	0.407	0.491	0.510	0.023	0.909
		N	340	340	340	340	340	340
	Phlebotomy	Correlation Coefficient	-0.085	.220**	-0.047	-0.105	0.033	0.016
		Sig. (2-tailed)	0.116	0.000	0.385	0.053	0.539	0.762
		N	340	340	340	340	340	340
	Pulse rate, rhythm, volume and character	Correlation Coefficient	0.054	0.057	-0.077	0.044	-0.055	-0.016
		Sig. (2-tailed)	0.320	0.292	0.156	0.422	0.310	0.774
		N	341	341	341	341	341	341
	Referral to specialism or specialist services	Correlation Coefficient	.143**	.168**	.118*	0.061	.190**	0.086
		Sig. (2-tailed)	0.008	0.002	0.029	0.258	0.000	0.111

For peer review only

	N	341	341	341	341	341	341
Respiratory examination	Correlation Coefficient	0.085	0.085	0.038	0.087	0.034	-0.012
	Sig. (2-tailed)	0.116	0.116	0.480	0.109	0.527	0.829
	N	341	341	341	341	341	341
Respiratory rate	Correlation Coefficient	0.018	0.084	-0.058	0.024	-0.063	0.013
	Sig. (2-tailed)	0.742	0.121	0.285	0.666	0.248	0.813
	N	340	340	340	340	340	340
Skin and/or nail scrapings/samples	Correlation Coefficient	.149**	.124*	0.042	0.072	.296**	0.045
	Sig. (2-tailed)	0.006	0.022	0.444	0.183	0.000	0.410
	N	341	341	341	341	341	341
Spirometry	Correlation Coefficient	-0.001	0.004	0.032	0.022	0.046	-0.015
	Sig. (2-tailed)	0.986	0.940	0.561	0.687	0.402	0.778
	N	341	341	341	341	341	341
Sputum sample	Correlation Coefficient	.152**	.209**	.118*	0.084	.290**	.134*
	Sig. (2-tailed)	0.005	0.000	0.030	0.121	0.000	0.013
	N	340	340	340	340	340	340
Stool sample	Correlation Coefficient	0.049	.107*	.142**	.126*	.257**	.212**
	Sig. (2-tailed)	0.363	0.049	0.009	0.021	0.000	0.000
	N	340	340	340	340	340	340
Swabs	Correlation Coefficient	0.089	.211**	0.090	0.082	.277**	.126*
	Sig. (2-tailed)	0.099	0.000	0.097	0.133	0.000	0.020
	N	341	341	341	341	341	341
Temperature	Correlation Coefficient	-0.019	0.067	-0.103	-0.023	-.114*	0.023
	Sig. (2-tailed)	0.733	0.215	0.057	0.672	0.035	0.676
	N	341	341	341	341	341	341
Throat examination	Correlation Coefficient	0.063	0.072	0.082	.153**	0.102	0.071
	Sig. (2-tailed)	0.243	0.183	0.129	0.005	0.061	0.193
	N	341	341	341	341	341	341
Urinalysis	Correlation Coefficient	0.050	0.104	-0.004	0.029	0.033	0.071
	Sig. (2-tailed)	0.356	0.056	0.947	0.592	0.548	0.193
	N	340	340	340	340	340	340

**. Correlation is significant at the 0.01 level (2-tailed).

*. Correlation is significant at the 0.05 level (2-tailed).

For peer review only

Supplemental Table D. Correlations between the request and interpretation of blood tests and demogaphics of paramedics

			Prescribing Status	Clinical Supervision	Length of time registered as a paramedic	Length of time in primary care	Salary	Hours worked	Job Title	Level of Education
Spearman's rho	FBC	Correlation Coefficient	.205**	.176**	0.009	0.015	.267**	.125*	.136*	.167**
		Sig. (2-tailed)	<.001	<.001	0.871	0.789	<.001	0.021	0.012	0.002
		N	341	341	341	341	341	341	341	341
	ESR	Correlation Coefficient	.189**	.158**	0.091	-0.028	.201**	.114*	0.104	.158**
		Sig. (2-tailed)	<.001	0.003	0.094	0.600	<.001	0.036	0.055	0.003
		N	341	341	341	341	341	341	341	341
	Iron studies	Correlation Coefficient	.162**	.139**	-0.017	-0.004	.198**	.115*	0.104	.135*
		Sig. (2-tailed)	0.003	0.010	0.760	0.938	0.000	0.034	0.055	0.013
		N	341	341	341	341	341	341	341	341
	TFT	Correlation Coefficient	.276**	.133*	0.039	0.081	.276**	.138*	.154*	.201**
		Sig. (2-tailed)	<.001	0.014	0.476	0.136	<.001	0.011	0.004	0.000
		N	341	341	341	341	341	341	341	341
	HbA1c	Correlation Coefficient	.202**	.155**	-0.064	0.002	.249**	.142**	.118*	.162**
		Sig. (2-tailed)	0.000	0.004	0.235	0.971	0.000	0.009	0.029	0.003
		N	341	341	341	341	341	341	341	341
	LFT	Correlation Coefficient	.183**	.175**	-0.006	-0.007	.247**	.129*	.137*	.165**
		Sig. (2-tailed)	<.001	<.001	0.911	0.900	<.001	0.017	0.011	0.002
		N	341	341	341	341	341	341	341	341
	U&Es	Correlation Coefficient	.185**	.175**	0.006	-0.007	.261**	.146**	.123*	.152**
		Sig. (2-tailed)	<.001	0.001	0.906	0.898	<.001	0.007	0.024	0.005
		N	341	341	341	341	341	341	341	341
	Haematinics	Correlation Coefficient	.304**	0.080	0.069	0.086	.279**	.201**	.152*	.144**
		Sig. (2-tailed)	<.001	0.140	0.206	0.111	<.001	<.001	0.005	0.008
		N	341	341	341	341	341	341	341	341
	PSA	Correlation Coefficient	.297**	.123*	-0.008	0.002	.293**	.195**	.137*	.205**
		Sig. (2-tailed)	<.001	0.023	0.876	0.977	<.001	<.001	0.011	<.001
		N	341	341	341	341	341	341	341	341
	ACR	Correlation Coefficient	.230**	0.093	-0.021	.116*	.236**	.232**	0.094	.123*
		Sig. (2-tailed)	<.001	0.085	0.705	0.033	<.001	<.001	0.082	0.023
		N	341	341	341	341	341	341	341	341
	B12	Correlation Coefficient	.245**	.125*	-0.005	0.011	.273**	0.100	.158*	.149**
		Sig. (2-tailed)	<.001	0.021	0.922	0.835	<.001	0.066	0.003	0.006
		N	341	341	341	341	341	341	341	341
	Drug levels	Correlation Coefficient	.172**	0.025	0.007	0.037	.164**	.181**I36	0.011	0.095
		Sig. (2-tailed)	<.001	0.641	0.895	0.498	0.002	<.001	0.836	0.079
		N	341	341	341	341	341	341	341	341
	Calcium	Correlation Coefficient	.251**	.130*	0.055	0.025	.214**	.165**	.110*	.166**

For peer review only

	Sig. (2-tailed)	<.001	0.016	0.312	0.643	<.001	0.002	0.043	0.002
	N	341	341	341	341	341	341	341	341
CRP	Correlation Coefficient	.246**	.153**	0.009	0.083	.296**	.189**	0.099	.108*
	Sig. (2-tailed)	<.001	0.005	0.871	0.124	<.001	<.001	0.068	0.047
	N	341	341	341	341	341	341	341	341
Vitamin D	Correlation Coefficient	.183**	.142**	-0.066	-0.027	.192**	0.090	0.095	.147**
	Sig. (2-tailed)	<.001	0.009	0.225	0.623	0.000	0.098	0.078	0.006
	N	341	341	341	341	341	341	341	341
Rheumatoid factor	Correlation Coefficient	.248**	0.082	0.019	0.081	.235**	0.085	.154*	.196**
	Sig. (2-tailed)	<.001	0.130	0.727	0.137	<.001	0.117	0.004	<.001
	N	341	341	341	341	341	341	341	341
Anti CCP	Correlation Coefficient	.248**	0.080	0.035	0.087	.195**	0.047	.147*	.165**
	Sig. (2-tailed)	<.001	0.139	0.514	0.108	<.001	0.389	0.007	0.002
	N	341	341	341	341	341	341	341	341
Urate	Correlation Coefficient	.256**	.141**	0.063	.133*	.288**	.193**	.122*	.167**
	Sig. (2-tailed)	<.001	0.009	0.244	0.014	<.001	<.001	0.025	0.002
	N	341	341	341	341	341	341	341	341
Autoimmune antibodies	Correlation Coefficient	.197**	0.100	-0.025	0.041	.223**	0.084	.129*	.198**
	Sig. (2-tailed)	<.001	0.065	0.649	0.448	<.001	0.120	0.017	<.001
	N	341	341	341	341	341	341	341	341
Lipid profile	Correlation Coefficient	.278**	0.104	-0.003	0.077	.341**	0.071	.158*	.192**
	Sig. (2-tailed)	<.001	0.054	0.957	0.157	<.001	0.192	0.003	0.000
	N	341	341	341	341	341	341	341	341
estosterone	Correlation Coefficient	.134*	0.009	-0.023	0.013	.144**	0.090	0.044	.117*
	Sig. (2-tailed)	0.013	0.869	0.671	0.806	0.008	0.097	0.415	0.030
	N	341	341	341	341	341	341	341	341
SHBG	Correlation Coefficient	.132*	-0.034	0.009	0.020	0.101	0.008	0.027	.155**
	Sig. (2-tailed)	0.014	0.530	0.867	0.707	0.063	0.885	0.623	0.004
	N	341	341	341	341	341	341	341	341
Free androgen index	Correlation Coefficient	0.098	-0.054	-0.044	0.022	0.104	0.019	0.024	.129*
	Sig. (2-tailed)	0.070	0.320	0.415	0.686	0.055	0.732	0.657	0.017
	N	341	341	341	341	341	341	341	341
FSH/LH +/- prolactin	Correlation Coefficient	.161**	0.005	0.018	0.032	.132*	0.071	0.089	.144**
	Sig. (2-tailed)	0.003	0.920	0.746	0.552	0.015	0.189	0.101	0.008
	N	341	341	341	341	341	341	341	341
CA125	Correlation Coefficient	.268**	0.092	0.005	.115*	.257**	.161**	.148*	.186**
	Sig. (2-tailed)	<.001	0.091	0.923	0.034	<.001	0.003	0.006	0.001
	N	341	341	341	341	341	341	341	341
CRP	Correlation Coefficient	.221**	.151**	0.021	0.072	.281**	.178**	0.086	.116*
	Sig. (2-tailed)	<.001	0.005	0.695	0.188	<.001	0.001	0.111	0.032
	N	341	341	341	341	341	341	341	341

For peer review only

Coeliac screen	Correlation Coefficient	.305**	0.077	-0.054	.126*	.326**	0.086	.146*	.197**
	Sig. (2-tailed)	<.001	0.153	0.322	0.020	<.001	0.114	0.007	0.000
	N	341	341	341	341	341	341	341	341
Amylase	Correlation Coefficient	.306**	0.077	0.059	.115*	.266**	.118*	.150*	.198**
	Sig. (2-tailed)	<.001	0.156	0.274	0.033	<.001	0.030	0.005	<.001
	N	341	341	341	341	341	341	341	341
Hepatitis	Correlation Coefficient	.193**	0.076	-0.015	.120*	.234**	0.087	.127*	.156**
	Sig. (2-tailed)	0.000	0.164	0.779	0.027	<.001	0.107	0.019	0.004
	N	341	341	341	341	341	341	341	341
HIV	Correlation Coefficient	.192**	0.045	-0.034	.154**	.208**	0.069	.137*	.136*
	Sig. (2-tailed)	<.001	0.407	0.526	0.004	<.001	0.205	0.011	0.012
	N	341	341	341	341	341	341	341	341
Glandular fever screen	Correlation Coefficient	.342**	0.061	.123*	.223**	.324**	0.104	.168*	.204**
	Sig. (2-tailed)	0.000	0.264	0.023	<.001	<.001	0.056	0.002	0.000
	N	341	341	341	341	341	341	341	341
BNP/NT-proBNP	Correlation Coefficient	0.278	.153**	0.022	.109*	.297**	.228**	0.095	.134*
	Sig. (2-tailed)	<.001	0.005	0.687	0.044	<.001	<.001	0.081	0.013
	N	341	341	341	341	341	341	341	341

**. Correlation is significant at the 0.01 level (2-tailed).

*. Correlation is significant at the 0.05 level (2-tailed).

For peer review only

Supplemental Table E. Associations between prescribing status and clinical skills assessments in primary care

Clinical Assessments	Value	df	Asymptotic Significance (2-sided)
Lymphadenopathy	28.75	4	<.001
Digital rectal examination	68.059	4	215
Eye examination	24.228	4	<.001
Imaging	15.861	4	0.003
Mental health examination	14.886	4	0.005
Otoscopy	21.312	4	<.001
Referrals to specialism or specialist services	14.886	4	0.003
Skin samples	37.529	4	<.001
Sputum samples	31.322	4	<.001
Stool samples	24.25	4	<.001
Swabs	32.213	4	<.001

For peer review only

STROBE Statement—Checklist of items that should be included in reports of ***cross-sectional studies***

	Item No	Recommendation	Page No
**Title and abstract**	1	(*a*) Indicate the study's design with a commonly used term in the title or the abstract	1,3
		(*b*) Provide in the abstract an informative and balanced summary of what was done and what was found	3
**Introduction**			
Background/rationale	2	Explain the scientific background and rationale for the investigation being reported	5,6
Objectives	3	State specific objectives, including any prespecified hypotheses	6
**Methods**			
Study design	4	Present key elements of study design early in the paper	4,5
Setting	5	Describe the setting, locations, and relevant dates, including periods of recruitment, exposure, follow-up, and data collection	6,7
Participants	6	(*a*) Give the eligibility criteria, and the sources and methods of selection of participants	7; Supp 1
Variables	7	Clearly define all outcomes, exposures, predictors, potential confounders, and effect modifiers. Give diagnostic criteria, if applicable	4, 15,16
Data sources/ measurement	8*	For each variable of interest, give sources of data and details of methods of assessment (measurement). Describe comparability of assessment methods if there is more than one group	7
Bias	9	Describe any efforts to address potential sources of bias	18,19
Study size	10	Explain how the study size was arrived at	7
3Quantitative variables	11	Explain how quantitative variables were handled in the analyses. If applicable, describe which groupings were chosen and why	7
Statistical methods	12	(*a*) Describe all statistical methods, including those used to control for confounding	7
		(*b*) Describe any methods used to examine subgroups and interactions	NA
		(*c*) Explain how missing data were addressed	NA
		(*d*) If applicable, describe analytical methods taking account of sampling strategy	7
		(*e*) Describe any sensitivity analyses	NA
**Results**			
Participants	13*	(a) Report numbers of individuals at each stage of study—eg numbers potentially eligible, examined for eligibility, confirmed eligible, included in the study, completing follow-up, and analysed	8
		(b) Give reasons for non-participation at each stage	NA
		(c) Consider use of a flow diagram	NA

For peer review only - http://bmjopen.bmj.com/site/about/guidelines.xhtml

Descriptive data	14*	(a) Give characteristics of study participants (eg demographic, clinical, social) and information on exposures and potential confounders	8
		(b) Indicate number of participants with missing data for each variable of interest	8
Outcome data	15*	Report numbers of outcome events or summary measures	8-16
Main results	16	(*a*) Give unadjusted estimates and, if applicable, confounder-adjusted estimates and their precision (eg, 95% confidence interval). Make clear which confounders were adjusted for and why they were included	8-16; Supp 2
		(*b*) Report category boundaries when continuous variables were categorized	NA
		(*c*) If relevant, consider translating estimates of relative risk into absolute risk for a meaningful time period	NA
Other analyses	17	Report other analyses done—eg analyses of subgroups and interactions, and sensitivity analyses	NA
**Discussion**			
Key results	18	Summarise key results with reference to study objectives	17,18
Limitations	19	Discuss limitations of the study, taking into account sources of potential bias or imprecision. Discuss both direction and magnitude of any potential bias	18,19
Interpretation	20	Give a cautious overall interpretation of results considering objectives, limitations, multiplicity of analyses, results from similar studies, and other relevant evidence	17, 18, 20
Generalisability	21	Discuss the generalisability (external validity) of the study results	17,18
**Other information**			
Funding	22	Give the source of funding and the role of the funders for the present study and, if applicable, for the original study on which the present article is based	21

*Give information separately for exposed and unexposed groups.

Note: An Explanation and Elaboration article discusses each checklist item and gives methodological background and published examples of transparent reporting. The STROBE checklist is best used in conjunction with this article (freely available on the Web sites of PLoS Medicine at http://www.plosmedicine.org/, Annals of Internal Medicine at http://www.annals.org/, and Epidemiology at http://www.epidem.com/). Information on the STROBE Initiative is available at www.strobe-statement.org.

For peer review only

For peer review only - http://bmjopen.bmj.com/site/about/guidelines.xhtml

BMJ Open

Understanding the roles and work of paramedics in primary care: A national cross-sectional survey

Journal:	*BMJ Open*
Manuscript ID	bmjopen-2022-067476.R1
Article Type:	Original research
Date Submitted by the Author:	04-Nov-2022
Complete List of Authors:	Eaton, Georgette; Oxford University, Nuffield Department of Primary Care Health Sciences<br>Tierney, Stephanie; Oxford University, Nuffield Department of Primary Care Health Sciences<br>Wong, Geoff; Oxford University, Nuffield Department of Primary Care Health Sciences<br>Oke, Jason; Oxford University, Nuffiled Department of Primary Care Health Sciences<br>Williams, Veronika; Nipissing University, Faculty of Education and Professional Studies - School of Nursing<br>Mahtani, Kamal; Oxford University, Nuffield Department of Primary Health Care Sciences
**Primary Subject Heading**:	General practice / Family practice
Secondary Subject Heading:	Health policy
Keywords:	PRIMARY CARE, Organisation of health services < HEALTH SERVICES ADMINISTRATION & MANAGEMENT, Quality in health care < HEALTH SERVICES ADMINISTRATION & MANAGEMENT

SCHOLARONE™
Manuscripts

I, the Submitting Author has the right to grant and does grant on behalf of all authors of the Work (as defined in the below author licence), an exclusive licence and/or a non-exclusive licence for contributions from authors who are: i) UK Crown employees; ii) where BMJ has agreed a CC-BY licence shall apply, and/or iii) in accordance with the terms applicable for US Federal Government officers or employees acting as part of their official duties; on a worldwide, perpetual, irrevocable, royalty-free basis to BMJ Publishing Group Ltd ("BMJ") its licensees and where the relevant Journal is co-owned by BMJ to the co-owners of the Journal, to publish the Work in this journal and any other BMJ products and to exploit all rights, as set out in our licence.

The Submitting Author accepts and understands that any supply made under these terms is made by BMJ to the Submitting Author unless you are acting as an employee on behalf of your employer or a postgraduate student of an affiliated institution which is paying any applicable article publishing charge ("APC") for Open Access articles. Where the Submitting Author wishes to make the Work available on an Open Access basis (and intends to pay the relevant APC), the terms of reuse of such Open Access shall be governed by a Creative Commons licence – details of these licences and which Creative Commons licence will apply to this Work are set out in our licence referred to above.

Other than as permitted in any relevant BMJ Author's Self Archiving Policies, I confirm this Work has not been accepted for publication elsewhere, is not being considered for publication elsewhere and does not duplicate material already published. I confirm all authors consent to publication of this Work and authorise the granting of this licence.

For peer review only

TITLE PAGE

Title: Understanding the roles and work of paramedics in primary care: A national cross-sectional survey

Corresponding Author:

Georgette Eaton

Nuffield Department of Primary Care Health Sciences
University of Oxford
Radcliffe Primary Care Building
Radcliffe Observatory Quarter
Woodstock Road
Oxford
OX2 6GG
Georgette.eaton@phc.ox.ac.uk
Tel: +44 (0)1865 617816

Co-Authors:

Dr Stephanie Tierney
Nuffield Department of Primary Care Health Sciences
University of Oxford
Radcliffe Primary Care Building
Radcliffe Observatory Quarter
Woodstock Road
Oxford
OX2 6GG

Dr Geoff Wong
Nuffield Department of Primary Care Health Sciences
University of Oxford
Radcliffe Primary Care Building
Radcliffe Observatory Quarter
Woodstock Road
Oxford
OX2 6GG

Dr Jason Oke
Nuffield Department of Primary Care Health Sciences
University of Oxford
Radcliffe Primary Care Building
Radcliffe Observatory Quarter

For peer review only

Woodstock Road
Oxford
OX2 6GG

Dr Veronika Williams
100 College Drive
Box 5002
North Bay
ON
Canada
P1B 8L7
veronikw@nipissingu.ca
Tel: 705.474.3450

Dr Kamal R. Mahtani
Nuffield Department of Primary Care Health Sciences
University of Oxford
Radcliffe Primary Care Building
Radcliffe Observatory Quarter
Woodstock Road
Oxford
OX2 6GG

Keywords:

1. PRIMARY CARE
2. Organisation of health services
3. Quality in health care

Word Count: 4846

For peer review only

ABSTRACT

OBJECTIVES: This research aimed to fill a current knowledge gap, namely the current scope of clinical role of paramedics in primary care, in relation to specific constructs such a level of education and clinical experience.

SETTING The survey was distributed to paramedics in primary care across the UK through the College of Paramedics.

PARTICIPANTS A total of 341 surveys were returned (male=215). 90% of responses were from paramedics in England, 1.7% from paramedics in Northern Ireland, 4.6% from paramedics in Scotland, and 2.9% from paramedics in Wales. This represents approximately 33% of the primary care paramedic workforce in England and Wales. Estimates for percentages in Northern Ireland and Scotland are unavailable due to the lack of workforce datasets capturing paramedics in primary care.

RESULTS Considerable variation was found in job titles, level of education and provision of clinical supervision of paramedics in primary care. Differing levels of practice were noted, despite guidance documents that attempt to standardise the role. Statistical analysis of quantitative data highlighted that relationships exist between paramedic clinical exposure in primary care, level of education, and ability of independently prescribe medicines and the extent to which clinical presentations are seen and examinations performed. However, free text responses indicated that challenges in relation to access to further education and clinical supervision to support clinical development resulted in frustration for paramedics who work in this setting.

CONCLUSIONS As well as offering an insight into the demographics of the primary care paramedic work force, there is indication of the clinical scope of role undertaken in this setting. Based on our findings, we recommend changes to education and support, governance, and legislation to ensure paramedics employed in primary care can work to achieve the full extent of their professional capability.

STRENGTHS AND LIMITATIONS

- The survey was distributed by the UK College of Paramedics and on social media, and so may not capture the experiences of paramedics not members of their professional body or without profiles on social media.
- There was a low uptake in responses from paramedics working in Northern Ireland and Scotland.
- All (n=341) respondents answered all questions within the survey
- The sample size power calculation was met, assuring an adequate power to detect statistical significance in the results.
- The survey was undertaken during September-November 2021, during which time UK primary care was continuing to respond to the ongoing global pandemic of COVID-19

INTRODUCTION

Over the last decade, paramedics in the United Kingdom (UK) have increasingly taken up clinical employment away from ambulance services, with many moving into primary care settings (1). Reasons for this move are multifactorial and interwoven. Changes to healthcare access have created a sociocultural dependence on the ambulance service (2), resulting in an increase of urgent and primary care related dispositions attended by paramedics (3). To respond to the changing demands of 999 calls, paramedic pre-registration education prepares registrants to deliver holistic care across the lifespan (4), moving beyond protocolised training for the management of emergency presentations, for which paramedics have historically been most well-known. This broad undergraduate education defines the pluripotential nature of paramedics, enabling them to act as generalist clinicians in a range of clinical settings – and ultimately enables them to leave the ambulance service. This, coupled with poor managerial support and a lack of clinical progression within the ambulance service (5), is resulting in paramedics seeking opportunities to work in other clinical settings which offer further development and an improved work-life balance (6). Occurring at a time where the primary care workforce is understaffed due to a failure to recruit and retain General Practitioners (GPs) (7), paramedics actively seek opportunities to work in primary care, believing their capabilities fit well within this clinical setting (8). Indeed, recent NHS workforce policy welcomes this professional group into primary care (9–11), with associated funding through the Additional Roles Reimbursement Scheme (AARS) available for practices employing paramedics (amongst other health and social care staff) in England (12).

Our previous research has highlighted that for paramedics to work successfully as part of the primary care team, transition into this workforce needs to be supported through a combination of formal education, clinical supervision, and socialisation (8). Desired outcomes, such as increasing clinical capacity within the primary care team (and filling workforce gaps created by the shortage of GPs), may then transpire. Health Education England (HEE) has made steps to provide a framework that addresses these concepts. HEE is a non-departmental public body, which provides coordination and support for the training and education within England's healthcare workforce. In 2019, HEE set out a 'roadmap' for paramedics to follow as they transition into primary care

For peer review only

roles (13). This document outlines the qualifications, capabilities, clinical skills, and case presentations paramedics are expected to encounter whilst working in primary care. This is the only published document across the devolved nations that sets any scope of role for paramedics working in this clinical setting.

Paramedics are being actively recruited into the primary care workforce, but knowledge gaps remain. This research specifically aims to fill a current knowledge gap, namely the paramedics' scope of role in primary care and their perception of their roles. This research records the current scope of clinical role of paramedics in primary care, in relation to specific objectives:

- To better understand the patterns of education level, experience, salary, prescribing status, and clinical supervision for paramedics in primary care.
- To investigate the scope of role undertaken by paramedics in NHS primary care.
- To explore the perceptions paramedics in primary care have on their contribution to primary care teams.

For peer revi

METHODS

Prior to commencing this survey, a study protocol was developed and registered with OSF Registries (10.17605/OSF.IO/YKDA7). We report our findings according to the Strengthening the Reporting of Observational Studies in Epidemiology (STROBE) Statement (14).

Study design

An online survey was distributed via the College of Paramedics to paramedics in primary care in England, Northern Ireland, Scotland, and Wales. The survey utilised both qualitative and quantitative items in recognition of the polygonal objectives of the study (15). Details regarding workforce data for paramedics in primary care are reported for England (16) and Wales (17), but not yet for Northern Ireland or Scotland. Based on the available data in England and Wales, we estimated that approximately 1,500 paramedics were working in primary care roles across the United Kingdom in August 2021, and therefore considered a sample size of 306 would be needed for a confidence interval of 95% and margin of error of 5% for statistical analysis.

Materials and procedure

The survey was developed by authors in consultation with members of a patient and public involvement group, and stakeholders from the College of Paramedics, HEE, and the Royal College of General Practitioners. The survey was first piloted with paramedics within this stakeholder group and refined by the authorship team. This pilot assisted in the flow of questions, and the format of question presentation. The final survey was presented using Jisc online surveys (https://www.onlinesurveys.ac.uk/) (online supplemental file 1). Data collection took place between 1st September and 30th November 2021.

Distribution of the survey was initially via internal communications within the College of Paramedics, as well as across their social media platforms. This survey was shared further on social media to reach paramedics working in primary care who may not be members of the College of Paramedics.

Respondents were offered a £10 Amazon e-voucher in compensation for their time to complete the survey.

Data analysis

Free-text responses were analysed using semantic level, inductive thematic analysis in NVivo v.12 (18), undertaken by GE. Ten per cent of these codes were reviewed by ST. Quantitative data were analysed using descriptive statistics (mean, SD, and frequencies) and appropriate non-parametric tests (Chi-Square Test of Independence, Kruskal-Wallis Test, Mann-Whitney test, Spearman's rho correlation) in IBM SPSS Statistics, Version 28 (IBM Corp., Armonk, N.Y., USA). Bonferroni correction was used to counteract problems where multiple comparisons occurred, and this adjustment is included in the results reported in this paper. Statistical analysis was undertaken by GE and reviewed independently by JO.

Quantitative and qualitative data were initially analysed separately, then merged and interpreted (19). During interpretation, data was considered in relation to existing conceptual frameworks (8) to enrich findings and synthesise complementary results.

Patient Involvement

The patient and public group associated with this research were involved during the design of the survey instrument and the analysis of the qualitative results.

RESULTS

A total of 341 responses were returned, of which 90.6% were from paramedics working in primary care in England (n=309). Based on workforce data published for the period within which the survey was undertaken (September-November 2021) (16,17), our respondents represent 33% of the population for England and Wales separately. Table 1 outlines descriptive characteristics of survey respondents according to age range, gender, and country of work. All respondents answered each question in the survey.

Table 1. Description of survey respondents

Age Range	n	Gender	n	Country of work	n
18-24 years old	4	Female	126	England	309
25-34 years old	98	Male	215	Northern Ireland	6
35-44 years old	113			Scotland	16
45-54 years old	96			Wales	10
55-65 years old	30				

Experience as a paramedic prior to working in primary care

Prior to working in primary care, most respondents had 3-5 years (n=93) or 6-10 years (n=93) experience of working as a paramedic. When asked why they chose to work in primary care, a majority cited an improved work/life balance as the single biggest contributing factor. This was often referred to in relation to a move away from the unsustainable workload in the ambulance service, especially working hours that were considered family friendly, with no night or weekend shifts. Other factors prompting paramedics to take up employment in primary care included the opportunity for clinical development; increased job satisfaction associated with the increased autonomy and enhanced clinical skill set required to work in primary care; and a clinical interest in holistic and preventative medicine associated with general practice.

For peer review only

Level of education

The majority (52%) of respondents were educated to at least one module at FHEQ Level 7/SCQF Level 11, with more than half of these educated to postgraduate certificate (n=90). Figure 1 outlines the level of education for respondents. The need for additional knowledge to work in primary care was articulated. Respondents often reported a "*constant pressure to study*" (RID 118; Emergency Care Practitioner), and a sense of overwhelm regarding the volume of academic work alongside their clinical workload. Both a lack of protected time to study, as well as absence of funding from primary care employers for paramedics to undertake education qualifications, were highlighted as a hinderance to development in primary care.

Effect of HEE Roadmap

For respondents in England, the 'roadmap' published by HEE (13) was viewed to assist in the standardisation and supervision of the role (n=42), but was often poorly executed at an employment level, due to lack of clarity in the roadmap's requirements (n=21), ease of fulfilment (n=25), or uptake by employer (n=21). 57 respondents reported the roadmap had no influence on their employment or practice in primary care:

"I'm not ARRS funded so doesn't impact me. I'm aware of the roadmap but my surgery isn't pressuring me to complete it. They know my skill set." (RID 327; Paramedic Practitioner).

Clinical Supervision

85.6% (n=292) of respondents received clinical supervision within their role. When asked to what extent the clinical supervision meets their needs, 25 respondents reported a clinical supervision model that was regular and structured. Other experiences of supervision were "v*ery poor due to limited time with supervisor*" (RID 51; Paramedic Practitioner).

"I don't feel the GPs really understand the role yet or have the time to support it in a way that would meet the expectations set out by the… roadmap." (RID 199; Paramedic Practitioner).

For peer review only - http://bmjopen.bmj.com/site/about/guidelines.xhtml

Whilst a trend was seen concerning the provision of clinical supervision and the extent to which clinical presentations were seen and clinical examinations undertaken, this failed to reach statistical significance (Tables A and B, online supplemental file 2).

Job title

Job titles for paramedics working in primary care were varied and inconsistent (Figure 2). Inconsistency in job titles was suggested to contribute to discrepancies in scope of practice and understanding of the role from other health care professionals in primary care:

"I still feel that there is a lack of understanding, or clear delineation between roles… I am unsure if those at my surgery are aware of the difference between a Paramedic Practitioner and Advanced Clinical Practitioner…" (RID 305; Advanced Clinical Practitioner).

As well as patients:

"I don't think [my job title] *reflects my role and is unclear to patients."* (RID 150; Emergency Care Practitioner).

It appears that job title (and thus seniority of paramedic) made a difference to the extent to which a diagnosis is made during a consultation (H(3)=15.73, p=<.001), the management of medical and clinical complexity (H(3)=15.73, p=<.001), and leadership and management (H(3)=10.507 p=.015) undertaken by paramedics.

Salary

The most common salary bracket was between £33,222-£43,041 (Figure 3). Significant associations were found between salary and job title (X^2(15)> = 51.137, p= <.001), prescribing status (X^2(5)> = 118.190, p=<.001), highest qualification (X^2(30)> = 72.589, p=<.001), length of time as a paramedic (X^2(40)> = 101.212, p=<.001), and length of time in primary care (X^2(25)> = 112.780, p=<.001).

There was no significant association between salary and gender (X^2(5) = 11.936, p=.036), or salary and region of work within the UK (X^2(15) = 28.589, p=.018).

For peer review only

Length of time working in primary care

Length of time in primary care differed with job title (H(3)=14.145, p=003), where seniority of job title was associated with increased time in primary care. Elements of the clinical work associated with length of time in primary care are reported in Tables A and B within online supplemental file 2.

Hours worked in primary care

Significant associations were found between hours worked and employment type (X^2(15)>=129.872, p=<.001), where there was an association found between respondents employed directly with the practice and working full time hours (n=126), and those working in rotational roles between ambulance services and primary care providers working one day a week (n=22).

Core capabilities of paramedics working in primary care

We assessed the relationships between the extent to which the core capabilities of primary care set out within HEE's Roadmap (13) formed part of the respondent's role, against employment type, length of time registered as a paramedic, length of time in primary care, level of education, hours worked, job title, independent prescribing status, and salary. Positive relationships exist between these capabilities and increase in each of these factors. The strength of these correlations are outlined in Table C of online supplemental file 2.

However, some respondents experienced frustration regarding their progression within these core capabilities:

"Keen to undertake a leadership role but don't see this being an option. Very much bums on seats." (RID 92; Advanced Clinical Practitioner).

Clinical work undertaken

To understand the breadth and frequency of clinical work undertaken, respondents were asked the extent to which they saw the range of clinical presentations (Figure 4) and the extent to which they undertook clinical examinations (Figure 5) outlined in HEE's Roadmap (13).

For peer review only

Relationships between clinical presentations existed between length of time as a paramedic, length of time in primary care, hours worked in primary care, highest qualification, job title, and prescribing status. Relationships between clinical examinations undertaken also existed between length of time in primary care, hours worked in primary care, highest qualification, job title, and prescribing status. These results are outlined in Tables A and B in online supplemental file 2.

Blood Tests

The request and interpretation of blood tests by paramedics in primary care was positively influenced by prescribing status, the receipt of clinical supervision, increased salary, hours worked (full time), and higher levels of education – however these results largely failed to meet statistical significance. There was no correlation between the request and interpretation of blood tests and length of time as a paramedic, nor length of time in primary care. These results are outlined in Table D online supplemental file 2.

Patient Groups not seen

When asked about patient groups not seen, many respondents (n=189) would not see presentations relating to women’s health (including intimate examination, pregnant patients with directly related pregnancy issues, sexual health, or menopause). Other common patient groups not seen by paramedics included children, particularly under the age of two (n=114), and presentations relating to mental health (n=69). In addition, respondents saw clinically complex patients, palliative care, and chronic conditions less than other patient groups.

Emergency Skills

Significant correlations were found between level of education and the undertaking of emergency procedures (r_s=--.156, p=<.004), where respondents with lower qualifications undertook emergency procedures to a greater extent. A similar correlation was found between the level of education and attending presentations such as cardiac arrest (r_s=-.106, p=.049), catastrophic haemorrhage (r_s=-.119, p=.028), shock (r_s=-.173, p=.001), cardiovascular adverse signs (r_s=-.120, p=<.027), and limp children (r_s=.125, p=.021), again where respondents with lower qualifications attended these presentations to a greater extent. This was also reflected in the free-text

comments, where some paramedics *"…do not feel challenged by only dealing with same day emergency consultations."* (RID 14; Paramedic).

There was also a correlation between hours worked and attending presentations such as catastrophic haemorrhage (r_s=.109, p=.044), anaphylaxis (r_s=.127, p=.019), angioedema (r_s =.140, p=.009), seizures (r_s =.147, p=.007), and overdose/poisoning (r_s =.200, p=<.001), where respondents who worked less hours in primary care (such as one day a week or 10-20 hours per week) attended these presentations to a greater extent. It was also noted that these respondents were employed primarily by ambulance services, and so likely to be working in rotational roles.

Free-text comments outlined that respondents who had been employed for a longer time in primary care missed the opportunity to attend higher acuity patients, and also reported a loss of paramedic identity without the opportunity to practice their emergency skills.

Prescribing status

125 respondents (36.7%) were independent prescribers, with a further 57 (16.7%) undertaking the course at the time of the survey, and a further 137 (38.7%) wishing to undertake the course in the future. Inability to prescribe frustrated 20 respondents when asked about the frustrations in their role.

Significant associations were found between prescribing status and clinical assessment skills outlined in Table E online supplemental file 2, where independent prescribers were more likely to undertake these examinations when compared to paramedics who could not prescribe.

Role frustrations

Short appointment times and volume of patients within the day contributed to high workload and feelings of frustrations about their role in primary care. General system frustrations were also reported regarding the structure, organisation, and funding of primary care. In addition, the impact of the ongoing global pandemic of coronavirus disease (COVID-19) on the working practices of respondents caused frustration, with experiences of an increase in telephone consultation and aggression from patients.

For peer review only

Respondents reported frustration when faced with restrictions in practice, commonly due to an inability to prescribe Schedule 2 controlled medicines, or to provide patients with a Statement of Fitness for Work. Other restrictions in practice included an ability to fit contraceptive devices, as well as certain procedural skills such as undertaking intimate examination.

Another frustration reported was a lack of understanding of the capabilities of the paramedics in primary care, from both colleagues and patients:

"Sometimes there are major misconceptions about what paramedics can and can't do. Most management have no idea about the role of a paramedic." (RID 326; First Contact Practitioner Paramedic).

"I am well qualified for my role, I am experienced. But I work alongside doctors who do not understand my capabilities." (RID 15; Advanced Clinical Practitioner, Paramedic).

Role gratification

When asked about the difference respondents felt they made in the role, this was considered between the difference they felt they made in the primary care workforce, the difference to patients, and the difference their role had on the paramedic profession overall.

Primary care

Just under half of respondents felt their role in primary care increased workforce capacity (n=161, 47%). This was in the context of freeing up time "*for GPs to manage complex conditions*" (RID 9; Advanced Paramedic Practitioner); facilitating multi-disciplinary team working, and being able to capitalise on their unique paramedic skills:

"*Our clinical knowledge with ability to make autonomous safe decisions with the ability to recognise big sick / little sick quickly means that we are a strong spoke in the wheel of general practice."* (RID 303; Specialist Paramedic).

For peer review only

Patients' satisfaction

Respondents measured the satisfaction of patients who they had seen in their role by virtue of positive feedback received, and patients who actively sought consultations with them again in the future, or did not reattend for the same presenting complaint. This patient satisfaction was attributed to the comprehensive consultations undertaken by paramedics, which they reported as benefitting from increased time slots, and the discussion of medical conditions in a simple language patients could understand. Indeed, paramedics viewed their interpersonal skills as one of their greatest strengths regarding patient satisfaction:

"For the most part people are happy to see a professional that listens to them, treats them with respect and dignity and is competent. I think a lot of paramedics offer this in primary care." (RID 135; Advanced Clinical Practitioner).

Experiences of patient dissatisfaction following consultation by a paramedic in primary care were also reported by respondents. These occurred when patients were disappointed that the consultation was not with a GP, and where there was a lack of understanding of the paramedic role, leading them to question the clinical expertise paramedics could offer:

"Some patients do not understand my role/skill set and just want a GP." (RID 143; Advanced Paramedic).

Profession

Respondents considered their role in primary care in relation to promotion and understanding of the profession, which "*shows that there is so much more than being a paramedic on the back of an ambulance*" (RID 144; Paramedic Practitioner). Other respondents considered this in relation to the development of the profession as a whole:

"I feel as though the level of autonomous clinical decision making, management of complex cases, referrals and medicine management we are practising is at a much higher level than a large amount of the profession. I think it sets a good example to

For peer review only - http://bmjopen.bmj.com/site/about/guidelines.xhtml

other paramedics and other medical colleagues alike that paramedics can be pushed beyond traditional roles and expectations and can prove to be valuable part of clinical team." (RID 202; Advanced Paramedic).

The impact of the move to work in primary care was also acknowledged in context of the wider healthcare workforce. Respondents outlined this both in terms of leaving the ambulance service to undertake roles in primary care, as well as rotational working between ambulance services and primary care settings. This was considered to have benefit regarding the transferability of clinical knowledge and skills from one clinical setting to the other:

"My rotational role increases understanding between ambulance & primary care, increases my knowledge & understanding which I can pass on to colleagues and increases visibility, trust and understanding of the profession throughout primary care and the lay community." (RID 36; Specialist Paramedic).

DISCUSSION

Main findings of this study

This research confirms previous publications which noted variance in a) job title reported by paramedics working in primary care; b) the clinical work and examinations undertaken by paramedics in this setting; c) entry requirements in terms of clinical experience and education to work in primary care (1,8). This level of variation subsequently leads to confusion around the scope and expectations for the role and contributes to a lack of recognition of paramedics within primary care teams. Whilst attempts have been made through HEE's Roadmap (13) to outline a framework to address this, this is applicable only in England, and such a framework has no influence for paramedics across the devolved nations. The main barriers to engagement with this Roadmap were competing workload pressures affecting the delivery of clinical supervision and uptake of this framework by primary care employers. Such inconsistency contributed to frustration and demotivation among respondents, who were concerned regarding their clinical development in this setting.

For peer review only

Clinical examinations and procedural skills

Our analysis indicates that length of time in primary care, higher levels of education, and status as an independent prescriber all contribute to an increase in the scope of role for paramedics in primary care. Indeed, an inverse association was also observed, where paramedics with lower educational qualifications attended emergency presentations within primary care to a greater extent than those who had undertaken higher education. This outlines that, whilst the paramedic may transition into primary care due to the virtue of their generalist background, their productivity in primary care may be influenced by further education and feedback regarding their clinical experience in this setting. Despite this generalist background, this survey also outlines there are patient groups commonly not seen by paramedics. This could be due to a creep into the paramedic role of nursing policy which emphasise that nurses should refer women who are pregnant to midwifery or physician care if they are not dual registered in this area (20); and legacy of instructions for paramedics in ambulance services to convey all children under the age of two to emergency departments, and children under the age of five must be seen by a physician if non-conveyed (21).

The survey also highlighted that paramedics working less hours in primary care (such as one day a week or 10-20 hours per week) attended emergency presentations in their primary care role to a greater extent when compared to their full-time counterparts. Such hours are common in rotational models, where paramedics, specialist paramedics or advanced paramedics work in a split clinical role between ambulance services and primary care settings in an attempt to increase workforce capacity in primary care and reduce attrition from the ambulance service (22). Whilst the ability for paramedics to attend emergency presentations in primary care may be a benefit for primary care providers, this does little to develop their primary care clinical acumen.

Paramedic taxonomy

Some job titles reported by respondents match those endorsed by the College of Paramedics (23) (such as ‘Paramedic’, ‘Specialist Paramedic’, ‘Advanced Paramedic’, or ‘Consultant Paramedic’) or those outlined by HEE (13) (such as ‘First Contact Practitioner’ and ‘Advanced Practitioner’), yet there remains a variety of job titles that do not correlate to these archetypes.

For peer review only - http://bmjopen.bmj.com/site/about/guidelines.xhtml

Our analysis indicates that, as paramedics take up more senior roles in primary care (such as ‘advanced paramedic’), their scope of role increases in relation to clinical examinations performed, and the clinical presentations they attend. Such an increase in scope could be due to their ability to independently prescribe and undertaking postgraduate study. Independent prescribing is typically undertaken by ‘advanced paramedics’ who have completed (or working towards completion of) a Master’s degree (24). We noted that these paramedics are more likely to make a diagnosis during the consultation and manage medical and clinical complexity. This is in contrast to ‘paramedics’ or ‘first contact practitioners’, who may have a similar scope of clinical examination, but a reduced scope in relation to managing clinical complexity and making a diagnosis. This supports previous findings where such paramedics are employed in an ‘eye and ears’ approach only (8).

There was a strong correlation with advanced and consultant level roles and undertaking activities related to leadership and management in primary care. This suggests that paramedics may move into leadership roles within primary care that have traditionally been filled by GPs. However, there was no correlation regarding undertaking research activities and job title. This indicates that research activities are less accessible to paramedics in primary care, despite being a pillar of advanced practice, matching previous research findings (25).

Strengths and weaknesses of the study

This is the first national survey of the paramedic role in primary care within the UK. It has international relevance for primary care workforce transformation in countries where paramedics operate in a similar way to in the UK, such as in Australasia and Canada (26). Whilst the survey was distributed across each UK nation, this was either through the College of Paramedics, or on social media – and thus paramedics not registered with the professional body, or not on social media, may not have had access. At best, the surveyed respondents constitute one-third of the paramedic workforce in primary are, and therefore results should not be generalised to the entire population of paramedics working in the primary care.

For peer review only

It is noted that the uptake of the survey in Northern Ireland and Scotland was low. Numbers of paramedics working in primary care roles is likely to be fewer than in England, however the paramedic role is currently not captured in workforce data for these countries (27,28), so the actual number of paramedics in primary care in these countries is unknown. However, there was no variation by region reported in the clinical work undertaken, outlining that paramedics are working in a similar way across the UK. Nevertheless, we recognise that paramedics from Northern Ireland and Scotland may be underrepresented in this survey.

The use of data triangulation within the cross-sectional survey allowed for the exploration of both the distribution and variety of roles paramedics undertake in primary care. Additional free-text responses also enabled further understanding of the support and frustrations paramedics experience when working in primary care. However, in the interpretation of the quantitative data we undertook multiple statistical tests. Whilst adjustments were made to control family-wise error rate, it is possible that some of the marginal results produced are not robust. Further research, using different variable types, could build on these findings to determine their strength.

It is appreciated that data collection took part during the ongoing global pandemic of COVID-19. As this is the first national survey of the paramedic role in primary care, it is unclear what impact this ongoing pandemic has on these results. Future surveys may be able to determine this.

RECOMMENDATIONS

Based on our findings above we make a number of recommendations:

Education and support

- There is a need to further standardise required education and training for paramedics working in primary care.
- There should be systems in place within primary care settings to ensure paramedics have access to structured clinical supervision.
- Support should be given to employers to enable them to effectively support the paramedic in primary care and utilise this healthcare professional to the greatest benefit.

For peer review only

Governance

• There is a need to standardise job titles, salaries, and descriptions for paramedics working in primary care to ensure role recognition by both patients and the wider primary care team.

• A standard scope of role for paramedics working in primary care that is applicable to all nations is needed.

• Clear career pathways for paramedics working in primary care should be established, to maximise retention and job satisfaction in this clinical setting.

Changes to legislation

• Changes to legislation to support independent prescribing of controlled medications.

• Ability for paramedics to provide a Statement of Fitness for Work.

CONCLUSION

As the first survey of the paramedic role in primary care across the UK, this study indicates the demographic range of paramedics working in primary care, and the common clinical presentations and examinations undertaken by this workforce. As well as offering an insight into the clinical scope of role undertaken in this setting, we have highlighted that relationships exist between paramedic clinical exposure in primary care, level of education, and ability of independently prescribe medicines and the extent to which clinical presentations are seen and examinations performed. As well as policymakers, this is important information for primary care employers seeking to employ, or develop, paramedics in this practice setting.

For peer review only

FIGURE LEGEND

Figure 1. Level of education of respondents

Figure 2. Job title of respondents.

Figure 3. Salary of respondents

Figure 4. A treemap of clinical presentations attended by respondents as a paramedic in primary care

Figure 5. A treemap of clinical examination undertaken by respondents as a paramedic in primary care

For peer review only

ACKNOWLEDGEMENTS

We would like to thank the patient and public group involved with this research for their time and comments during the design of the survey and the analysis of the results. We would also like to thank our stakeholders from the College of Paramedics, Health Education England, the Nuffield Trust, and the Royal College of General Practitioners with whom we discussed the development of our survey, and with whom we piloted the survey. Lastly, thank you to the paramedics across the UK who completed this survey, and the peer reviewers of this paper prior to publication.

Contributors

GE, ST, GW, KM, VW designed the survey. Data analysis was carried out by GE, JO and ST. The manuscript was drafted by GE, ST, GW, JO, VW and KM. All authors accept full responsibility for the work and/or the conduct of the study, had access to the data, and controlled the decision to publish. All authors read and approved the final manuscript

Funding

GE is supported by a National Institute for Health Research (NIHR) Doctoral Research Fellowship [NIHR300681]. This work is also supported by NHS Health Education England [ref: 190121].

Disclaimer

The views expressed are those of the authors and not necessarily those of the NHS, the NIHR, NHS Health Education England or the host institution. The funders of this research remained independent of the project design, data collection and data analysis.

Competing Interests

GE is a Trustee in the College of Paramedics. The recruitment of paramedics to the survey was made by application to the College of Paramedics Research Design Advisory Committee and was not associated with GE's trusteeship.

The remaining authors declare no competing interests.

For peer review only

Ethics approval

This study was approved by the Central University Research Ethics Committee (MS IDREC Ref: R64129/RE001. Respondents were free to decide whether to participate and informed consent was obtained at the start of the survey. Compensation was offered at completion of the survey, in the form of a £10 Amazon e-voucher. All data processing and storage comply with the General Data Protection Regulation and UK Data Protection Act 2018.

Data availability statement

Datasets are available upon reasonable request.

For peer review only

REFERENCES

1. Eaton G, Wong G, Williams V, Roberts N, Mahtani KR. Contribution of paramedics in primary and urgent care: A systematic review. *Br J Gen Pract* 2020; 70 (695): e421-e426
2. Wankhade P. Cultural characteristics in the ambulance service and its relationship with organisational performance: evidence from the UK. In: Does Culture matter Track. Nottingham: PAC Conference: 6-8 September; 2010.
3. Eaton G, Mahtani K, Catterall M. The evolving role of paramedics – a NICE problem to have? *J Health Serv Res Policy* 2018 Jul;23(3):193-195.
4. College of Paramedics. Paramedic Curriculum Guidance. 5th ed. Bridgwater: College of Paramedics; 2019. https://collegeofparamedics.co.uk/COP/ProfessionalDevelopment/Paramedic_Curriculum_Guidance.aspx (accessed 20 July 2022)
5. Beldon R, Garside J. Burnout in frontline ambulance staff. *JPP* 2021 Dec 30;14(1):6–14.
6. Mahtani KR, Eaton G, Catterall M, Ridley A. Setting the scene for paramedics in general practice: what can we expect? *J R Soc Med* 2018;111(6).
7. Marchand C, Peckham S. Addressing the crisis of GP recruitment and retention: a systematic review. *Br J Gen Pract* 2017];67(657):e227–37.
8. Eaton G, Wong G, Tierney S, Roberts N, Williams V, Mahtani KR. Understanding the role of the paramedic in primary care: a realist review. *BMC Med* 2021;19(1)
9. NHS England. The NHS Long Term Plan. London, NHS England 2019
10. NHS England. General Practice Forward View. London, NHS England 2016.
11. NHS England. Supporting General Practice in 2021/22. London, NHS England 2021.
12. NHS Confederation. Supporting population health management: workforce Recruiting paramedic practitioners through the Additional Roles Reimbursement Scheme (ARRS). 2021 https://www.nhsconfed.org/-/media/Confederation/Files/Publications/Documents/Paramedic-practitioners-ARRS.pdf (accessed 20 July 2022)
13. Health Education England. First Contact Practitioners and Advanced Practitioners in Primary Care: (Musculoskeletal) A Roadmap to Practice. London, Health Education England 2021.

For peer review only

14. Von Elm, E., Altman, D. G., Egger, M., Pocock, S. J., Gøtzsche, P. C., Vandenbroucke, J. P., & STROBE Initiative. The Strengthening the Reporting of Observational Studies in Epidemiology (STROBE) statement: guidelines for reporting observational studies. *Lancet* 2007 370(9596), 1453–1457. https://doi.org/10.1016/S0140-6736(07)61602-X
15. Czaja R, Blair J, Blair E. Designing surveys: A guide to decisions and procedures. London, SAGE Publications 2014.
16. NHS Digital. Headcount and Full-Time Equivalent (FTE) Time Series. General Practice Workforce: 2022 https://app.powerbi.com/view?r=eyJrIjoiYTM4ZTA3NGItMTM2Mi00NzAwLWEyY2QtNDgyZDkxOTk3MmFlIiwidCI6IjUwZjYwNzFmLWJiZmUtNDAxYS04ODAzLTY3Mzc0OGU2MjllMiIsImMiOjh9 (accessed 20 July 2022)
17. Welsh Government. Number of wider practice staff employed in general practices, by staff category and local health board. 2022 https://statswales.gov.wales/Catalogue/Health-and-Social-Care/General-Medical-Services/number-of-wider-practice-staff-employed-in-general-practices (accessed 20 July 2022)
18. Braun V, Clarke V. Thematic Analysis A Practical Guide. London: SAGE Publications 2022.
19. Zheng M. Conceptualization of Cross-Sectional Mixed Methods Studies in Health Science: A Methodological Review. *Int j quant qual res* 2015; 3(2):66–87.
20. Royal College of Nursing. Advanced Level Nursing Practice and Care of Pregnant and Postnatal Women. London; 2021.
21. National Ambulance Service Medical Directors. NASMeD Best Practice guidance: Conveyance of children by operational ambulance clinicians in face-to-face settings. London; 2021
22. Welsh Ambulance Services NHS Trust. Advanced Paramedic Practitioner Pilot-BCU Health Board Final Report. 2020 https://aace.org.uk/wp-content/uploads/2020/08/APP-Rotational-Pilot-Report.pdf (accessed 20 July 2022)
23. College of Paramedics. Post-Registration Career Framework. 4th ed. 2018 https://collegeofparamedics.co.uk/COP/ProfessionalDevelopment/post_reg_career_framework.aspx (accessed 20 July 2022)

For peer review only

24. College of Paramedics. Practice Guidance for Paramedics Independent & Supplementary Prescribers 2021 https://collegeofparamedics.co.uk/COP/Professional_development/Medicines_and_Independent_Prescribing/COP/ProfessionalDevelopment/Medicines_and_Independent_Prescribing.aspx?hkey=04486919-f7b8-47bd-8d84-47bfc11d821a (accessed 20 July 2022)

25. Fothergill J, Al-Oraibi A, Houdmont J, Conway J, Evans C, Timmons S, et al. Nationwide evaluation of the advanced clinical practitioner role in England: a cross-sectional survey. *BMJ Open* 2022; 12:55475 http://dx.doi.org/10.1136/bmjopen-2021-055475 (accessed 20 July 2022)

26. O'Meara P. Community paramedics: a scoping review of their emergence and potential impact. *International Paramedic Practice* 2014;4(1):5–12

27. Northern Ireland Statistics and Research Agency. General Medical Services for Northern Ireland, Annual Statistics 2019/20. Belfast; 2020 https://www.statisticsauthority.gov.uk/osr/code-of-practice/ (accessed 20 July 2022)

28. Public Health Scotland. General Practice Workforce Survey 2021 https://publichealthscotland.scot/media/9866/2021-10-26-gpworkforcesurvey2021-report.pdf (accessed 20 July 2022)

For peer review only

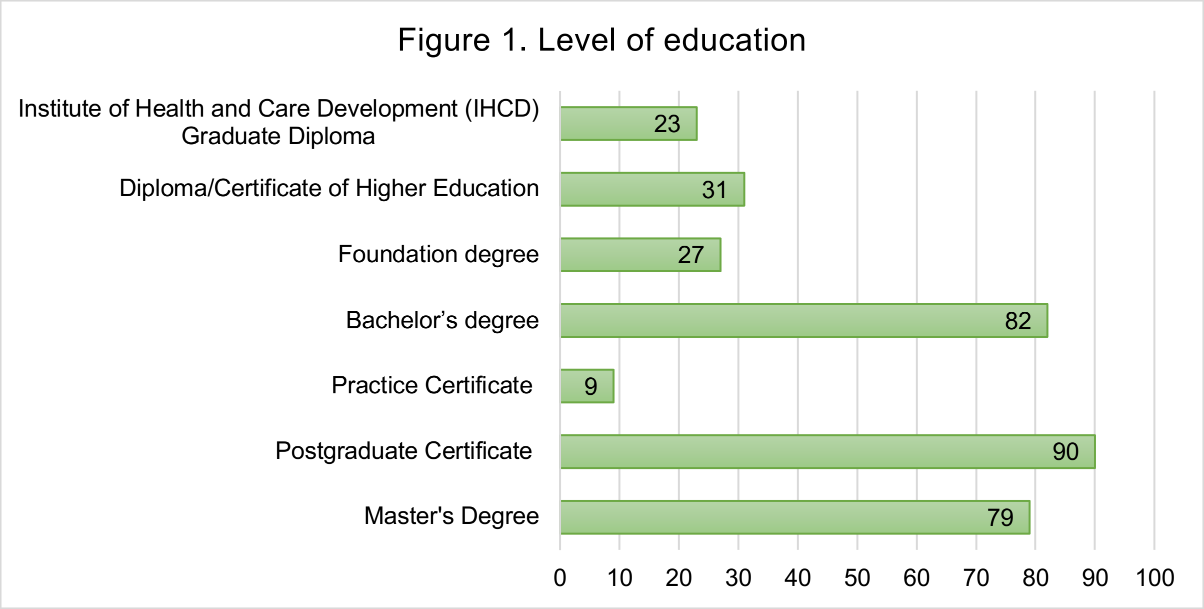

peer review only

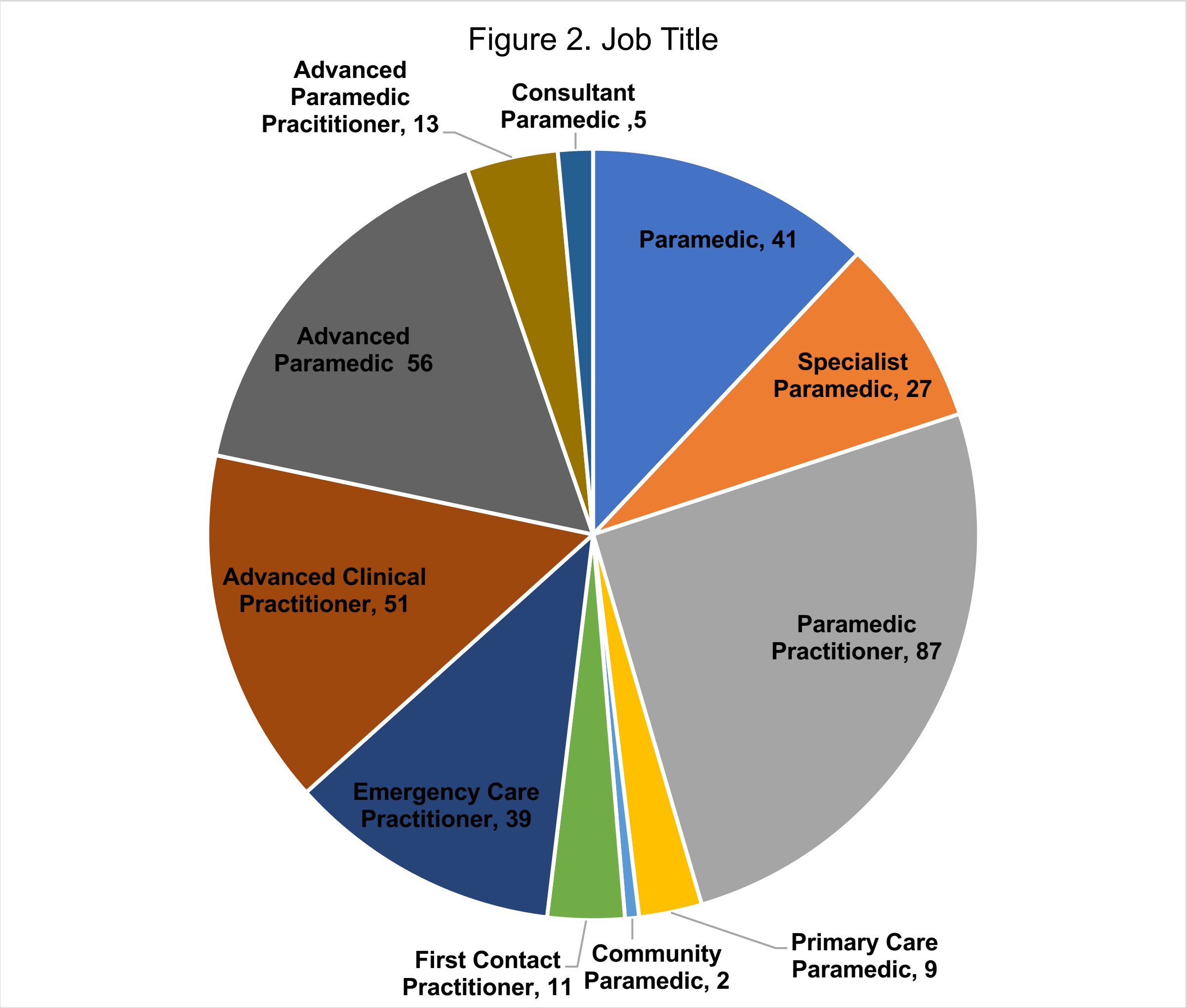
Figure 2. Job Title
Advanced Paramedic Pracititioner, 13
Consultant Paramedic ,5
Paramedic, 41
Specialist Paramedic, 27
Paramedic Practitioner, 87
Primary Care Paramedic, 9
Community Paramedic, 2
First Contact Practitioner, 11
Emergency Care Practitioner, 39
Advanced Clinical Practitioner, 51
Advanced Paramedic 56

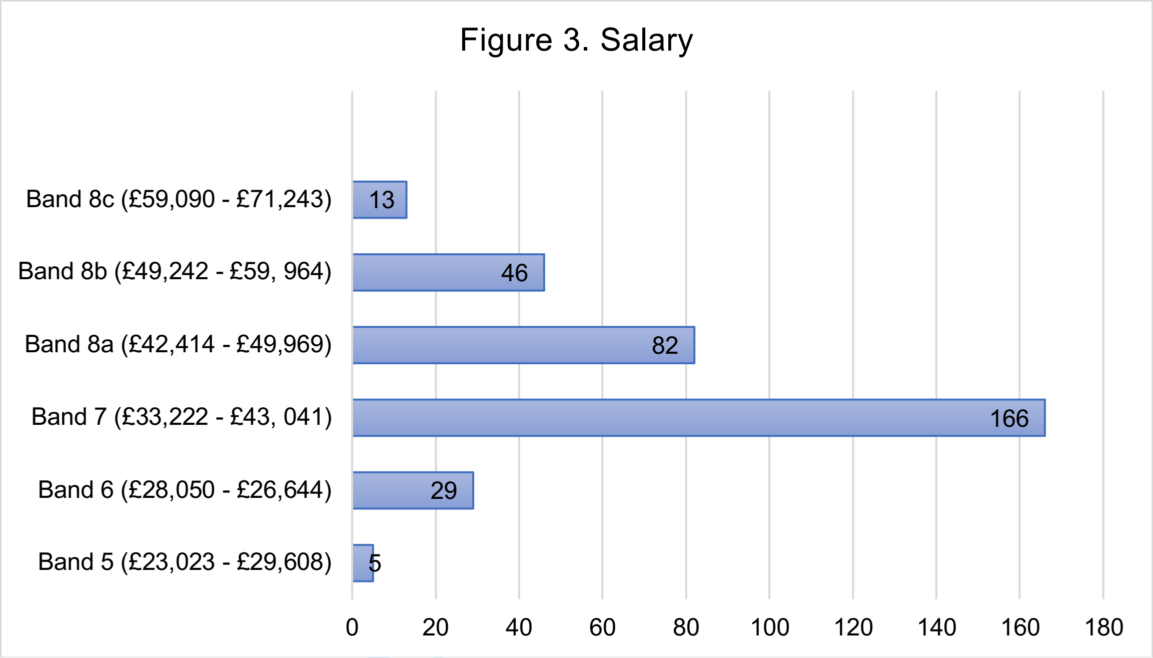

For peer review only

Figure 4. Treemap of Clinical Presentations attended by Paramedics working in Primary Care

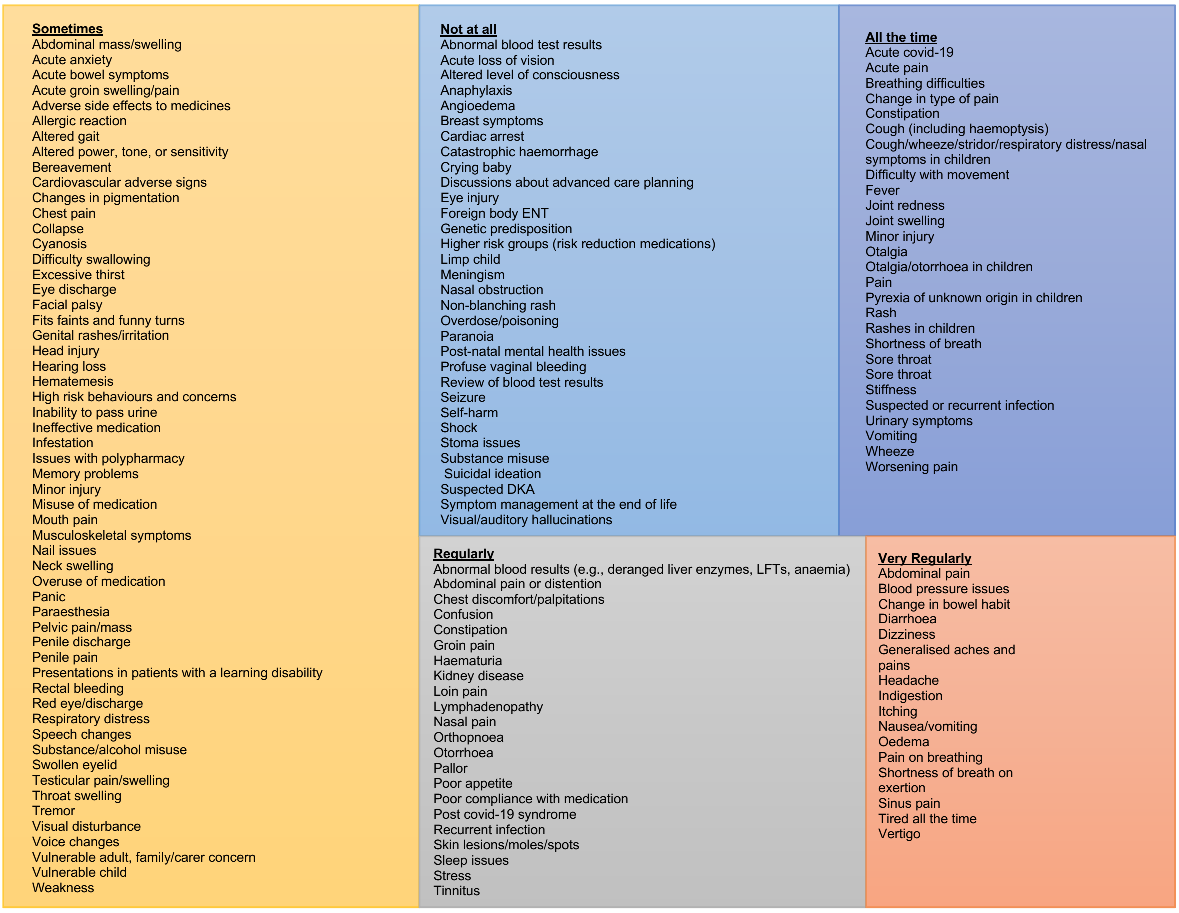

Figure 5. Treemap of Clinical Examinations undertaken by Paramedics working in Primary Care

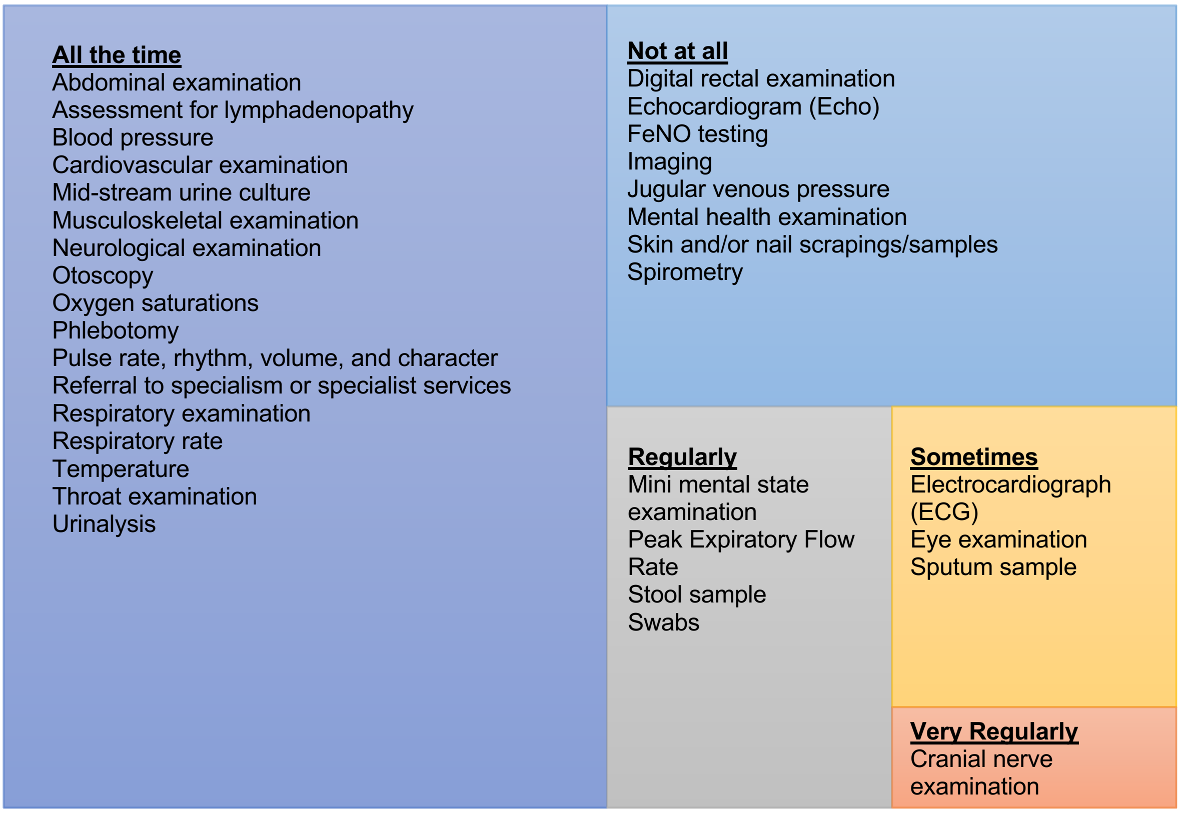

For peer review only - http://bmjopen.bmj.com/site/about/guidelines.xhtml

SURVEY QUESTIONNAIRE

Understanding the roles and work of paramedics in primary care: A national cross-sectional survey

What is this project about?

Thank you for your interest in participating in this questionnaire. This questionnaire is part of a study which aims improve understanding of the ways in which paramedics are working within the primary care workforce in the United Kingdom. Whilst paramedics are currently working in primary care roles, evidence must be generated to show how and why these changes would work, for whom, in what context and to what extent. That's why we're keen to understand from your perspective, as a paramedic working in primary care, the impact you feel you have and your perspectives of your role. We hope that the results of our research will influence future policy and professional change, as well as support paramedics working in primary care in the future.

You have been invited to participate as you are a paramedic working in primary care within the UK. Please read through this information before agreeing to participate (if you wish to) by ticking the 'yes' box below.

You may ask any questions before deciding to take part by contacting the researcher (details below).

The Principal Researcher is Georgette Eaton, who is attached to the Nuffield Department of Primary Care Health Services at the University of Oxford. This project is being completed under the supervision of Dr Kamal R. Mahtani, Dr Geoff Wong, Dr Stephanie Tierney and Dr Veronika Williams from the University of Oxford, and Professor Julia Williams from the University of Hertfordshire. It is funded through a Doctoral Research Fellowship from the National Institute of Health Research.

You will be asked a series of questions about the work you undertake in primary care, and your perceptions of this. This should take about 20 minutes. No other background knowledge is required.

Do I have to take part?

No. Please note that participation is voluntary. If you do decide to take part, you may withdraw at any point for any reason before submitting your answers by pressing the 'Exit' button/ closing the browser. We are offering every paramedic who completes the questionnaire a £10 Amazon e-voucher. However, we are only able to offer this to participants who complete all study activities.

We have included a 'Prefer not to say' option for each set of questions should you prefer not to answer a particular question.

How will my data be used?

During the main set of questions, we will not collect any data that could directly identify you.

At the end of the survey, you will see a page to register your interest in being part of the second phase of our research which will involve an interview. To register your interest, please submit your name and nhs.net email address. This is not linked to your completion of the questionnaire and there is no obligation to be involved in the second phase of our research.

We are offering every paramedic who completes the questionnaire a £10 Amazon e-voucher. At the end of the survey, you will be taken to a separate page to give us your

SURVEY QUESTIONNAIRE

name and nhs.net email address in order to claim this e-voucher. This cannot be matched to your responses in the survey and will be destroyed when individual participant involvement is complete (e.g. when the Amazon e-voucher has been sent and redeemed by you.

Your IP address will not be stored. We will take all reasonable measures to ensure that data remain confidential.

The responses you provide will be stored in a password-protected electronic file on University of Oxford secure servers and may be used in academic publications, conference presentations, reports for external organisations and on websites. Identifiable information will be deleted as soon as it is no longer required for the research. Research data will be stored for 3 years after publication or public release of the study's results.

Who will have access to my data?
The University of Oxford is the data controller with respect to your personal data (name and email address) and, as such, will determine how your personal data is used in the study. The University will process your personal data for the purpose of the research reasons outlined above. Research is a task that we perform in the public interest. Further information about your rights with respect to your personal data is available from https://compliance.admin.ox.ac.uk/individual-rights.

All data will be anonymous. The answers you give us will be shared with the College of Paramedics, Health Education England, and the National Institute for Health Research.

We would also like your permission to use the questionnaire answers in future studies, and to share these with other researchers (e.g., in online databases). This will not include your name and email address, as this is only to claim incentive for the study or to participate in future work associated with this project.

The results will be written up for a DPhil (PhD) degree.

Who has reviewed this study?
This project has been reviewed by, and received ethics clearance through, a subcommittee of the University of Oxford Central University Research Ethics Committee R64129/RE001.

Who do I contact if I have a concern or I wish to complain?
If you have a concern about any aspect of this study, please speak to Georgette Eaton at georgette.eaton@phc.ox.ac.uk or their supervisor, Professor Kamal R. Mahtani kamal.mahtani@phc.ox.ac.uk and we will do our best to answer your query. We will acknowledge your concern within 10 working days and give you an indication of how it will be dealt with. If you remain unhappy or wish to make a formal complaint, please contact the Chair of the Research Ethics Committee at the University of Oxford who will seek to resolve the matter as soon as possible.

Please note that you may only participate in this survey if you are 18 years of age or over	Yes<br>No
If you have read the information above and agree to participate with the understanding that the data (including personal data) you submit will be processed accordingly, please tick the box below to start.	Yes<br>No

SURVEY QUESTIONNAIRE

**Section 1.** The purpose of this section is to find out about the prevalence of paramedics working in primary care in the United Kingdom.	
Are you currently a paramedic working in primary care?	Yes<br>No
What is your age range?	18-24 years old<br>25-34 years old<br>35-44 years old<br>45-54 years old<br>55-65 years old
Which of the following most accurately describe(s) you?<br><br>(Multiple choice answer)	Female<br>Intersex<br>Male<br>Non-binary<br>Transgender<br>Let me type (answer box)
Where do you practice?	England<br>Northern Ireland<br>Scotland<br>Wales
How long have you been a registered paramedic?	0-2 years<br>3-5 years<br>6-10 years<br>11- 14 years<br>15 - 19 years<br>20-24 years<br>25 - 30 years<br>31-35 years<br>>36 years
How long have you worked in your current role in primary care?	0-6 months<br>6-12 months<br>12 – 18months<br>18months – 2 years<br>2-4 years<br>5-7 years<br>8-10 years<br>11- 14 years<br>15 - 19 years<br>>20 years
What is your salary?	Band 5 (£24,907 - £30,615)<br>Band 6 (£31,365 - £37,890)<br>Band 7 (£38,890 - £44,503)<br>Band 8a (£45,753 - £51,668)<br>Band 8b (£53,168 - £62,001)<br>Band 8c (£63,751 - £73,664)<br>Band 8d (£75,914 - £87,754)<br>Band 9 (£91,004 - £104,927)

For peer review only

SURVEY QUESTIONNAIRE

What hours do you work in primary care?	Full time – more than 30 hours per week<br>Part time – 21-30 hours per week<br>Part time – 10-20 hours per week<br>One day a week<br>One session a week (4 hours)
What is your highest qualification relevant to your role as a paramedic in primary care?	Institute of Health and Care Development (IHCD) Graduate Diploma<br>Diploma/Certificate of Higher Education<br>Foundation degree<br>Bachelor's degree<br>Practice Certificate<br>Postgraduate Certificate<br>Postgraduate Diploma<br>Master's Degree<br>Let me type (answer box)
What is your job title?	Paramedic<br>Specialist Paramedic<br>Paramedic Practitioner<br>First Contact Practitioner (Paramedic)<br>Emergency Care Practitioner<br>Advanced Clinical Practitioner (Paramedic)<br>Advanced Paramedic<br>Consultant Paramedic<br>Let me type (answer box)
Are you annotated as an independent prescriber with the Health and Care Professions Council?	Yes<br>Not yet – I am undertaking the course currently<br>No – but I would like to be in the future<br>No
How are you employed in primary care?	Directly employed by GP practice<br>Directly employed by a primary care network/federation/health board<br>Freelance/locum contract<br>Agency/Private provider<br>Rotational through ambulance trust<br>Let me type (answer box)
What type of tasks do you undertake*?<br><br>*These could be face to face or remotely (by telephone or online)<br><br>Please select as many as apply.	Same day/urgent home visits<br>Routine home visits<br>Same day/urgent clinic appointments*<br>Minor illness clinic*<br>Routine clinic appointments*<br>Telephone triage<br>Care home ward rounds<br>Chronic Disease Reviews*<br>Covid clinics*

For peer review only

SURVEY QUESTIONNAIRE

	Admin (e.g. requesting investigations, interpreting results, medication checks)<br>Let me type (answer box)
What types of patients do you see?	Let me type (answer box)
Are there any patient types that you do not see?	Let me type (answer box)
Why?	Let me type (answer box)

For peer review only

SURVEY QUESTIONNAIRE

Section 2.
The purpose of this section is to understand the type of activities that make up your role as a paramedic in primary care. For each of the following activities, please indicate the extent that these form part of your role

	Not at all	Little extent	Some extent	Great extent	Very great extent
Communication and consultations					
Practising holistically to personalise care and promote public and person health					
Working with colleagues in primary care					
Maintaining an ethical approach and fitness to practice					
Information gathering and interpretation					
Clinical Examination and procedural skills					
Making a diagnosis					
Managing medical and clinical complexity					
Independent prescribing, medicines, and supply of pharmacotherapy					
Leadership and Management					
Education and Development					
Research and evidence-based practice					

For peer review only

SURVEY QUESTIONNAIRE

**Section 3.** The purpose of this section is to understand the type of clinical presentations that make up your role as a paramedic in primary care. For each of the following activities, please tick the regularity of which you see these in your role in primary care					
	Not at all	Sometimes	Regularly	Very regularly	All the time
**Cardiovascular system*** Blood pressure issues, chest pain, chest discomfort, orthopnoea, palpitation, irregular pulse, oedema, shortness of breath on exertion					
**Dermatology*** Rash, itching, infestation, spots, skin lesions/moles, nail issues, changes in pigmentation					
**Eyes, Ears, Nose and Throat*** Red eye, visual disturbance, acute loss of vision, eye discharge, eye injury, foreign body, swollen eyelid, dizziness, vertigo, otalgia, otorrhoea, sinus pain, nasal pain, nasal obstruction, mouth pain, neck swelling, sore throat, throat swelling, tinnitus, hearing loss, voice changes					
**Emergency presentations*** Cardiac arrest, catastrophic haemorrhage, shock, respiratory distress, cardiovascular emergency, anaphylaxis, angioedema, allergic reaction, collapse, seizure, non-blanching rash, overdose/poisoning, suspected DKA, meningism, limp child					
**Gastrointestinal system*** Difficulty swallowing, poor appetite, excessive thirst, abdominal pain, abdominal distention, abdominal mass/swelling, constipation, diarrhoea, change in bowel habit, nausea/vomiting, hematemesis, indigestion, rectal bleeding, abdominal blood results (eg. deranged liver enzymes, LFTs, anaemia), high risk behaviours and concerns, stoma issues					

For peer review only

SURVEY QUESTIONNAIRE

**Genitourinary System*** Loin pain, groin pain, haematuria, urinary symptoms, kidney disease, recurrent infection, penile pain, testicular pain/swelling, inability to pass urine, profuse vaginal bleeding, acute groin swelling/pain					
**General presentations*** Breast symptoms, tired all the time, generalised aches and pains, lymphadenopathy, sleep issues, fever, substance/alcohol misuse, overdose/poisoning, vulnerable adult, family/carer concern, genetic predisposition, presentations in patients with a learning disability; review of blood test results					
**Sexual health assessment*** Genital rashes/irritation, urinary symptoms, penile pain, penile discharge, acute groin swelling/pain, pelvic pain/mass, contraception					
**Medication Review*** Adverse side effects, ineffective medication, poor compliance, overuse of medication, misuse of medication, issues with polypharmacy, abnormal blood test results, higher risk groups (risk reduction medications)					
**Mental Health*** Suicidal ideation, self-harm, acute anxiety, stress, panic, post-natal mental health issues, visual/auditory hallucinations, paranoia, bereavement, substance misuse					
**Musculoskeletal system*** Pain, swelling, redness, stiffness, difficulty with movement, minor injury					
**Neurological system*** Altered level of consciousness, fits faints and funny turns, dizziness, altered power tone sensitivity, paraesthesia, altered level of consciousness,					

For peer review only

SURVEY QUESTIONNAIRE

weakness, altered gait, facial palsy, tremor, speech changes, headache, head injury, memory problems, confusion					
**Paediatrics***<br>Vulnerable child, rashes, pyrexia of unknown origin, crying baby, otalgia/otorrhoea, eye injury, red eye/discharge, cough/wheeze/stridor/respiratory distress/nasal symptoms, sore throat, vomiting, diarrhoea, acute bowel symptoms, abdominal pain, constipation, musculoskeletal symptoms, minor injury					
**Pain***<br>Acute pain, worsening pain, change in type of pain					
**Palliative and end of life care***<br>Symptom management, discussions about advanced care planning, verification of death					
**Pregnancy related conditions**					
**Non-pregnancy related conditions in pregnant people**					
**Respiratory System***<br>Shortness of breath, breathing difficulties, pain on breathing, cough (including haemoptysis), wheeze, pallor, cyanosis, suspected or recurrent infection, acute covid-19, post covid-19 syndrome					

For peer review only

SURVEY QUESTIONNAIRE

Section 4.
The purpose of this section is to understand the type of clinical investigations and procedural skills that make up your role as a paramedic in primary care. For each of the following activities, please order the regularity that you undertake these in your role in primary care

	Not at all	Sometimes	Regularly	Very regularly	All the time
Abdominal examination – including inspection, auscultation, percussion & palpation					
Assessment for lymphadenopathy					
Blood pressure					
Blood sugar					
Blood ketones					
Blood tests – FBC, ESR, iron studies, TFT, HbA1c, LFT, U&Es, haematinics, PSA, ACR, B12, drug levels, calcium, CRP, clotting factors, vitamin D, rheumatoid factor, anti CCP, urate, D-dimer, INR, TnT, autoimmune antibodies, TFT, lipid profile, testosterone, SHBG, free androgen index, FSH/LH +/- prolactin, CA125, CA19-9, CRP, coeliac screen, amylase, hepatitis, HIV, glandular fever screen/monospot, BNP/NT-proBNP					
Cardiovascular examination – including inspection, auscultation & palpation, Jugular venous pressure					
Core paramedic skills: Emergency procedures for seeking assistance and calling ambulance; provide basic life support (CPR, defibrillator); administration of nebulised therapies; management of anaphylaxis; management of suspected meningitis; management of seizures; management of					

For peer review only

SURVEY QUESTIONNAIRE

suspected MI; administration of oxygen					
Digital rectal examination					
Interpretation of injection fraction from echocardiogram (Echo)					
Electrocardiograph (ECG)					
Eye examination including inspection and visual acuity, fundoscopy and pupils, fluorescein, local anaesthetic					
Respiratory clinic - FeNO testing Spirometry					
Request for Imaging (eg. X-ray, CT, MRI, Ultrasound of organs, MSK and soft tissue, pelvic transvaginal and testicular)					
Interpretation for imaging					
Joint Injections					
Prescribing					
Mental health examination – Person Health Questionnaire (PHQ9); Generalised Anxiety Disorder Questionnaire (GAD7); Edinburgh Post					
Mid-stream urine culture					
Minor Surgery					
Mini mental state examination (MMSE), GPCOG and 6CIT, 480					
Musculoskeletal examination – including spine, neck, shoulders, elbows, wrists, hands, fingers, hips, pelvis, knee, ankle, feet and toes using Look/Feel/Move principles					
Neurological examination – including inspection, palpation, reflexes, sensation, power, tone, strength, pupils and nystagmus, cranial nerve and cerebellar testing					
Otoscopy					
Oxygen saturations					
Peak Expiratory Flow Rate					
Phlebotomy					

For peer review only

SURVEY QUESTIONNAIRE

Pulse rate, rhythm, volume and character					
Referral to specialism or specialist services					
Respiratory examination – including inspection, auscultation, percussion and palpation					
Respiratory rate					
Skin and/or nail scrapings/samples					
Smear					
Spirometry					
Sputum sample					
Stool sample – culture and sensitivity, faecal calprotectin, helicobacter-pylori testing, FIT testing or FOB					
Swabs					
Temperature					
Throat examination					
Urinalysis and HCG					
Vaginal examination (PV)					
Are there any clinical investigations and procedural skills that you undertake that we have not listed here?		Let me type (answer box)			

For peer review only

SURVEY QUESTIONNAIRE

**Section 5.** The purpose of this section is to understand your perspectives of your role working in primary care. You are welcome to write your answers, or bullet point them – whichever works best for you	
Why did you choose to work in primary care?	
Compared to your previous role, how do you find your current role in primary care?	Prompt: What are the challenges? What are the differences?
Do you experience conflict in your role?	Yes – go to next Q<br>No – skip
What type of conflict occurs?	
Do you receive clinical supervision in your role?	Yes – go to next Q<br>No – skip
To what extent does the clinical supervision/support you receive meet your needs?	
Do you feel patients are satisfied following a consultation with you?	Yes – go to next Q<br>No - go to next Q (negative)
Why do you feel patients are (not) satisfied following a consultation with you?	
How does Health Education England's Roadmap to Practice in primary care impact you in your role?	
What frustrations do you have about your role?	
How does your role as a paramedic in primary care make a difference to:<br>- Patients<br>- Other members of the primary care team<br>- The profession	

For peer review only

Supplemental Table A. Correlations between clinical presentations and demographics of paramedics

			Clinical Supervision	Hours worked	Job Title	Length of time registered as a paramedic	Length of time in primary care	Level of Education	Prescribing Status
Spearman's rho									
Cardiovascular	Blood pressure issues	Correlation Coefficient	.118	.139	.062	.002	.001	.105	.068
		Sig. (2-tailed)	0.029	0.01	0.256	0.972	0.988	0.052	0.21
		N	341	341	341	341	341	341	341
	Chest discomfort	Correlation Coefficient	.097	.065	0.212**	.024	.120	.061	.084
		Sig. (2-tailed)	0.073	0.234	0.0000	0.66	0.027	0.262	0.123
		N	341	341	341	341	341	341	341
	Chest pain	Correlation Coefficient	.016	.018	0.206**	-.002	.103	.076	.050
		Sig. (2-tailed)	0.762	0.737	0.0001	0.972	0.058	0.163	0.358
		N	341	341	341	341	341	341	341
	Irregular pulse	Correlation Coefficient	.087	.176	.036	.061	.088	.047	.057
		Sig. (2-tailed)	0.108	0.001	0.506	0.259	0.105	0.389	0.298
		N	341	341	341	341	341	341	341
	Oedema	Correlation Coefficient	.188	.130*	-.018	-.045	-.037	-.024	.010
		Sig. (2-tailed)	0.0001	0.016	0.742	0.408	0.499	0.659	0.851
		N	341	341	341	341	341	341	341
	Othopnoea	Correlation Coefficient	.058	.106	.096	.030	.053	-.029	.051
		Sig. (2-tailed)	0.283	0.051	0.076	0.584	0.325	0.588	0.349
		N	341	341	341	341	341	341	341
	Palpitations	Correlation Coefficient	.076	.094	.186**	.015	.093	.129	.171
		Sig. (2-tailed)	0.159	0.083	0.0005	0.786	0.086	0.017	0.002
		N	341	341	341	341	341	341	341
	Shortness of breath on exertion	Correlation Coefficient	.140	.177	.028	-.049	.026	.029	.059
		Sig. (2-tailed)	0.01	0.001	0.606	0.37	0.628	0.591	0.278
		N	341	341	341	341	341	341	341
** Correlation is significant at the 0.0008 level (2-tailed).									
Dermatology	Changes in pigmentation	Correlation Coefficient	-.011	.108	.093	.073	.100	.093	.279**
		Sig. (2-tailed)	0.843	0.045	0.087	0.177	0.066	0.087	0.000
		N	341	341	341	341	341	341	341
	Infestation	Correlation Coefficient	.022	.094	.105	.103	.206**	.159**	.365**
		Sig. (2-tailed)	0.688	0.084	0.053	0.058	0.000	0.003	0.000
		N	341	341	341	341	341	341	341
	Itching	Correlation Coefficient	.086	.037	.213**	.094	0.125	.203**	.303**
		Sig. (2-tailed)	0.111	0.500	0.000	0.084	0.021	0.000	0.000
		N	341	341	341	341	341	341	341
	Nail issues	Correlation Coefficient	.019	.093	.098	.108	.165**	.133*	.308**
		Sig. (2-tailed)	0.725	0.087	0.070	0.046	0.002	0.014	0.000
		N	341	341	341	341	341	341	341
	Rash	Correlation Coefficient	.108	-.003	.226**	.048	.063	.192**	.260**
		Sig. (2-tailed)	0.045	0.962	0.000	0.379	0.249	0.000	0.000
		N	341	341	341	341	341	341	341
	Skin lesions/moles	Correlation Coefficient	.113	.044	.182**	.036	.111	.156**	.327**
		Sig. (2-tailed)	0.038	0.420	0.001	0.503	0.041	0.004	0.000
		N	341	341	341	341	341	341	341
	Spots	Correlation Coefficient	.092	.024	.161**	.077	.164**	.188**	.313**
		Sig. (2-tailed)	0.088	0.662	0.003	0.156	0.002	0.000	0.000
		N	341	341	341	341	341	341	341
** Correlation is significant at the 0.001 level (2-tailed).									
Eard, Nose and Throat	Acute loss of vision	Correlation Coefficient	.042	.166	.088	-.004	.249**	.101	.176
		Sig. (2-tailed)	0.443	0.002	0.104	0.948	0.0000	0.063	0.001
		N	341	341	341	341	341	341	341
	Dizziness	Correlation Coefficient	.130	.167	.153	-.049	.089	.131	.147
		Sig. (2-tailed)	0.016	0.002	0.005	0.370	0.100	0.015	0.007
		N	341	341	341	341	341	341	341
	Eye discharge	Correlation Coefficient	.093	.047	.156**	.067	.197**	.151	.215**
		Sig. (2-tailed)	0.088	0.382	0.004	0.216	0.0002	0.005	0.0001
		N	341	341	341	341	341	341	341
	Eye injury	Correlation Coefficient	-.053	.068	.108*	.020	.140	.013	.046
		Sig. (2-tailed)	0.327	0.209	0.046	0.711	0.010	0.816	0.395
		N	341	341	341	341	341	341	341
	Foreign body	Correlation Coefficient	-.025	.020	.068	.021	.143	.024	.063
		Sig. (2-tailed)	0.645	0.719	0.208	0.706	0.008	0.664	0.245
		N	341	341	341	341	341	341	341
	Hearing loss	Correlation Coefficient	.084	.192	.071	.011	.149**	.091	.308**
		Sig. (2-tailed)	0.122	0.0004	0.188	0.834	0.006	0.095	0.0000
		N	341	341	341	341	341	341	341
	Mouth pain	Correlation Coefficient	.043	.080	.014	-.036	.092	.066	.181
		Sig. (2-tailed)	0.431	0.140	0.797	0.507	0.089	0.222	0.001
		N	341	341	341	341	341	341	341
	Nasal obstruction	Correlation Coefficient	-.060	.159	.093	.012	.212**	.134	.267**
		Sig. (2-tailed)	0.272	0.003	0.088	0.821	0.0001	0.013	0.0000
		N	341	341	341	341	341	341	341
	Nasal pain	Correlation Coefficient	.071	.143	.124	.030	.149	.160	.324**
		Sig. (2-tailed)	0.194	0.008	0.022	0.584	0.006	0.003	0.0000
		N	341	341	341	341	341	341	341
	Neck swelling	Correlation Coefficient	.057	.098	.139	.004	.119	.198**	.225**
		Sig. (2-tailed)	0.293	0.072	0.010	0.948	0.028	0.0002	0.0000
		N	341	341	341	341	341	341	341
	Otalgia	Correlation Coefficient	.104	.009	.243**	.099	.121	.128	.214**
		Sig. (2-tailed)	0.056	0.865	0.0000	0.069	0.026	0.018	0.0001
		N	341	341	341	341	341	341	341
		Correlation Coefficient	.120	.056	.210**	.069	.171	.134	.259**

For peer review only

Eyes, E	Otorrhoea	Sig. (2-tailed)	0.027	0.305	0.0001	0.206	0.002	0.013	0.0000
		N	341	341	341	341	341	341	341
	Red eye	Correlation Coefficient	.098	.069	.141	-.004	.153	.130	.189
		Sig. (2-tailed)	0.070	0.201	0.009	0.943	0.005	0.016	0.0005
		N	341	341	341	341	341	341	341
	Sinus pain	Correlation Coefficient	.107	.137	.202**	.075	.164	.200**	.307**
		Sig. (2-tailed)	0.048	0.011	0.0002	0.167	0.002	0.0002	0.0000
		N	341	341	341	341	341	341	341
	Sore throat	Correlation Coefficient	.078	.072	.210**	.043	.083	.171	.134
		Sig. (2-tailed)	0.148	0.183	0.0001	0.430	0.127	0.002	0.013
		N	341	341	341	341	341	341	341
	Swollen eyelid	Correlation Coefficient	.090	.099	.146	.056	.146	.151	.259**
		Sig. (2-tailed)	0.098	0.069	0.007	0.303	0.007	0.005	0.0000
		N	341	341	341	341	341	341	341
	Throat swelling	Correlation Coefficient	.003	.101	.140	-.096	.132	.161	.192
		Sig. (2-tailed)	0.955	0.063	0.010	0.079	0.015	0.003	0.0004
		N	341	341	341	341	341	341	341
	Tinnitus	Correlation Coefficient	.097	.066	.119	.040	.101	.195**	.215**
		Sig. (2-tailed)	0.074	0.224	0.029	0.459	0.062	0.0003	0.0001
		N	341	341	341	341	341	341	341
	Vertigo	Correlation Coefficient	.111	.121	.116	-.004	.078	.181	.176
		Sig. (2-tailed)	0.040	0.025	0.032	0.937	0.151	0.001	0.001
		N	341	341	341	341	341	341	341
	Visual disturbance	Correlation Coefficient	.085	.111	.103	.005	.168	.106	.191
		Sig. (2-tailed)	0.119	0.042	0.058	0.934	0.002	0.051	0.0004
		N	341	341	341	341	341	341	341
	Voice changes	Correlation Coefficient	.034	.099	.047	.015	.216**	.090	.274**
		Sig. (2-tailed)	0.535	0.067	0.389	0.777	0.0001	0.097	0.0000
		N	341	341	341	341	341	341	341

** Correlation is significant at the 0.0003 level (2-tailed).

Female and Male Anatamoical Health	Acute groin swelling/pain	Correlation Coefficient	.038	.140	.202**	-.017	.184**	.206**	.276**
		Sig. (2-tailed)	0.487	0.010	0.0002	0.754	0.001	0.0001	0.00000
		N	341	341	341	341	341	341	341
	Breast symptoms	Correlation Coefficient	-.024	-.038	.138	-.018	.087	.169	.191**
		Sig. (2-tailed)	0.664	0.483	0.011	0.735	0.109	0.002	0.0004
		N	341	341	341	341	341	341	341
	Genital rashes/irritation	Correlation Coefficient	-.024	.094	.239**	.022	.145	.188**	.332**
		Sig. (2-tailed)	0.662	0.082	0.00001	0.688	0.007	0.0005	0.00000
		N	341	341	341	341	341	341	341
	Pelvic pain/mass	Correlation Coefficient	-.024	.136	.167	.050	.164	.169	.242**
		Sig. (2-tailed)	0.665	0.012	0.002	0.361	0.002	0.002	0.00001
		N	341	341	341	341	341	341	341
	Penile discharge	Correlation Coefficient	.032	.133	.137	.037	.158	.195**	.293**
		Sig. (2-tailed)	0.560	0.014	0.011	0.499	0.003	0.0003	0.00000
		N	341	341	341	341	341	341	341
	Penile pain	Correlation Coefficient	.039	.147	.200**	-.027	.170	.234**	.331**
		Sig. (2-tailed)	0.469	0.007	0.0002	0.614	0.002	0.00001	0.00000
		N	341	341	341	341	341	341	341
	Urinary symptoms	Correlation Coefficient	.042	.036	.174**	-.003	.013	.060	.134
		Sig. (2-tailed)	0.439	0.513	0.001	0.949	0.804	0.273	0.013
		N	341	341	341	341	341	341	341

** Correlation is significant at the 0.001 level (2-tailed).

Gastrointestinal & Hepatic System	Abdominal blood results	Correlation Coefficient	.187	.131	.117	.033	.065	.142	.224**
		Sig. (2-tailed)	0.001	0.015	0.031	0.538	0.229	0.009	0.0000
		N	341	341	341	341	341	341	341
	Abdominal distention	Correlation Coefficient	.041	.249**	.042	-.071	.085	.018	.012
		Sig. (2-tailed)	0.455	0.0000	0.440	0.188	0.116	0.737	0.820
		N	341	341	341	341	341	341	341
	Abdominal mass/swelling	Correlation Coefficient	.038	.207**	.023	-.041	.096	.009	.059
		Sig. (2-tailed)	0.489	0.0001	0.671	0.451	0.076	0.872	0.274
		N	341	341	341	341	341	341	341
	Abdominal pain	Correlation Coefficient	.060	.023	.133	.001	.045	.113	.093
		Sig. (2-tailed)	0.273	0.678	0.014	0.991	0.410	0.037	0.085
		N	341	341	341	341	341	341	341
	Change in bowel habit	Correlation Coefficient	.064	.004	.084	-.041	.002	.091	.144
		Sig. (2-tailed)	0.238	0.943	0.120	0.451	0.973	0.094	0.008
		N	341	341	341	341	341	341	341
	Constipation	Correlation Coefficient	.102	.134	.072	-.014	.013	.041	.115
		Sig. (2-tailed)	0.060	0.013	0.187	0.791	0.813	0.448	0.034
		N	341	341	341	341	341	341	341
	Diarrhoea	Correlation Coefficient	.042	.094	.064	-.039	.052	.041	.116
		Sig. (2-tailed)	0.434	0.082	0.239	0.477	0.338	0.451	0.032
		N	341	341	341	341	341	341	341
	Difficulty swallowing	Correlation Coefficient	.070	.152	.079	-.007	.145	.026	.184
		Sig. (2-tailed)	0.195	0.005	0.144	0.902	0.007	0.627	0.001
		N	341	341	341	341	341	341	341
	Excessive thirst	Correlation Coefficient	.014	.183	.076	-.056	.036	.077	.174
		Sig. (2-tailed)	0.797	0.001	0.161	0.300	0.507	0.158	0.001
		N	341	341	341	341	341	341	341
	Hematemesis	Correlation Coefficient	-.012	.103	.098	-.019	.143	.116	.133
		Sig. (2-tailed)	0.820	0.058	0.070	0.732	0.008	0.032	0.014
		N	341	341	341	341	341	341	341
	High risk behaviours and	Correlation Coefficient	-.017	.143	.016	.121	.138	-.011	.086
		Sig. (2-tailed)	0.750	0.008	0.768	0.025	0.011	0.836	0.114

	concerns	N	341	341	341	341	341	341	341
	Indigestion	Correlation Coefficient	.142	.097	.222**	.008	.138	.157	.262**
		Sig. (2-tailed)	0.009	0.073	0.0000	0.883	0.011	0.004	0.0000
		N	341	341	341	341	341	341	341
	Nausea/vomiting	Correlation Coefficient	.138	.102	.123	.005	.055	.078	.159
		Sig. (2-tailed)	0.011	0.060	0.023	0.929	0.308	0.152	0.003
		N	341	341	341	341	341	341	341
	Poor appetite	Correlation Coefficient	.090	.098	.034	.043	.045	-.001	.070
		Sig. (2-tailed)	0.095	0.070	0.530	0.430	0.407	0.978	0.197
		N	341	341	341	341	341	341	341
	Rectal bleeding	Correlation Coefficient	.074	.128	.153	.012	.117	.119	.242**
		Sig. (2-tailed)	0.174	0.018	0.005	0.820	0.031	0.028	0.0000
		N	341	341	341	341	341	341	341
	Stoma issues	Correlation Coefficient	-.033	.242**	-.017	.047	.107	-.065	.130
		Sig. (2-tailed)	0.544	0.0000	0.754	0.383	0.049	0.235	0.016
		N	341	341	341	341	341	341	341
** Correlation is significant at the 0.0004 level (2-tailed).									
Key Presentations	Fever	Correlation Coefficient	.038	.070	.150	.000	.044	.101	.127
		Sig. (2-tailed)	0.486	0.200	0.006	0.999	0.415	0.063	0.019
		N	341	341	341	341	341	341	341
	Generalised aches and pains	Correlation Coefficient	.121	.052	.069	-.037	-.001	.064	.081
		Sig. (2-tailed)	0.026	0.335	0.203	0.493	0.988	0.238	0.134
		N	341	341	341	341	341	341	341
	Genetic predisposition	Correlation Coefficient	.029	.029	.036	.049	.069	.043	.027
		Sig. (2-tailed)	0.593	0.590	0.504	0.365	0.204	0.424	0.617
		N	341	341	341	341	341	341	341
	Lymphadenopathy	Correlation Coefficient	.138	.136	.125	0.054	0.057	.149	.228**
		Sig. (2-tailed)	0.011	0.012	0.021	0.318	0.298	0.006	0.00002
		N	341	341	341	341	341	341	341
	Overdose/ poisoning	Correlation Coefficient	-.048	.254**	.101	-.073	.160	.057	.074
		Sig. (2-tailed)	0.377	0.00000	0.061	0.181	0.003	0.290	0.174
		N	341	341	341	341	341	341	341
	Presentations in patients with a learning disability	Correlation Coefficient	.024	.178	.075	.097	.117	.029	.063
		Sig. (2-tailed)	0.653	0.001	0.166	0.075	0.031	0.588	0.249
		N	341	341	341	341	341	341	341
	Review of blood test results	Correlation Coefficient	.144	.094	.201	-.001	.087	.199	.282**
		Sig. (2-tailed)	0.008	0.082	0.0002	0.982	0.109	0.0002	0.00000
		N	341	341	341	341	341	341	341
	Sleep issues	Correlation Coefficient	.074	.060	.040	-.104	-.039	.080	.107
		Sig. (2-tailed)	0.171	0.267	0.457	0.056	0.472	0.140	0.048
		N	341	341	341	341	341	341	341
	Substance/alcohol misuse	Correlation Coefficient	.014	.127	.115	-.018	.175	.092	.092
		Sig. (2-tailed)	0.791	0.019	0.034	0.743	0.001	0.091	0.090
		N	341	341	341	341	341	341	341
	Tired all the time	Correlation Coefficient	.165	.077	.096	-.036	.029	.105	.160
		Sig. (2-tailed)	0.002	0.154	0.077	0.513	0.590	0.053	0.003
		N	341	341	341	341	341	341	341
	Vulnerable adult, family/carer concern	Correlation Coefficient	.072	.216	-.033	.016	.054	-.069	-.013
		Sig. (2-tailed)	0.185	0.0001	0.545	0.765	0.320	0.205	0.807
		N	341	341	341	341	341	341	341
** Correlation is significant at the 0.0004 level (2-tailed).									
Medication Review and Issues	Abnormal blood test results	Correlation Coefficient	.105	.098	.194**	.055	.095	.186	.264**
		Sig. (2-tailed)	0.052	0.072	0.0003	0.312	0.080	0.001	0.00000
		N	341	341	341	341	341	341	341
	Adverse side effects of medication	Correlation Coefficient	.026	.080	.118	.046	.222**	.131	.255**
		Sig. (2-tailed)	0.626	0.142	0.029	0.402	0.00003	0.016	0.00000
		N	341	341	341	341	341	341	341
	Higher risk groups (risk reduction medications)	Correlation Coefficient	.044	.116	.159	.078	.206**	.145	.235**
		Sig. (2-tailed)	0.416	0.033	0.003	0.152	0.0001	0.007	0.00001
		N	341	341	341	341	341	341	341
	Ineffective medication	Correlation Coefficient	.015	.085	.145	.044	.149	.139	.274**
		Sig. (2-tailed)	0.776	0.117	0.007	0.420	0.006	0.010	0.00000
		N	341	341	341	341	341	341	341
	Issues with polypharmacy	Correlation Coefficient	.038	.145	.187	.120	.195**	.163	.302**
		Sig. (2-tailed)	0.488	0.007	0.001	0.026	0.0003	0.002	0.00000
		N	341	341	341	341	341	341	341
	Misuse of medication	Correlation Coefficient	-.005	.130	.156	.101	.188**	.128	.203
		Sig. (2-tailed)	0.929	0.017	0.004	0.063	0.0005	0.018	0.0002
		N	341	341	341	341	341	341	341
	Overuse of medication	Correlation Coefficient	.041	.083	.230**	.099	.197**	.122	.243**
		Sig. (2-tailed)	0.451	0.127	0.00002	0.068	0.0002	0.024	0.00001
		N	341	341	341	341	341	341	341
	Poor compliance with medication	Correlation Coefficient	.071	.105	.135	.103	.144	.110	.191**
		Sig. (2-tailed)	0.188	0.052	0.012	0.058	0.008	0.042	0.0004
		N	341	341	341	341	341	341	341
** Correlation is significant at the 0.0008 level (2-tailed).									
	Difficulty with movement	Correlation Coefficient	.054	.104	.113	.036	.023	.023	.072
		Sig. (2-tailed)	0.317	0.055	0.037	0.508	0.669	0.668	0.186
		N	341	341	341	341	341	341	341
	Minor injury	Correlation Coefficient	-.007	.082	.061	-.049	.040	-.020	-.023
		Sig. (2-tailed)	0.902	0.129	0.265	0.369	0.467	0.709	0.669
		N	341	341	341	341	341	341	341

For peer review only

Musculoskeletal	Pain	Correlation Coefficient	.057	.051	.153	.070	.019	.063	.039
		Sig. (2-tailed)	0.296	0.346	0.005	0.198	0.725	0.249	0.472
		N	341	341	341	341	341	341	341
	Redness	Correlation Coefficient	.051	.164	.146	.031	.092	.006	.093
		Sig. (2-tailed)	0.350	0.002	0.007	0.569	0.090	0.907	0.086
		N	341	341	341	341	341	341	341
	Stiffness	Correlation Coefficient	.064	.100	.128	.040	.062	.042	.112
		Sig. (2-tailed)	0.240	0.064	0.018	0.456	0.253	0.442	0.039
		N	341	341	341	341	341	341	341
	Swelling	Correlation Coefficient	.033	.133	.152	.027	.093	.023	.077
		Sig. (2-tailed)	0.540	0.014	0.005	0.617	0.087	0.679	0.155
		N	341	341	341	341	341	341	341
** Correlation is significant at the 0.001 level (2-tailed).									
Neurological	Altered gait	Correlation Coefficient	.062	.165	.018	.095	.075	.019	.053
		Sig. (2-tailed)	0.251	0.002	0.743	0.081	0.169	0.729	0.326
		N	341	341	341	341	341	341	341
	Altered level of consciousness	Correlation Coefficient	.024	.170	.054	.081	.156	-.012	.036
		Sig. (2-tailed)	0.656	0.002	0.320	0.136	0.004	0.820	0.508
		N	341	341	341	341	341	341	341
	Altered level of consciousness	Correlation Coefficient	.013	.176	.039	.068	.149	-.022	.064
		Sig. (2-tailed)	0.812	0.001	0.472	0.209	0.006	0.687	0.239
		N	341	341	341	341	341	341	341
	Altered power, tone or sensitivity	Correlation Coefficient	.039	.120*	.119	.044	.101	.096	.065
		Sig. (2-tailed)	0.473	0.027	0.028	0.423	0.062	0.076	0.231
		N	341	341	341	341	341	341	341
	Confusion	Correlation Coefficient	.071	.229**	.012	.070	.116	.017	.128
		Sig. (2-tailed)	0.190	0.00002	0.827	0.196	0.032	0.753	0.018
		N	341	341	341	341	341	341	341
	Dizziness	Correlation Coefficient	.101	.061	.125	.004	.042	.144	.092
		Sig. (2-tailed)	0.061	0.258	0.021	0.937	0.436	0.008	0.090
		N	341	341	341	341	341	341	341
	Facial palsy	Correlation Coefficient	.017	.131	.080	.086	.185	.051	.184
		Sig. (2-tailed)	0.754	0.015	0.138	0.112	0.001	0.347	0.001
		N	341	341	341	341	341	341	341
	Fits faints and funny turns	Correlation Coefficient	.096	.110	.118	-.016	.118	.069	.060
		Sig. (2-tailed)	0.078	0.043	0.030	0.762	0.029	0.204	0.268
		N	341	341	341	341	341	341	341
	Head injury	Correlation Coefficient	-.028	.029	.092	-.053	.068	.037	.001
		Sig. (2-tailed)	0.605	0.593	0.088	0.330	0.207	0.498	0.989
		N	341	341	341	341	341	341	341
	Headache	Correlation Coefficient	.103	.046	.195	-.046	.025	.172	.153
		Sig. (2-tailed)	0.057	0.396	0.0003	0.396	0.643	0.001	0.005
		N	341	341	341	341	341	341	341
	Memory problems	Correlation Coefficient	.090	.178	-.028	.075	.084	.032	.130
		Sig. (2-tailed)	0.098	0.001	0.607	0.166	0.120	0.553	0.016
		N	341	341	341	341	341	341	341
	Paraesthesia	Correlation Coefficient	.017	.081	.189	.057	.211	.139	.199
		Sig. (2-tailed)	0.758	0.138	0.0005	0.298	0.0001	0.010	0.0002
		N	341	341	341	341	341	341	341
	Speech changes	Correlation Coefficient	.031	.224**	.011	.049	.184	.014	.097
		Sig. (2-tailed)	0.567	0.00003	0.841	0.367	0.001	0.797	0.073
		N	341	341	341	341	341	341	341
	Tremor	Correlation Coefficient	.039	.127	.031	.013	.134	.042	.099
		Sig. (2-tailed)	0.474	0.019	0.568	0.813	0.014	0.440	0.067
		N	341	341	341	341	341	341	341
	Weakness	Correlation Coefficient	.104	.133	.068	.025	.050	.032	.030
		Sig. (2-tailed)	0.054	0.014	0.210	0.650	0.361	0.550	0.581
		N	341	341	341	341	341	341	341
** Correlation is significant at the 0.0004 level (2-tailed).									
	Abdominal pain	Correlation Coefficient	341	341	341	341	341	341	341
		Sig. (2-tailed)	0.334	0.977	0.00000	0.811	0.002	0.00000	0.00000
		N	.053	-.002	.293**	.013	.171	.249**	.260**
	Acute bowel symptoms	Correlation Coefficient	.040	.014	.254**	-.058	.144	.217**	.234**
		Sig. (2-tailed)	0.461	0.800	0.00000	0.285	0.008	0.00005	0.00001
		N	341	341	341	341	341	341	341
	Constipation	Correlation Coefficient	.049	.052	.266**	.001	.134	.248**	.294**
		Sig. (2-tailed)	0.371	0.335	0.00000	0.987	0.013	0.00000	0.00000
		N	341	341	341	341	341	341	341
	Cough/wheeze/ stridor/respiratory distress/nasal symptoms	Correlation Coefficient	.043	-.032	.249**	.005	.109	.203	.148
		Sig. (2-tailed)	0.433	0.557	0.00000	0.923	0.045	0.0002	0.006
		N	341	341	341	341	341	341	341
	Crying baby	Correlation Coefficient	-.025	-.048	.296**	-.069	.149	.214	.192
		Sig. (2-tailed)	0.639	0.372	0.00000	0.201	0.006	0.0001	0.0004
		N	341	341	341	341	341	341	341
	Diarrhoea	Correlation Coefficient	.059	.009	.246**	-.046	.149	.237**	.230**
		Sig. (2-tailed)	0.280	0.866	0.00000	0.393	0.006	0.00001	0.00002
		N	341	341	341	341	341	341	341
	Eye injury	Correlation Coefficient	.003	.061	.116	-.042	.197	.117	.146
		Sig. (2-tailed)	0.961	0.260	0.032	0.434	0.0002	0.030	0.007
		N	341	341	341	341	341	341	341

For peer review only

Paediatrics	Minor injury	Correlation Coefficient	-.074	-.030	.155	-.039	.190	.081	.092
		Sig. (2-tailed)	0.174	0.583	0.004	0.472	0.0004	0.137	0.090
		N	341	341	341	341	341	341	341
	Musculoskeletal symptoms	Correlation Coefficient	-.031	-.024	.247**	.015	.173	.158	.167
		Sig. (2-tailed)	0.572	0.660	0.00000	0.778	0.001	0.003	0.002
		N	341	341	341	341	341	341	341
	Otalgia/otorrhoea	Correlation Coefficient	.070	-.024	.273**	-.010	.139*	.190	.223**
		Sig. (2-tailed)	0.196	0.663	0.00000	0.851	0.010	0.0004	0.00003
		N	341	341	341	341	341	341	341
	Pyrexia of unknown origin	Correlation Coefficient	.004	-.063	.265**	-.048	.090	.225**	.145
		Sig. (2-tailed)	0.947	0.248	0.00000	0.372	0.097	0.00003	0.007
		N	341	341	341	341	341	341	341
	Rashes	Correlation Coefficient	.023	-.039	.261**	-.007	.142	.208	.238**
		Sig. (2-tailed)	0.671	0.472	0.00000	0.904	0.009	0.0001	0.00001
		N	341	341	341	341	341	341	341
	Red eye/discharge	Correlation Coefficient	.045	.035	.222**	.026	.212	.193	.253**
		Sig. (2-tailed)	0.405	0.517	0.00003	0.628	0.0001	0.0003	0.00000
		N	341	341	341	341	341	341	341
	Sore throat	Correlation Coefficient	.053	-.020	.270**	.003	.099	.224**	.185
		Sig. (2-tailed)	0.327	0.713	0.00000	0.962	0.067	0.00003	0.001
		N	341	341	341	341	341	341	341
	Vomiting	Correlation Coefficient	.066	.008	.262**	-.053	.149	.224**	.225**
		Sig. (2-tailed)	0.227	0.885	0.00000	0.333	0.006	0.00003	0.00003
		N	341	341	341	341	341	341	341
	Vulnerable child	Correlation Coefficient	-.059	.103	.200**	.022	.175	.159	.196**
		Sig. (2-tailed)	0.279	0.057	0.0002	0.686	0.001	0.003	0.0003
		N	341	341	341	341	341	341	341

** Correlation is significant at the 0.0004 level (2-tailed).

Pain	Acute pain	Correlation Coefficient	.100	.071	.160	.053	.096	.127	.122
		Sig. (2-tailed)	0.066	0.190	0.003	0.326	0.076	0.019	0.024
		N	341	341	341	341	341	341	341
	Change in type of pain	Correlation Coefficient	.028	.079	.121	.048	.079	.017	.133
		Sig. (2-tailed)	0.606	0.145	0.026	0.373	0.148	0.750	0.014
		N	341	341	341	341	341	341	341
	Worsening pain	Correlation Coefficient	.052	.079	.140	.046	.035	.050	.090
		Sig. (2-tailed)	0.337	0.144	0.010	0.398	0.523	0.359	0.097
		N	341	341	341	341	341	341	341

** Correlation is significant at the 0.002 level (2-tailed).

Palliative and End of Life Care	Discussions about advanced care planning	Correlation Coefficient	.047	.205**	-.093	.051	.168	-.043	.071
		Sig. (2-tailed)	0.382	0.0001	0.088	0.349	0.002	0.433	0.190
		N	341	341	341	341	341	341	341
	Symptom management	Correlation Coefficient	.056	.205**	-.046	.051	.089	-.029	.044
		Sig. (2-tailed)	0.300	0.0001	0.399	0.349	0.103	0.593	0.418
		N	341	341	341	341	341	341	341

** Correlation is significant at the 0.003 level (2-tailed).

Renal and Genitourinary	Acute groin swelling/pain	Correlation Coefficient	.017	.155	.175	.076	.174	.141	.259**
		Sig. (2-tailed)	0.750	0.004	0.001	0.161	0.001	0.009	0.0000
		N	341	341	341	341	341	341	341
	Groin pain	Correlation Coefficient	.104	.135	.184	-.064	.110	.126	.260**
		Sig. (2-tailed)	0.055	0.012	0.001	0.238	0.041	0.020	0.0000
		N	341	341	341	341	341	341	341
	Haematuria	Correlation Coefficient	.132	.134	.156	.075	.116	.111	.226**
		Sig. (2-tailed)	0.015	0.014	0.004	0.165	0.032	0.041	0.0000
		N	341	341	341	341	341	341	341
	Inability to pass urine	Correlation Coefficient	.048	.206**	.046	.039	.131	.064	.101
		Sig. (2-tailed)	0.378	0.0001	0.400	0.471	0.016	0.240	0.063
		N	341	341	341	341	341	341	341
	Kidney disease	Correlation Coefficient	.090	.209**	.127	.028	.074	.119	.204**
		Sig. (2-tailed)	0.099	0.0001	0.019	0.601	0.173	0.028	0.0001
		N	341	341	341	341	341	341	341
	Loin pain	Correlation Coefficient	.093	.109	.189**	.027	.143	.143	.266**
		Sig. (2-tailed)	0.086	0.044	0.0004	0.622	0.008	0.008	0.0000
		N	341	341	341	341	341	341	341
	Penile pain	Correlation Coefficient	.037	.151	.095	.003	.162	.097	.271**
		Sig. (2-tailed)	0.495	0.005	0.079	0.949	0.003	0.073	0.0000
		N	341	341	341	341	341	341	341
	Profuse vaginal bleeding	Correlation Coefficient	-.044	-.006	.153	.067	.157	.117	.130
		Sig. (2-tailed)	0.416	0.917	0.005	0.218	0.004	0.031	0.017
		N	341	341	341	341	341	341	341
	Recurrent infection	Correlation Coefficient	.100	.141	.055	.058	-.016	.053	.081
		Sig. (2-tailed)	0.065	0.009	0.313	0.283	0.762	0.332	0.134
		N	341	341	341	341	341	341	341
	Testicular pain/swelling	Correlation Coefficient	.017	.129	.211**	.035	.215**	.182	.351**
		Sig. (2-tailed)	0.755	0.017	0.0001	0.517	0.0001	0.001	0.0000
		N	341	341	341	341	341	341	341
	Urinary symptoms	Correlation Coefficient	.136	.058	.089	.022	-.073	.060	.097
		Sig. (2-tailed)	0.012	0.282	0.102	0.691	0.181	0.266	0.074
		N	341	341	341	341	341	341	341

** Correlation is significant at the 0.0006 level (2-tailed).

	Acute Covid-19	Correlation Coefficient	.005	.121	.125	-.079	.029	.063	.069
		Sig. (2-tailed)	0.931	0.026	0.021	0.148	0.588	0.248	0.202
		N	341	341	341	341	341	341	341
	Breathing	Correlation Coefficient	.069	.111	.114	.023	.105	.097	.129

For peer review only

Respiratory	Breathing difficulties	Sig. (2-tailed)	0.202	0.041	0.036	0.678	0.052	0.074	0.017
		N	341	341	341	341	341	341	341
	Cough (including haemoptysis)	Correlation Coefficient	.090	.129	.133	-.005	.054	.126	.140
		Sig. (2-tailed)	0.097	0.017	0.014	0.934	0.321	0.020	0.010
		N	341	341	341	341	341	341	341
	Cyanosis	Correlation Coefficient	-.054	.143	.058	.021	.144	.068	.125
		Sig. (2-tailed)	0.317	0.008	0.284	0.693	0.008	0.213	0.021
		N	341	341	341	341	341	341	341
	Pain on breathing	Correlation Coefficient	.107	.104	.118	-.009	.088	.107	.106
		Sig. (2-tailed)	0.047	0.055	0.030	0.872	0.105	0.048	0.051
		N	341	341	341	341	341	341	341
	Pallor	Correlation Coefficient	-.038	.174	.060	.064	.120	.026	.108
		Sig. (2-tailed)	0.482	0.001	0.272	0.242	0.026	0.638	0.046
		N	341	341	341	341	341	341	341
	Post Covid-19 syndrome	Correlation Coefficient	-.028	.090	.042	-.062	.034	.072	.093
		Sig. (2-tailed)	0.603	0.097	0.435	0.252	0.535	0.186	0.087
		N	341	341	341	341	341	341	341
	Shortness of breath	Correlation Coefficient	.064	.126	.082	-.011	.067	.076	.107
		Sig. (2-tailed)	0.241	0.020	0.130	0.833	0.218	0.162	0.048
		N	341	341	341	341	341	341	341
	Suspected or recurrent infection	Correlation Coefficient	.077	.080	.090	.035	-.015	.100	.052
		Sig. (2-tailed)	0.154	0.138	0.097	0.515	0.789	0.064	0.342
		N	341	341	341	341	341	341	341
	Wheeze	Correlation Coefficient	.086	.128	.126	.046	.100	.121	.146
		Sig. (2-tailed)	0.113	0.018	0.020	0.393	0.066	0.026	0.007
		N	341	341	341	341	341	341	341

** Correlation is significant at the 0.0007 level (2-tailed).

For peer review only

Supplemental Table B. Correlations between clinical examinations and demogaphics of paramedics

			Clinical Supervision	Hours worked	Job Title	Length of time in primary care	Level of Education	Prescribing Status
Spearman's rho	Abdominal examination	Correlation Coefficient	.062	.116	.129	.091	.113	.117
		Sig. (2-tailed)	0.254	0.032	0.017	0.094	0.036	0.031
		N	341	341	341	341	341	341
	Assessment for lymphadenopathy	Correlation Coefficient	.062	.019	.186	.148	.211**	.257**
		Sig. (2-tailed)	0.254	0.730	0.001	0.006	0.0001	0.0000
		N	341	341	341	341	341	341
	Blood pressure	Correlation Coefficient	.064	.094	.072	.058	.071	.017
		Sig. (2-tailed)	0.238	0.085	0.183	0.284	0.190	0.751
		N	341	341	341	341	341	341
	Cardiovascular examination	Correlation Coefficient	.081	.113	.111	.065	.087	.066
		Sig. (2-tailed)	0.138	0.037	0.040	0.230	0.109	0.226
		N	341	341	341	341	341	341
	Cranial nerve examination	Correlation Coefficient	.048	.088	.166	.139	.155	.132
		Sig. (2-tailed)	0.376	0.106	0.002	0.010	0.004	0.015
		N	341	341	341	341	341	341
	Digital rectal examination	Correlation Coefficient	.007	.141	.217**	.195	.264**	.444**
		Sig. (2-tailed)	0.902	0.009	0.0001	0.0003	0.0000	0.0000
		N	341	341	341	341	341	341
	Echocardiogram (Echo)	Correlation Coefficient	-.066	.065	.052	.082	.091	.138
		Sig. (2-tailed)	0.228	0.236	0.343	0.133	0.096	0.011
		N	339	339	339	339	339	339
	Electrocardiograph (ECG)	Correlation Coefficient	-.047	.150	.053	.127	.027	.055
		Sig. (2-tailed)	0.388	0.005	0.329	0.019	0.616	0.311
		N	341	341	341	341	341	341
	Eye examination	Correlation Coefficient	-.036	.017	.105	.204**	.212**	.255**
		Sig. (2-tailed)	0.513	0.753	0.054	0.0002	0.0001	0.000
		N	341	341	341	341	341	341
	FeNO testing	Correlation Coefficient	-.193	.015	.026	.060	.091	.121
		Sig. (2-tailed)	0.0003	0.790	0.632	0.271	0.094	0.025
		N	341	341	341	341	341	341
	Imaging	Correlation Coefficient	-.005	.084	.214**	.129	.127	.185
		Sig. (2-tailed)	0.924	0.124	0.0001	0.018	0.019	0.001
		N	341	341	341	341	341	341
	Jugular venous pressure	Correlation Coefficient	.077	.062	.089	.124	.137	.181
		Sig. (2-tailed)	0.155	0.256	0.099	0.022	0.011	0.001
		N	341	341	341	341	341	341
	Mental health examination	Correlation Coefficient	.092	.037	.073	.126	.129	.178
		Sig. (2-tailed)	0.089	0.496	0.177	0.020	0.017	0.001
		N	341	341	341	341	341	341
	Mid-stream urine culture	Correlation Coefficient	.113	.179	0.075	0.013	0.093	.129
		Sig. (2-tailed)	0.037	0.001	0.167	0.807	0.088	0.017
		N	341	341	341	341	341	341
	Mini mental state examination (MMSE)	Correlation Coefficient	.094	.126	.040	.128	.081	.084
		Sig. (2-tailed)	0.082	0.020	0.464	0.018	0.135	0.120
		N	341	341	341	341	341	341
	Musculoskeletal examinatio	Correlation Coefficient	.017	.086	.153	.133	.112	.073
		Sig. (2-tailed)	0.758	0.113	0.005	0.014	0.039	0.180
		N	340	340	340	340	340	340
	Neurological examination	Correlation Coefficient	.008	.084	.125	.067	.124	.052
		Sig. (2-tailed)	0.887	0.123	0.021	0.217	0.022	0.335
		N	341	341	341	341	341	341
	Otoscopy	Correlation Coefficient	.058	.053	.162	.088	.088	.193
		Sig. (2-tailed)	0.282	0.332	0.003	0.106	0.104	0.0003
		N	341	341	341	341	341	341
	Oxygen saturations	Correlation Coefficient	.048	.080	.029	.029	-.067	-.105
		Sig. (2-tailed)	0.372	0.142	0.593	0.599	0.217	0.052
		N	341	341	341	341	341	341
	Peak Expiratory Flow Rate	Correlation Coefficient	-.006	.045	.036	.183	.037	.123
		Sig. (2-tailed)	0.909	0.407	0.510	0.001	0.491	0.023
		N	341	341	341	341	341	341
	Phlebotomy	Correlation Coefficient	.016	.220**	-.105	-.085	-.047	.033
		Sig. (2-tailed)	0.762	0.0000	0.053	0.116	0.385	0.539

For peer review only

	N	341	341	341	341	341	341
Pulse rate, rhythm, volume and character	Correlation Coefficient	-.016	.057	.044	.054	-.077	-.055
	Sig. (2-tailed)	0.774	0.292	0.422	0.320	0.156	0.310
	N	341	341	341	341	341	341
Referral to specialism or specialist services	Correlation Coefficient	.086	.168	.061	.143	.118	.190
	Sig. (2-tailed)	0.111	0.002	0.258	0.008	0.029	0.0004
	N	341	341	341	341	341	341
Respiratory examination	Correlation Coefficient	-.012	.085	.087	.085	.038	.034
	Sig. (2-tailed)	0.829	0.116	0.109	0.116	0.480	0.527
	N	341	341	341	341	341	341
Respiratory rate	Correlation Coefficient	.013	.084	.024	.018	-.058	-.063
	Sig. (2-tailed)	0.813	0.121	0.666	0.742	0.285	0.248
	N	340	340	340	340	340	340
Skin and/or nail scrapings/samples	Correlation Coefficient	.045	.124	.072	.149	.042	.296**
	Sig. (2-tailed)	0.410	0.022	0.183	0.006	0.444	0.0000
	N	341	341	341	341	341	341
Spirometry	Correlation Coefficient	-.015	.004	.022	-.001	.032	.046
	Sig. (2-tailed)	0.778	0.940	0.687	0.986	0.561	0.402
	N	341	341	341	341	341	341
Sputum sample	Correlation Coefficient	.134	.209**	.084	.152	.118	.290**
	Sig. (2-tailed)	0.013	0.0001	0.121	0.005	0.030	0.0000
	N	341	341	341	341	341	341
Stool sample	Correlation Coefficient	.212**	.107	.126	.049	.142	.257**
	Sig. (2-tailed)	0.0001	0.049	.021	0.363	0.009	0.0000
	N	341	341	341	341	341	341
Swabs	Correlation Coefficient	.126	.211**	.082	.089	.090	.277**
	Sig. (2-tailed)	0.020	0.0001	0.133	0.099	0.097	0.0000
	N	341	341	341	341	341	341
Temperature	Correlation Coefficient	.023	.067	-.023	-.019	-.103	-.114
	Sig. (2-tailed)	0.676	0.215	0.672	0.733	0.057	0.035
	N	341	341	341	341	341	341
Throat examination	Correlation Coefficient	.071	.072	.153	.063	.082	.102
	Sig. (2-tailed)	0.193	0.183	0.005	0.243	0.129	0.061
	N	341	341	341	341	341	341
Urinalysis	Correlation Coefficient	.071	.104	.029	.050	-.004	.033
	Sig. (2-tailed)	0.193	0.056	0.592	0.356	0.947	0.548
	N	341	341	341	341	341	341

**. Correlation is significant at the 0.0002 level (2-tailed).

For peer review only

Supplemental Table C. Relationship between core capabilities of primary care and demographics of paramedics

			Clinical Supervision	Hours worked	Job Title	Length of time registered as a paramedic	Length of time in primary care	Level of Education	Prescribing Status	Salary
Spearman's rho	Communication and consultations	Correlation Coefficient	-.042	-.050	.138	.150	.051	.112	-.102	.127
		Sig. (2-tailed)	0.436	0.356	0.011	0.006	0.349	0.038	0.059	0.019
		N	341	341	341	341	341	341	341	341
	Practising holistically to personalise care and promote public and person	Correlation Coefficient	-.015	-.076	.123	-.016	.066	.115	-.162	.159
		Sig. (2-tailed)	0.779	0.164	0.023	0.767	0.226	0.034	0.003	0.003
		N	341	341	341	341	341	341	341	341
	Working with colleagues in primary care	Correlation Coefficient	-.128	-.189**	.066	.069	-.015	.048	-.107	.077
		Sig. (2-tailed)	0.018	0.0005	0.225	0.204	0.783	0.375	0.048	0.156
		N	341	341	341	341	341	341	341	341
	Maintaining an ethical approach and fitness to practice	Correlation Coefficient	-.095	-0.083	0.095	0.139	0.026	0.013	-0.055	.078
		Sig. (2-tailed)	0.081	0.125	0.080	0.010	0.629	0.812	0.312	0.153
		N	341	341	341	341	341	341	341	341
	Information gathering and interpretation	Correlation Coefficient	-.059	-.024	.219**	.062	.066	.125	-.123	.177
		Sig. (2-tailed)	0.280	0.658	0.0000	0.251	0.225	0.021	0.023	0.001
		N	341	341	341	341	341	341	341	341
	Clinical Examination and procedural skills	Correlation Coefficient	-.005	-.079	.079	.142	.106	.081	-.119	.102
		Sig. (2-tailed)	0.924	0.145	0.145	0.009	0.051	0.137	0.028	0.059
		N	341	341	341	341	341	341	341	341
	Making a diagnosis	Correlation Coefficient	.016	-.081	.174	.092	.251**	.196**	-.284**	.231**
		Sig. (2-tailed)	0.763	0.134	0.001	0.090	0.0000	0.0003	0.0000	0.0000
		N	341	341	341	341	341	341	341	341
	Managing medical and clinical complexity	Correlation Coefficient	-.047	-.145	.209**	.017	.170	.156	-.298**	.236**
		Sig. (2-tailed)	0.391	0.007	0.0001	0.750	0.002	0.004	0.0000	0.0000
		N	341	341	341	341	341	341	341	341
	Independent prescribing, medicines, and supply of pharmacotherapy	Correlation Coefficient	-.044	-.091	.334	.097	.357**	.403**	-.757**	.481**
		Sig. (2-tailed)	0.416	0.092	0.0000	0.075	0.0000	0.0000	0.0000	0.0000
		N	341	341	341	341	341	341	341	341
	Leadership and Management	Correlation Coefficient	.016	-.213**	.158	.045	.302**	.175	-.331**	.255**
		Sig. (2-tailed)	0.774	0.0001	0.003	0.403	0.0000	0.001	0.0000	0.0000
		N	341	341	341	341	341	341	341	341
	Education and Development	Correlation Coefficient	-.008	-.095	.105	.003	.143	.087	-.143	.106
		Sig. (2-tailed)	0.888	0.080	0.052	0.956	0.008	0.110	0.008	0.051
		N	341	341	341	341	341	341	341	341
	Research and evidence-based practice	Correlation Coefficient	.000	-.088	.095	.005	.033	.082	-.114	.133
		Sig. (2-tailed)	0.997	0.104	0.079	0.927	0.546	0.131	0.035	0.014
		N	341	341	341	341	341	341	341	341

**. Correlation is significant at the 0.0005 level (2-tailed).

For peer review only

Supplemental Table D. Correlations between the request and interpretation of blood tests and demogaphics of paramedics

			Clinical Supervision	Hours worked	Job Title	Length of time registered as a paramedic	Length of time in primary care	Level of Education	Prescribing Status	Salary
Spearman's rho	ACR	Correlation Coefficient	.093	.232	.094	-.021	.116	.123	.230	.236
		Sig. (2-tailed)	0.085	0.001	0.082	0.705	0.033	0.023	0.001	0.001
		N	341	341	341	341	341	341	341	341
	Amylase	Correlation Coefficient	.077	.118	.150	.059	.115	.198	.306	.266
		Sig. (2-tailed)	0.156	0.030	0.005	0.274	0.033	0.001	0.001	0.001
		N	341	341	341	341	341	341	341	341
	Anti CCP	Correlation Coefficient	.080	.047	.147	.035	.087	.165	.248	.195
		Sig. (2-tailed)	0.139	0.389	0.007	0.514	0.108	0.002	0.001	0.001
		N	341	341	341	341	341	341	341	341
	Autoimmune antibodies	Correlation Coefficient	.100	.084	.129	-.025	.041	.198	.197	.223
		Sig. (2-tailed)	0.065	0.120	0.017	0.649	0.448	0.001	0.001	0.001
		N	341	341	341	341	341	341	341	341
	B12	Correlation Coefficient	.125	.100	.158	-.005	.011	.149	.245	.273
		Sig. (2-tailed)	0.021	0.066	0.003	0.922	0.835	0.006	0.001	0.001
		N	341	341	341	341	341	341	341	341
	BNP/NT-proBNP	Correlation Coefficient	.153	.228	.095	.022	.109	.134	.278	.297
		Sig. (2-tailed)	0.005	0.001	0.081	0.687	0.044	0.013	0.001	0.001
		N	341	341	341	341	341	341	341	341
	CA125	Correlation Coefficient	.092	.161	.148	.005	.115	.186	.268	.257
		Sig. (2-tailed)	0.091	0.003	0.006	0.923	0.034	0.001	0.001	0.001
		N	341	341	341	341	341	341	341	341
	Calcium	Correlation Coefficient	.130	.165	.110	.055	.025	.166	.251	.214
		Sig. (2-tailed)	0.016	0.002	0.043	0.312	0.643	0.002	0.001	0.001
		N	341	341	341	341	341	341	341	341
	Coeliac screen	Correlation Coefficient	.077	.086	.146	-.054	.126	.197	.305	.326
		Sig. (2-tailed)	0.153	0.114	0.007	0.322	0.020	0.0003	0.001	0.001
		N	341	341	341	341	341	341	341	341
	CRP	Correlation Coefficient	.153	.189	.099	.009	.083	.108	.246	.296
		Sig. (2-tailed)	0.005	0.001	0.068	0.871	0.124	0.047	0.001	0.001
		N	341	341	341	341	341	341	341	341
	Drug levels	Correlation Coefficient	.025	.181	.011	.007	.037	.095	.172	.164
		Sig. (2-tailed)	0.641	0.001	0.836	0.895	0.498	0.079	0.001	0.002
		N	341	341	341	341	341	341	341	341
	ESR	Correlation Coefficient	.158	.114	.104	.091	-.028	.158	.189	.201
		Sig. (2-tailed)	0.003	0.036	0.055	0.094	0.600	0.003	0.001	0.001
		N	341	341	341	341	341	341	341	341
	Estosterone	Correlation Coefficient	.009	.090	.044	-.023	.013	.117	.134	.144
		Sig. (2-tailed)	0.869	0.097	0.415	0.671	0.806	0.030	0.013	0.008
		N	341	341	341	341	341	341	341	341
	FBC	Correlation Coefficient	.176	.125	.136	.009	.015	.167	.20	.267
		Sig. (2-tailed)	0.001	0.021	0.012	0.871	0.789	0.002	0.001	0.001
		N	341	341	341	341	341	341	341	341
	Free androgen index	Correlation Coefficient	-.054	.019	.024	-.044	.022	.129	.098	.104
		Sig. (2-tailed)	0.320	0.732	0.657	0.415	0.686	0.017	0.070	0.055
		N	341	341	341	341	341	341	341	341
	FSH/LH +/- prolactin	Correlation Coefficient	.005	.071	.089	.018	.032	.144	.161	.132
		Sig. (2-tailed)	0.920	0.189	0.101	0.746	0.552	0.008	0.003	0.015
		N	341	341	341	341	341	341	341	341
	Glandular fever screen	Correlation Coefficient	.061	.104	.168	.123	.223	.204**	.342**	.324
		Sig. (2-tailed)	0.264	0.056	0.002	0.023	0.001	0.0001	0.0000	0.001
		N	341	341	341	341	341	341	341	341
	Haematinics	Correlation Coefficient	.080	.201	.152	.069	.086	.144	.304	.279
		Sig. (2-tailed)	0.140	0.001	0.005	0.206	0.111	0.008	0.00100	0.001
		N	341	341	341	341	341	341	341	341
	HbA1c	Correlation Coefficient	.155	.142	.118	-.064	.002	.162	.202**	.249**
		Sig. (2-tailed)	0.004	0.009	0.029	0.235	0.971	0.003	0.0002	0.0000
		N	341	341	341	341	341	341	341	341
	Hepatitis	Correlation Coefficient	.076	.087	.127	-.015	.120	.156	.193	.234
		Sig. (2-tailed)	0.164	0.107	0.019	0.779	0.027	0.004	0.0003	0.001
		N	341	341	341	341	341	341	341	341
	HIV	Correlation Coefficient	.045	.069	.137	-.034	.154	.136	.192	.208
		Sig. (2-tailed)	0.407	0.205	0.011	0.526	0.004	0.012	0.001	0.001
		N	341	341	341	341	341	341	341	341
	Iron studies	Correlation Coefficient	.139	.115	.104	-.017	-.004	.135	.162	.198
		Sig. (2-tailed)	0.010	0.034	0.055	0.760	0.938	0.013	0.003	0.0002
		N	341	341	341	341	341	341	341	341
	LFT	Correlation Coefficient	.175	.129	.137	-.006	-.007	.165	.183	.247
		Sig. (2-tailed)	0.001	0.017	0.011	0.911	0.900	0.002	0.001	0.001
		N	341	341	341	341	341	341	341	341
	Lipid profile	Correlation Coefficient	.104	.071	.158	-.003	.077	.192	.278	.341
		Sig. (2-tailed)	0.054	0.192	0.003	0.957	0.157	0.0004	0.001	0.001
		N	341	341	341	341	341	341	341	341
	PSA	Correlation Coefficient	.123	.195	.137	-.008	.002	.205	.297	.293
		Sig. (2-tailed)	0.023	0.001	0.011	0.876	0.977	0.001	0.001	0.001
		N	341	341	341	341	341	341	341	341
	Rheumatoid factor	Correlation Coefficient	.082	.085	.154	.019	.081	.196	.248	.235
		Sig. (2-tailed)	0.130	0.117	0.004	0.727	0.137	0.001	0.001	0.001
		N	341	341	341	341	341	341	341	341
	SHBG	Correlation Coefficient	-.034	.008	.027	.009	.020	.155	.132	.101
		Sig. (2-tailed)	0.530	0.885	0.623	0.867	0.707	0.004	0.014	0.063
		N	341	341	341	341	341	341	341	341
	TFT	Correlation Coefficient	.133	.138	.154	.039	.081	.201**	.276	.276

For peer review only

	Sig. (2-tailed)	0.014	0.011	0.004	0.476	0.136	0.00019	0.001	0.001
	N	341	341	341	341	341	341	341	341
U&Es	Correlation Coefficient	.175	.146	.123	.006	-.007	.152	.185	.261
	Sig. (2-tailed)	0.001	0.007	0.024	0.906	0.898	0.005	0.001	0.001
	N	341	341	341	341	341	341	341	341
Urate	Correlation Coefficient	.141	.193	.122	.063	.133	.167	.256	.288
	Sig. (2-tailed)	0.009	0.001	0.025	0.244	0.014	0.002	0.001	0.001
	N	341	341	341	341	341	341	341	341
Vitamin D	Correlation Coefficient	.142	.090	.095	-.066	-.027	.147	.183	.192
	Sig. (2-tailed)	0.009	0.098	0.078	0.225	0.623	0.006	0.001	0.000
	N	341	341	341	341	341	341	341	341

**. Correlation is significant at the 0.0002 level (2-tailed).

For peer review only

Supplemental Table E. Associations between prescribing status and clinical skills assessments in primary care

Clinical Assessments	Value	df	Asymptotic Significance (2-sided)
Digital rectal examination	68.059	4	215
Eye examination	24.228	4	<.001
Imaging	15.861	4	0.003
Lymphadenopathy	28.75	4	<.001
Mental health examination	14.886	4	0.005
Otoscopy	21.312	4	<.001
Referrals to specialism or specialist services	14.886	4	0.003
Skin samples	37.529	4	<.001
Sputum samples	31.322	4	<.001
Stool samples	24.25	4	<.001
Swabs	32.213	4	<.001

For peer review only

STROBE Statement—Checklist of items that should be included in reports of ***cross-sectional studies***

	Item No	Recommendation	Page No
**Title and abstract**	1	(*a*) Indicate the study's design with a commonly used term in the title or the abstract	1,3
		(*b*) Provide in the abstract an informative and balanced summary of what was done and what was found	3
**Introduction**			
Background/rationale	2	Explain the scientific background and rationale for the investigation being reported	5,6
Objectives	3	State specific objectives, including any prespecified hypotheses	6
**Methods**			
Study design	4	Present key elements of study design early in the paper	3,4,5, 7
Setting	5	Describe the setting, locations, and relevant dates, including periods of recruitment, exposure, follow-up, and data collection	6,7
Participants	6	(*a*) Give the eligibility criteria, and the sources and methods of selection of participants	7; Supp 1
Variables	7	Clearly define all outcomes, exposures, predictors, potential confounders, and effect modifiers. Give diagnostic criteria, if applicable	4, 15,16
Data sources/ measurement	8*	For each variable of interest, give sources of data and details of methods of assessment (measurement). Describe comparability of assessment methods if there is more than one group	7
Bias	9	Describe any efforts to address potential sources of bias	18,19
Study size	10	Explain how the study size was arrived at	7
3Quantitative variables	11	Explain how quantitative variables were handled in the analyses. If applicable, describe which groupings were chosen and why	7
Statistical methods	12	(*a*) Describe all statistical methods, including those used to control for confounding	7
		(*b*) Describe any methods used to examine subgroups and interactions	NA
		(*c*) Explain how missing data were addressed	NA
		(*d*) If applicable, describe analytical methods taking account of sampling strategy	7
		(*e*) Describe any sensitivity analyses	NA
**Results**			
Participants	13*	(a) Report numbers of individuals at each stage of study—eg numbers potentially eligible, examined for eligibility, confirmed eligible, included in the study, completing follow-up, and analysed	8
		(b) Give reasons for non-participation at each stage	NA

For peer review only - http://bmjopen.bmj.com/site/about/guidelines.xhtml

		(c) Consider use of a flow diagram	NA
Descriptive data	14*	(a) Give characteristics of study participants (eg demographic, clinical, social) and information on exposures and potential confounders	8
		(b) Indicate number of participants with missing data for each variable of interest	8
Outcome data	15*	Report numbers of outcome events or summary measures	8-16
Main results	16	(*a*) Give unadjusted estimates and, if applicable, confounder-adjusted estimates and their precision (eg, 95% confidence interval). Make clear which confounders were adjusted for and why they were included	8-16; Supp 2
		(*b*) Report category boundaries when continuous variables were categorized	NA
		(*c*) If relevant, consider translating estimates of relative risk into absolute risk for a meaningful time period	NA
Other analyses	17	Report other analyses done—eg analyses of subgroups and interactions, and sensitivity analyses	NA
**Discussion**			
Key results	18	Summarise key results with reference to study objectives	17,18
Limitations	19	Discuss limitations of the study, taking into account sources of potential bias or imprecision. Discuss both direction and magnitude of any potential bias	18,19
Interpretation	20	Give a cautious overall interpretation of results considering objectives, limitations, multiplicity of analyses, results from similar studies, and other relevant evidence	17, 18, 20
Generalisability	21	Discuss the generalisability (external validity) of the study results	17,18
**Other information**			
Funding	22	Give the source of funding and the role of the funders for the present study and, if applicable, for the original study on which the present article is based	22

*Give information separately for exposed and unexposed groups.

Note: An Explanation and Elaboration article discusses each checklist item and gives methodological background and published examples of transparent reporting. The STROBE checklist is best used in conjunction with this article (freely available on the Web sites of PLoS Medicine at http://www.plosmedicine.org/, Annals of Internal Medicine at http://www.annals.org/, and Epidemiology at http://www.epidem.com/). Information on the STROBE Initiative is available at www.strobe-statement.org.

For peer review only
